# Novel alloxazine analogues: design, synthesis, and antitumour efficacy enhanced by kinase screening, molecular docking, and ADME studies

**DOI:** 10.1080/14756366.2024.2398551

**Published:** 2024-09-17

**Authors:** Doaa Samaha, Sawsan Mahmoud, Mosaad Sayed Mohamed, Rokaia S. Abdullah, Nageh A. Abou Taleb, Tomohisa Nagamatsu, Hamed I. Ali

**Affiliations:** a Pharmaceutical Chemistry Department, Faculty of Pharmacy, Helwan University, Helwan, Egypt; b Institute of Chemistry, Humboldt-Universität zu Berlin, Berlin, Germany; c Pharmaceutical Organic Chemistry Department, Faculty of Pharmacy, Helwan University, Helwan, Egypt; d Environment Division, National Institute of Oceanography and Fisheries (NIOF), Alexandria, Egypt; e Department of Pharmaceutical Sciences, Irma Lerma Rangel School of Pharmacy, Texas A&M University, College Station, TX, USA; f Laboratory of Curative Creation Study for Geriatric-diseases Prevention, Faculty of Pharmacological Sciences, Sojo University, Kumamoto, Japan

**Keywords:** Alloxazine, antitumor, molecular docking, kinase profiling, ADME

## Abstract

This study describes the development of novel alloxazine analogues as potent antitumor agents with enhanced selectivity for tumour cells. Twenty-nine out of 45 newly compounds were investigated *in vitro* for their growth inhibitory activities, against two human tumour cell lines, namely, the human T-cell acute lymphoblastoid leukaemia cell line (CCRF-HSB-2) and human oral epidermoid carcinoma cell line (KB), and the antitumor agent “Ara-C” was used as a positive reference in this investigation. Compounds **9e** and **10J** were the highest among their analogues, against both tumour cell lines (CCRF-HSB-2 and KB). Correlation analyses demonstrated a strong relationship between the IC_50_ values and AutoDock binding free energy or calculated inhibition (*K_i_*). The study delves into structure–activity relationships (SARs) through advanced modelling tools integrated with structure-based drug design (SBDD) using GOLD 5.2.2, AutoDock 4.2, and Accelrys Discovery Studio 3.5. Physicochemical properties, pharmacokinetics, drug-likeness, and toxicity predictions of the most potent alloxazine derivatives were conducted using ProTox-II and Swiss ADME for effective antitumor agents with improved selectivity.

## Introduction

Cancer research occupies great importance in clinical research, as cancer is considered one of the most deadly diseases around the world^1^. There are two main types of cancer treatment (local and systematic therapy). Local treatments (surgery and radiation therapy) are directed to a specific organ or limited area of the body, where systemic treatments (chemotherapy, hormonal therapy, and immunotherapy) are therapies and drugs that spread throughout the body to treat or destroy cancer cells. Chemotherapy is the most common cancer treatment. Chemotherapy is the use of anti-cancer drugs to kill cancer cells, but the challenge is to kill the malignance cells only and leave the other normal cells.

Tyrosine kinases are a subgroup of the larger class of protein kinases. The term kinase describes a large family of enzymes that are responsible for catalysing the transfer of a phosphoryl group from a nucleoside triphosphate donor, such as ATP, to an acceptor molecule. Tyrosine kinases catalyse the phosphorylation process of tyrosine residue in proteins^2^. The phosphorylation of tyrosine residues in turn causes a change in the function^2^ of the protein that they are contained in, so we conclude that protein-tyrosine kinases (PTKs) play a vital role in the control of most essential cellular processes, such as cell migration, cell viability, cell metabolism as well as cell proliferation and differentiation^3^.

Different classes of compounds were investigated for their binding affinities into different protein tyrosine kinases (PTKs)^4–7^. These compounds include many flavin and alloxazine analogues, which were developed in our previous publications^8–11^. Flavin-N-oxides have been used in recent time for the treatment of solid tumours, non-solid tumour masses, and non-small cell lung cancers involving *in situ* activator mixed with the flavin-N-oxide for some time, which led to destruction of the DNA in cancer cells without substantial destruction to the DNA of normal cells^12^.

This study was prompted by these circumstances to seek to synthesise more 2-methylthio and 2-(substituted amino) alloxazine analogues with different substituents to investigate their antitumor effects and to improve potent bio reductive and selective agents towards tumour cells.

In this study, we design and synthesis various compounds including 10-alkyl-2-amino-flavin-5-oxide, 2-deoxo-2-methylthio-alloxazin-e-5-oxid. Furthermore, lead optimisation of the potentially active ­compounds is planned to be done via further structural modification, especially at C_2_-position which is considered as the main significant site influencing the antitumor potency and via N^3^-alkylation of alloxazine analogues. All the designed synthetically proposed derivatives are planned to be subjected to *in vitro* biological assays against different tumour cell lines (CCRF-HSB-2, and KB cells).

To achieve this goal, mainly we have to investigate the structure–activity relationship (SAR) of these proposed derivatives with modification of the structural features to improve their antitumor potencies. This requires the application of the aforementioned approaches of computer-aided drug design (CADD) and its main branch, structure-based drug design (SBDD), as powerful approaches aim to increase the speed and efficiency of the drug discovery process^13^. Also, protein kinase inhibitors (PKIs) have recently been reported as very potent against a wide range of cancers, docking of the synthesised and rationally designed derivatives into different PTKs has to be investigated as a virtual screening tool^14^.

## Results

### Chemistry

**Scheme 1.**
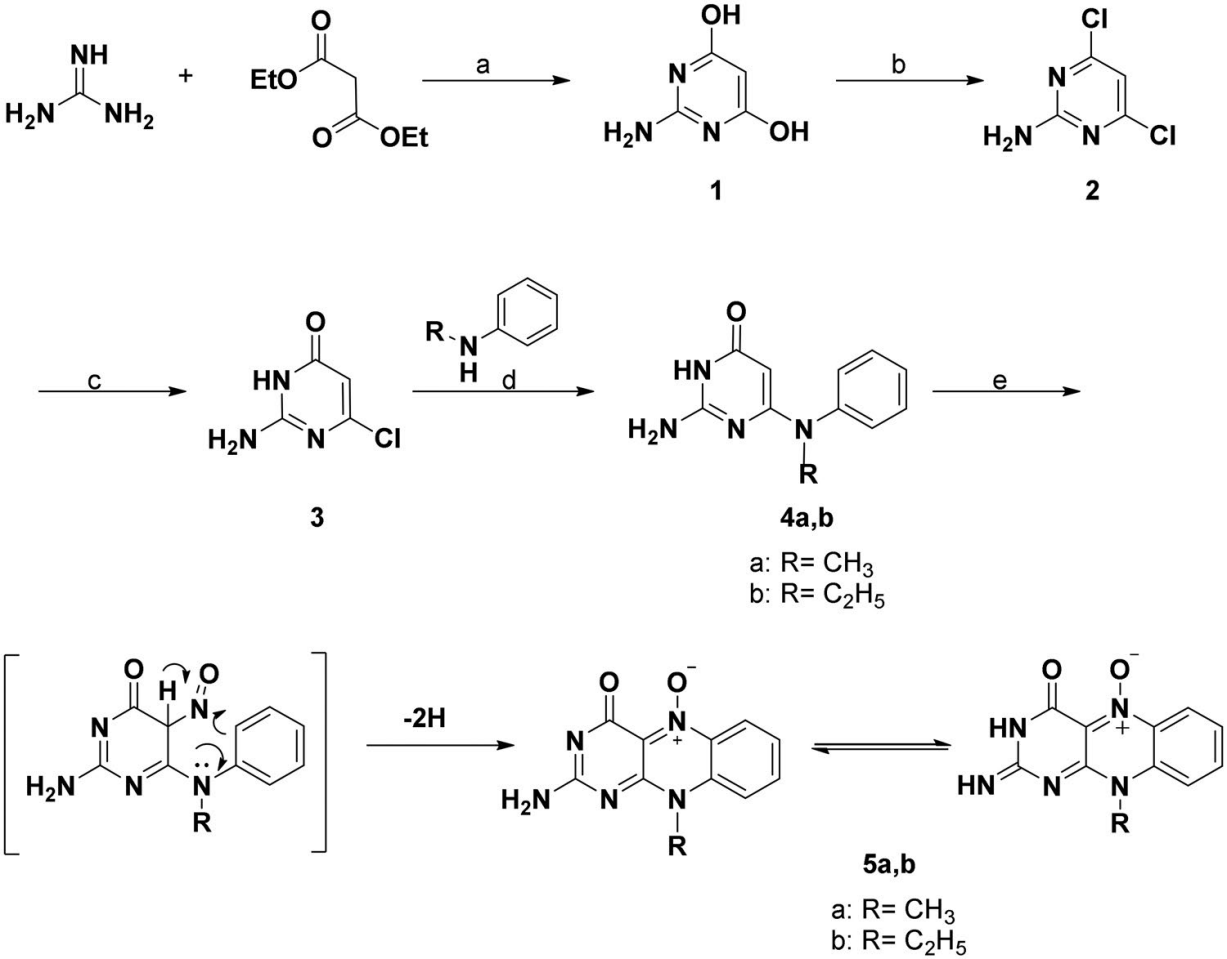
General procedure for the preparation of 2-amino-2-deoxoflavin-5-oxides (**5a,b**). Reagent and conditions: (a) NaOEt, reflux, 2h; (b) POCl_3_, triethylamine, reflux, 5–7 h; (c) NaOH (1.0 N), reflux, 5 h; (d) fusion at 190 °C, 7 h; (e) NaNO_2_, AcOH, 10–15 °C, 2–5 h.

**Scheme 2.**
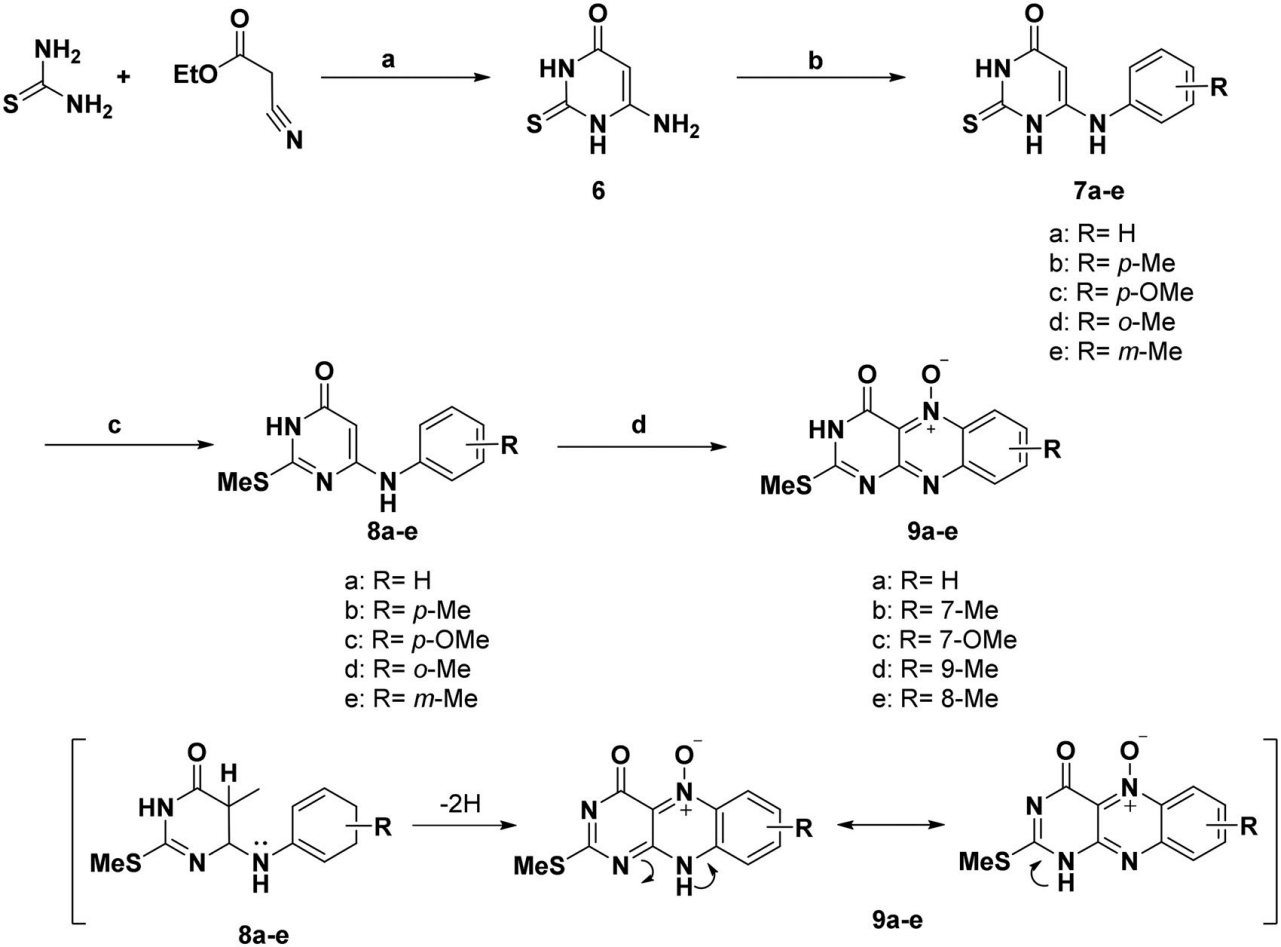
General procedure for the synthesis of 6-N-anilino-2-thioxopyrimidine-4-ones (**7a–e**), 6-N-anilino-2-methylthio-pyrimidin-4-ones (**8a–e**), and 2-deoxo-2-thio-methyl-alloxazin-5-oxide (**9a–e**). Reagents and conditions: (a) NaOEt, reflux, 2 h; (b) ArNH_2_/ArNH_3_^+^ Cl^−^, 170 °C, 9.0 h; (c) MeI, NaOH, 0–5 °C, 30 min; (d) NaNO_2_, AcOH, 10–15 °C, 2–5 h.

**Scheme 3.**
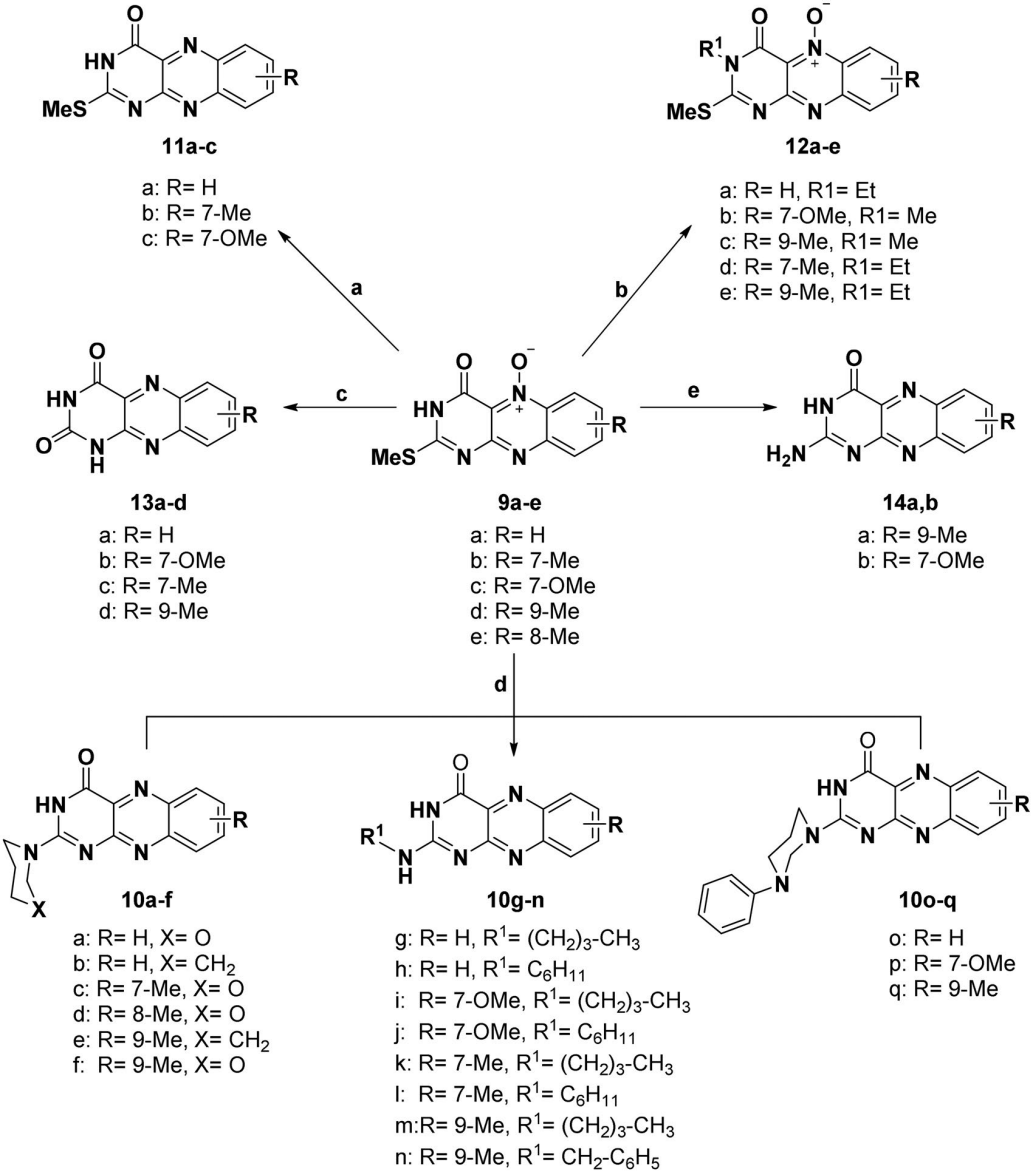
General procedure for the preparation of 2-substituted amino alloxazines (**10a–q**), 2-methylthio-alloxazines (**11a–c**), 3-alkyl-2-methylthioalloxazin-5-oxides (**12a–c**), 2,4-dioxo-aloxazine-5-oxides (**13a–d**), and 2-amino-alloxazines (**14a,b**). *Reagent and conditions*: (a) Na_2_S_2_O_4_, H_2_O, r.t., 12 h; (b) R^1^I, DMF, K_2_CO_3_, r.t., 10 h; (c) 5 N HCl, reflux, 15 h; (d) appropriate amine, DMF, reflux 5–12 h; (e) CH_3_COONH_4_, 160–165 °C, 0.5–1.5 h.

**Scheme 4.**
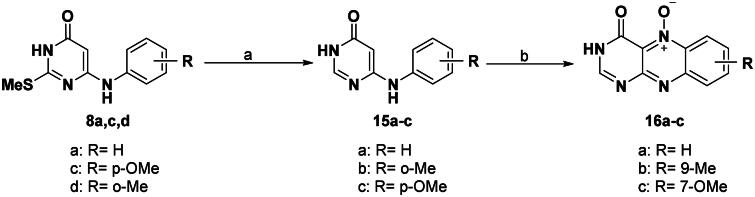
General method for the preparation of 6-(*N*-anilino) pyrimidin-4(3*H*)-ones (**15a–c**) and 4-oxo-3,4-dihydro-alloxazine-5-oxides (**16a–c**). *Reagent and conditions*: (a) Raney Ni, absolute EtOH, reflux 0.5–1.5 h; (b) NaNO_2_, AcOH, 10–15 °C, 4–8 h.

In this investigation, it is of interest to prepare novel flavin-5-oxide and alloxazin-5-oxide derivatives of expected antitumor activity. The synthetic techniques utilised for the synthesis of various compounds namely: 2-aminoflavin-2-deoxy-5-oxides; 2-amino-10-alkyl-4-oxo-4,10-dihydrobenzo[g]pteridin-5-oxide; (**5a,b**), 6-N-anilino-2-thioxopyrimidin-4-ones (**7a–e**), 6-anilino-2-methylthio-pyrimidine-4-ones (**8a–e**), 2-deoxo-2-thio-methyl-alloxazin-5-oxide (**9a–e)**, 2-(substituted amino)benzo[g] pteridin-4(3H)-on-es (**10a–q**), 2-deoxo-2-thiomethyl-alloxazines (**11a–c**), N^3^-alkyl-2-methylthio-alloxaz-in-5-oxides (**12a–e**), 2,4-dioxo-1,2,3,4-tetrahydrobenzo[g]pteridin-5-oxides (**13a–d**), 2-amino-benzo[g]pteridin-4(3H)-ones (**14a,b**), 6-(substituted phenylamino) pyrimidine-4(3H)-ones (**15a–c**), and 4-oxo-3,4-dihydrob-enzo[g]pteridin-5-oxides (**16a–c**), as illustrated in Schemes 1–4.

The 2-aminopyrimidine-4,6-diol (**1**) was involved as a precursor for the synthesis of the key substance 2-amino-6-chloropyrimidin-4(3H)-one (**3**) and it was obtained by reaction of guanidine hydrochloride with diethyl malonate in sodium ethoxide solution ­according to the reported procedure^15^, followed by chlorination with phosphoryl chloride at 85–95 °C in the presence of an acid scavenger; triethylamine without the use of a solvent to afford 4,6-dichloropyrimidin-2-amine (**2**)^16^, followed by partially basic hydrolysis by reflux with sodium hydroxide for 5 h to give 2-amino-6-chloropyrimidin-4(3H)-one (**3**)^17^. The key intermediates 6-(N-monoalkylanilino)-2-aminothiopyrimidin-4(3H)-ones (**4a,b**) were synthesised according to the reported procedure^18^ by fusion at 190 °C of 2-amino-6-chloro-pyrimidine-4 (3H)-one (**3**) with appropriate N-alkyl anilines for 7 h followed by treatment of the cooled residue with ether to afford 67–84% yield of 6-(N-monoalkylanilino)-2-aminothiopyrimidin-4(3H)-ones (**4a,b**) as shown in Scheme 1. This reaction proceeds via the nucleophilic substitution of the 6-choro atom by N-alkyl anilines. The intended 2-deoxy-2-aminoflavin-5-oxides (**5a,b**) were prepared according to the reported procedure^19^ by nitrosative cyclisation of 2-amino-6-(N-alkylanilino)pyrimidin-4(3H)-ones (**4a,b**).Using 2–4 equiv. of NaNO_2_ in AcOH at 10–15 °C and stirring at room temperature for 2–5 h yielded red crystals with 62–66% (Scheme 1).

The target compounds (**5a,b**) tend to exhibit tautomeric equilibrium which was confirmed by using spectral analysis. Where, 2-amino-10-alkyl-4-oxo-4,10-dihydro-alloxazine-5-oxides (**5a,b**) were found to predominate in DMSO solution at 25 °C as 2-imino-10-methyl-4-oxo-4,10-dihydrobenzo[g]pteridin-5-oxide tautomer^20^ as shown in Scheme 1.

This phenomenon was previously reported in detail by our research group^11^^,^^21^. The coalescence phenomenon of imino and amino tautomers in 2-monoalkyl amino derivatives was observed in ^1^H NMR spectra at 100 °C, favouring the imino form at higher temperatures. In contrast, benzylidene hydrazino derivatives showed no coalescence due to resonance stabilisation^21^. Additionally, amide-imidol tautomerism was noted in 2-deoxo-2-(4-phenylpiperazin-1-yl)-5-deazaalloaxazines, with the imidol tautomer predominating, evidenced by specific NMR and IR spectral shifts^11^.

The UV absorption spectra of the 2-deoxo-2-aminoflavin-5-oxides (**5a,b**) exhibit four absorption maxima at 244–250, 274, 334–340, and 452–456 nm. Owing to the absorption peak in the longest wavelength (452–456 nm), these two compounds have a red appearance.

This was supported for compound **5a** by the evidence of two singlet ^1^H NMR signals at 4.24 and 9.9 ppm which are exchangeable with D_2_O. These two singlet signals are attributed to 2-imino ═NH and N^3^H protons, respectively. Whereas the corresponding two singlet D_2_O exchangeable signals of compound **5b** were located at 4.0 and 10.0 ppm, respectively.

Moreover, the IR spectra of compound **5a** showed stretching bands at 3287 and 3420 cm^−1^ which are corresponding to 2-imino ═NH and N^3^H protons, respectively. Whereas compound **5b** exhibited the stretching bands at 3201 and 3435 cm^−1^ of 2-imino ═NH and N^3^H protons, respectively.

The required 6-amino-2-thiouracil (**6**) was prepared through the reaction of thiourea and ethyl cyanoacetate in sodium ethoxide according to the known procedure^22–24^. The 2-thiouracil is an example of a six-membered ambident heterocyclic system possessing several possible tautomeric structures but found predominantly in the oxothion form^25^^,^^26^.

Owing to the higher reactivity of anilines, the requisite starting materials, 6-anilino-2-methylthio-pyrimidine-4-ones (**8a–e**) which are the key intermediate for synthesis of 2-deoxo-2-methylthio-alloxazin-5-oxide cannot be prepared directly by reaction of anilines with 6-amino-2-methylthiopyrimidine-4(3H)-one^27^^,^^28^. Where both of 6-amino and 2-methylthio groups were replaced with aniline moiety. Therefore, compounds (**8a–e**) were prepared in two steps, first by amine exchange route using fusion of 6-amino-2-thiouracil with anilines in the presence of the corresponding salts of aniline chloride at 170 °C to afford 38–77% yield of 6-N-anilino-2-thioxopyrimidine-4-ones (**7a–e**) as shown in Scheme 2.

The term "exchange amination" is used to refer to the exchange of one amino group by another amine including the exchange of a hydrazine group^29^. Pyrimidines having primary amino or imino functions on a carbon adjacent to endocyclic nitrogen are readily available for nucleophilic displacement by exchange amination. Several reactions of primary amines with pyrimidinylamines were proceeded by an acid catalyst and two reactions were performed to determine whether an acid catalyst was required, and it was discovered that the exchange amination reaction in the absence of an acid catalyst did not give a consistent result. It is believed that the acid functions both as a catalyst and as a reagent, with aniline forming anilinium ion; and that the presence of some anilinium ion favours the desired reaction, although the precise mechanism is not understood. The acid can be supplied to the reaction mixture in a variety of ways. It can be added separately, or it can be supplied as a salt of the aniline. In our work, we used anilinium hydrochloride salt as an acid catalyst, and better results were obtained with two molecular proportions of the appropriate aniline and about one molecular proportion of the corresponding anilinium chloride per molecular proportion of the 6-amino-2-thiouracil (**6**). It has now been found that the formation of side products is enhanced by higher anilinium hydrochloride concentrations. Also, better results were generally obtained at temperatures of 170 °C. These compounds (**7a–e**) were synthesised by modification of the procedure in literature^30^ to get more pure products. In our proposed procedure, the obtained crude product was purified by dissolving it in a basic solution with heating in a steam bath for one hour, followed by cooling and filtration to get rid of the aniline side product, and then the filtrate was reprecipitated by 10% hydrochloric acid to get more pure compounds. Compound 2,3-dihydro-6-(*o*-tolylamino)-2-thioxopyrimidin-4(1*H*)-one (**7d)**, in which the methyl group in ortho position, was obtained in yield (69%) better than other analogues. Whereas compound 2,3-dihydro-6-(*o*-tolylamino)-2-thioxopyrimidin-4(1*H*)-one (**7e)** where the methyl group in meta position was obtained in the lowest yield (39%).

The IR, ^1^H NMR, UV/vis spectra, and elemental analysis, were used to elucidate the newly assigned structures. The UV absorption spectra of the 2,3-dihydro-6-(N-anilino)-2-thioxopyrimidin-4(1H)-ones (**7a–e**) revealed one absorption *λ* maximum at 272–278 nm which is attributed to their aromatic aniline ring. The IR spectra of compounds (**7d,e**) exhibited stretching bands at 3324–3288, 3185–3170, 3143–3090, and 1663–1633 cm^−1^, attributed to the 6-NH, 1-NH, 3-NH, and C═O groups, respectively. The structures of the products (**7d,e**) were confirmed by the presence of the resonance of an equivalent proton at the 5-position as a singlet signal at *δ*_H_ 4.45 and 4.94 ppm in ^1^H NMR spectra as illustrated.

The second step reaction included methylation of the sodium salt of 6-N-anilino-2-thioxopyrimidin-4-ones (**7a–e**) by CH_3_I in alkaline pH in an ice bath to afford analogues of 2-methylthio (**8a–e**) in a good yield of 69–94%. The reported studies^31^ showed that when 2-thiouracils were treated with an alkylating agent, the attack was virtually always at sulphur which is the most nucleophilic atom. Therefore, for N-alkylation sulphur should be blocked in the initial step.

The UV spectra of the 6-N-anilino-2-methylthio-3,4-dihydropyrimidine-4-ones (**8d,e**) exhibited one absorption maxima at *δ* 246–258 nm together with one absorption shoulder at *λ*300–304 nm, which is attributed to their aromatic aniline ring in addition to the further unsaturation in the dihydro-pyrimidinone ring. Compounds (**8d,e**) exhibited a bathochromic shift in comparison with compounds (**7d–e**) due to the conjugation change that occurred in the ring system where the thioxo group converted to thiomethyl group which retained the conjugation system of the pyrimidine ring.

The IR spectra of compounds (**8d,e**) exhibited stretching bands at *λ*3259–3238, 3139–3138, and 1631–1627 cm^−1^ corresponding to the 6-NH, 3-NH, and C═O groups, respectively.

The structures of the products (**8a–e**) were confirmed and differentiated from their precursors by the presence of methyl group at position 2 as a singlet signal at *δ*_H_ 2.43–2.52 in ^1^H NMR spectra and also the downfield shift of their singlet signals of proton at the 5-position to *δ*_H_ 4.80–5.34 in ^1^H NMR spectra in comparison to those of their precursors (**7d,e**) being *δ*_H_ 4.45 and 4.94.

In this investigation, we try to prepare the computationally designed analogues of the proposed highest binding affinities, namely 2-deoxo-2-methylthioalloxazine-5-oxides (**9a–e**). These designed derivatives were prepared from the key intermediate 6-N-anilino-2-methyl-thio-3,4-dihydropyrimidin-4-ones (**8a–e**) by a facile method of interaction (Scheme 2).

The 2-thioxo-alloxazine-5-oxides were not successfully synthesised directly from 6-N-anilino-2-thioxopyrimidin-4-ones (**7a–e**). This may be assigned to the oxidative dimerisation of 2-thioxo derivatives. Therefore, the 2-thioxo moiety should be protected by methylation to give 6-N-anilino-2-methylthio-3,4-dihydropyrimidin-4-ones (**8a–e**) which undergo nitrosative cyclisation involving 2–4 equiv. of NaNO_2_ in AcOH at 10–15 °C and then stirring at r.t. for 2–5 hours. After addition of sodium nitrite to the cold reaction mixture, the obtained greenish-yellow nitroso intermediates were dissolved in AcOH by heating in a warm water bath to enhance the prompt cyclisation to obtain yellow 2-thiomethyl-alloxazine-5-oxide derivatives (**9a–e**) with 62–89% yields (Scheme 2).

The IR, ^1^H NMR spectra, UV/vis, and elemental analysis were used for the elucidation of the newly assigned structures, where the UV/vis absorption spectra *λ* of compounds (**9d–e**) exhibited four absorption *λ* maxima at 272, 294–296, 360–364, and 442–448 nm. All compounds of **9a–e** showed a yellow appearance attributed to the presence of absorption *λ* maximum at 442–448 nm in the long wavelength.

The IR spectra of compounds (**9d,e**) exhibited stretching bands at 3184–3176 and 1695–1687 cm^−1^ which correspond to the 3-NH and C═O groups, respectively. The cyclised 2-deoxo-2-thiomethyl-alloxazin-5-oxides (**9d,e**) were mainly differentiated from their non-cyclised precursors (**8d,e**) by the disappearance of the singlet signal of the proton at *δ*_H_ 4.80–5.30 ppm of the 5-position in their ^1^H NMR spectra with the loss of one aromatic proton by the oxidative cyclisation at ortho position. Additionally, products (**9d,e**) exhibited a slight downfield shift of their ^1^H NMR singlet signal of 2-SMe moieties being at 2.62–2.63 ppm owing to the newly cyclised conjugated tricyclic alloxazine ring. While that of **8d,e** were located at 2.43–2.52 ppm, respectively. Additionally, ^CZ^NMR singlet signals of 3-NH were shifted from *δ*_H_ 11.7 and 11.9 ppm in **8d,e** to 12.80 ppm in the cyclised derivatives **9d,e**.

The 2-(substituted amino)-2-deoxo-al-loxazines (**10a–q**) were synthesised by a simple method including reflux of 2-deoxo-2-methylthioalloxazin-5-oxides (**9a–e**) with appropriate amines such as morpholine, piperidine, cyclohexylamine, butylamine, N-phenylpiperazine, and benzylamine in DMF under reflux for 5–12 h as shown in Scheme 3. All products (**10a–q)** were collected as orange or yellow needles in yields of 44–87%. This replacement was accompanied by deoxygenation of alloxazine-5-oxide to alloxazine as reported^19^. For all the aforementioned reactions which included treatment with different amines, the excess volatile amines were eliminated by concentration *in vacuo*.

The IR, ^1^H NMR, UV/vis spectra, and elemental analysis were used for elucidation of the newly attributed structures. Considering the ^1^H NMR spectra, the 2-(substituted amino)-2-deoxo-alloxazines (**10a–q**) are characterised by the disappearance of the strong characteristic singlet signal of the 2 CH_3_S group which was attributed for products (**9a–e**) at *δ*_H_ 2.62–2.63 ppm and a singlet signal of the 3-NH at *δ*_H_ 9.40–13.50 ppm and the aromatic protons of fused benzene ring appeared at *δ*_H_ 6.75–8.20 ppm. The ^1^H NMR spectra for 2-morphilino substituted compounds (**10a**, **10c**, **10d**, and **10f**) showed multiplet peaks at *δ*_H_ (3.70–3.74) and (3.75–3.85) ppm corresponding to 4H of 3′ and 5′-NCH_2_ and of 2′ and 6′-OCH_2_, respectively, for compound **10f** (Figure S1). The IR spectra of compounds (**10a**, **10c**, **10d**, and **10f**) showed stretching bands at 3148–3195 and 1680–1700cm^−1^ corresponding to the 3-NH and C═O groups, respectively.

The UV/vis absorption spectra of compounds (**10a** and **10d**) exhibited four absorption maxima at 222, (270–272), (338–342), and (438–440) nm, together with an absorption shoulder at 286 nm. For compounds (**10c** and **10f**), five absorption maxima at (224–226), (270–272), 286, 346, and (428–432) nm together with an absorption shoulder at 250–254 nm. These additional bands for compounds **10c** and **10f** may be due to electron donating + I effect of the methyl group at 7 and 9 positions, which changes the electronic environment.

The ^1^H NMR spectra for 2-piperidinyl substituted compound (**10b** and **10e**) showed multiplet peaks at *δ*_H_ 1.63–1.78 and 3.75–3.90 ppm corresponding to 6H of 3′,4′ and 5′-CH_2_ and 4H of 2′ and 6′-NCH_2_, respectively. The IR spectra of compounds (**10b** and **10e**) showed stretching bands at 3152–3179 and 1682–1701 cm^−1^ corresponding to the 3-NH and C═O groups, respectively. The UV/vis absorption spectra of compounds (**10b,e**) exhibited five absorption maxima at 216–224, 272, 290–294, 336–348, and 430–436 nm, together with an absorption shoulder at 252–254 nm.

The ^1^H NMR spectra for 2-butylamino substituted compound (**10g**, **10i**, **10k** and **10m**) showed triplet peak at *δ*_H_ 0.89–1.42 for 3H of 2-NH-(CH_2_)_3_-***CH_3_***, hextet peak at *δ*_H_ 1.39–1.90 for 2H of 2-NH-(CH_2_)_2_-***CH_2_***-CH_3_, quintet peak at *δ*_H_ 1.57–2.20 for 2H of 2-NH-CH_2_-***CH_2_***-CH_2_-CH_3_, multiplet peak at *δ*_H_ 3.30–3.96 for 2H of 2-NH-***CH_2_***-(CH_2_)_2_-CH_3_ and broad singlet peak at *δ*_H_ 6.7–7.67 ppm for 1H of 2-NH as amplified by compound **10i** (Figure S2).

The IR spectra of compounds (**10g**, **10i**, **10k**, and **10m**) showed stretching bands at 3394–3426, 3220–3246, and 1680–1700 cm^−1^ corresponding to the exocyclic 2-NH, 3-NH, and C═O groups, respectively.

**Figure 1.**
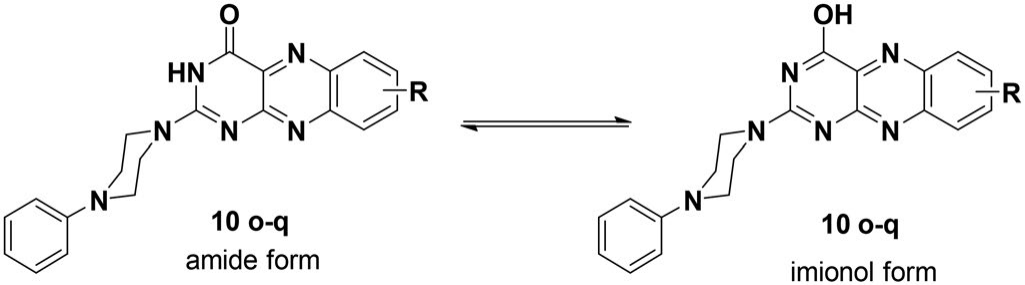
The amide-iminol tautomerism observed in compounds 2-(4-phenylpiperazin-1-yl)-alloxazine analogues (**10o–q**).

Interestingly, in the ^1^H NMR spectra for 2-(4-phenylpiperazin-1-yl)-alloxazine analogues (**10o–q**), the phenomenon of amide-iminol tautomerism was noticed (Figure 1).

**Figure 2.**
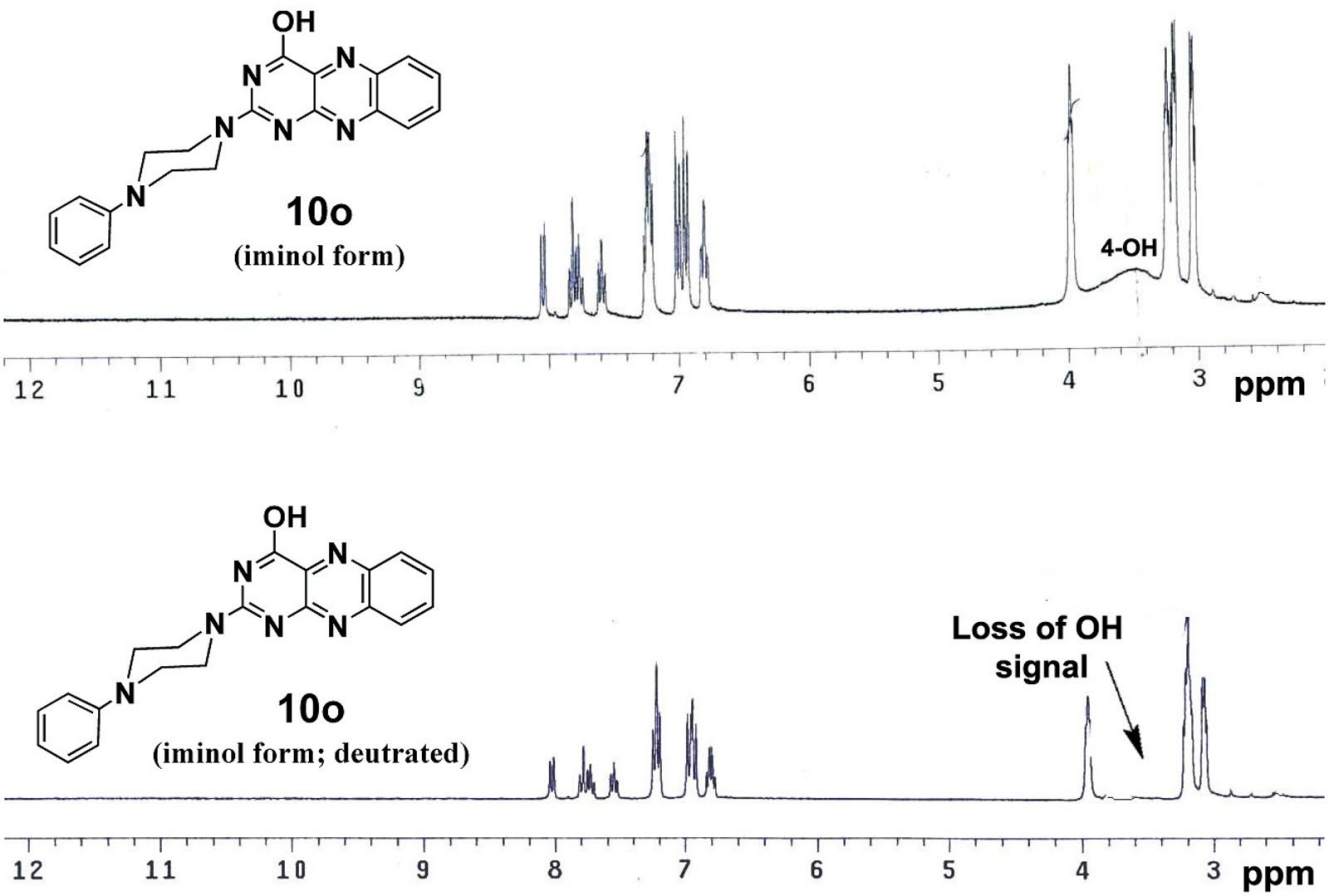
The NMR spectrum of compound 2-(4-phenylpiperazin-1-yl) alloxazine **(10o)** predominates in its iminol tautomeric form. The above spectrum represents ^1^H NMR in DMSO exposing the 4-OH broad signal, and the lower spectrum in deuterated DMSO (DMSO-*d*_6_), where the 4-OH proton disappeared.

Where the iminol tautomer was predominate in DMSO at 25 °C. These findings were supported by the presence of defined D_2_O exchangeable singlet at *δ*_H_ 3.32–3.45 ppm in the ^1^H NMR spectra which belonged to 4-OH and the disappearance of singlet signal for 3-NH proton which can be detected easily in another analogues (Figure 2). Moreover, this was confirmed by the disappearance of NH and C═O bands in the IR spectrum, and they were displaced by the presence of the OH stretching band at 3426–3436 m^−1^.

**Figure 3.**
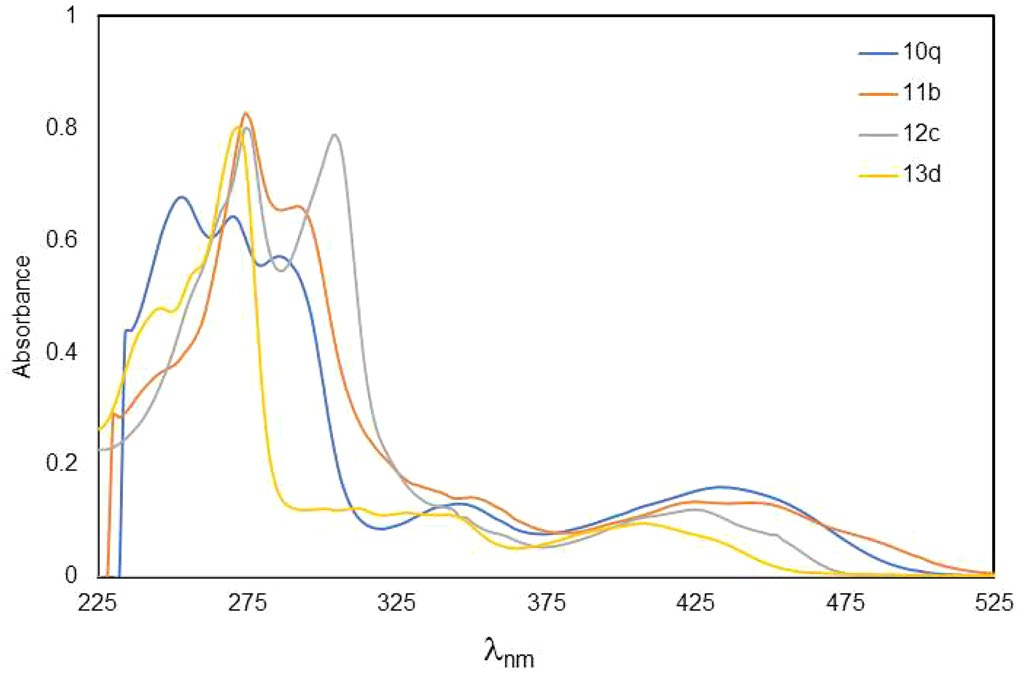
UV–vis spectra of 9-methyl-2-(4-phenylpiperazin-1-yl)-alloxazine(**10q**), 7-methyl-2-(methylthio)-alloxazine (**11b**), 3,9-dimethyl-2-deoxo-2-methylthio-alloxazin-5-oxide (**12c**), and 2,4-dioxo-9-methyl-1,2,3,4-tetrahydro-alloxazine-5-oxide (**13d**).

The UV/vis absorption spectra of compounds (**10o** and **10q**) exhibited five absorption maxima at 252–254, 270, 284–286, 338–346, and 434–436 nm, together with an absorption shoulder at 220–224 nm as shown in Figure 3, but UV spectra of compound **10p** showed three absorption maxima at 276, 332, and 448 nm, together with an absorption shoulder at 222 and 248 nm and this change is due to the –I and + M effect of the OMe at 7 position of compound **10h**.

The ^1^H NMR spectra for 2-cyclohexyl substituted compounds (**10h**, **10j**, and **10l**) showed multiplet signals for the cyclohexyl protons and broad singlet signal at *δ*_H_ 4.21–5.0 ppm corresponding to exocyclic 2-NH, IR spectra showed stretching band at 3376–3398, 3229–3286, and 1695–1699 corresponding to the exocyclic 2-NH, 3-NH, and C═O groups, respectively.

The UV/vis absorption spectra of compounds (**10h**, **10j**, and **10l**) exhibited four absorption maxima at 222–224, 270–276, 330–338, and 436–452 nm. This bathochromic shift at 436–452 is due to the mesomeric effect of 2-NH group and the electron-donating effect of the cyclohexyl which increase the nucleophilicity of NH group and so the lone pair will be more available for conjugation resulting in a bathochromic shift.

The 2-amino-2-deoxoalloxazines (**14a,b**) were prepared according to the reported procedure^32^ by fusion of their precursors (**9d,c)** with ammonium acetate at 160–165 °C for 0.5–1.5 h to obtain brown needles of the corresponding product in 69, and 74% yields as illustrated in Scheme 3. Moreover, this nucleophilic replacement of the 2-CH_3_S group for compounds **9a–e** by the amino group was accompanied by deoxygenation of 2-deoxo-2-methylthioalloxaz-in-5-oxides to produce the 2-amino-2-deoxoalloxazine analogues (**14a,b**).

The IR spectrum of compound (**14a,b**) showed stretching bands at 3256–3269, 3140–3148, and 1698 cm^−1^ corresponding to exocyclic 2-NH, 3-NH, and C═O, respectively.

Acid hydrolysis of 2-methylthio-4-oxo-3,4-dihydrobenzo[*g*]pteridine-5-oxides (**9a–e**) was carried out according to the reported procedure^32^, by heating in 5N hydrochloric acid under reflux for 15 h to give 2,4-dioxo-1,2,3,4-tetrahydro-alloxazine-5-oxides. (**13a–d**) as yellow crystals in 77–78% yield as shown in Scheme 3.

Considering the ^1^H NMR spectra of 2-deoxo-alloxazine derivatives (**13a–d**), they are characterised by the disappearance of the strong characteristic singlet signals of the 2-methylthio groups, which were assigned for compounds **9a–e** at *δ*_H_ 2.62–2.63 ppm. And two characteristic D_2_O exchangeable broad singlet signals at *δ*_H_ 11.39–11.73 and 11.55–11.84 ppm belong to 1-NH and 3-NH, respectively. Aromatic protons of the fused benzene ring appeared at *δ*_H_ 7.51–8.32 ppm.

The IR spectra of compounds (**13a–d**) showed stretching bands at 3252–3186, 3107–3081, 1737–1699, and 1696–1620 cm^−1^, corresponding to the 1-NH, 3-NH, 4-C═O, and 2-C═O groups, respectively. The UV/vis absorption spectra of compounds (**13a–d**) exposed four absorption maxima at 214–216, 258–272, 318–334, and 380–424 nm, together with additional absorption shoulder at 240–242 for compound **13a**. These acid hydrolysed products exhibited hypochromic shift mainly in the long wavelengths in comparison with the corresponding spectra of 2-deoxo-2-thiomethylalloxazin-5-oxides (**9a–d**) due to loss of thiomethyl group at position 2 and the interruption of the conjugation system with the pyrimidin-2,4-dione ring.

The 2-deoxo-2-thiomethyl-alloxazine-5-oxides (**9a–c**) were reduced involving aqueous sodium dithionite Na_2_S_2_O_4_ solution, by stirring overnight at room temperature to afford 2-deoxo-2-thio-methyl alloxazines (**11a–c**) as yellow or orange needles in 65–67% yields, as shown in Scheme 3. The newly assigned structures were confirmed by mass analyses which reveal the disappearance of the oxygen atom at 5 positions in the molecular ion peak. The MS spectrum of compound **11a** showed a molecular ion peak C_11_H_8_N_4_OS at *m/z* 244 which is also the base peak (100%). The MS spectrum of compound **11b** showed a molecular ion peak C_12_H_10_N_4_OS at *m/z* 258 which is also the base peak (100%). The MS spectrum of compound **11c** showed a molecular ion peak of C_12_H_10_N_4_O_2_S at *m/z* 274 (73%) and a base peak of C_10_H_5_N_3_O_2_ at *m/z* 199 (100%).

The IR, ^1^H NMR, UV/vis spectra, elemental analysis, and mass analyses were used for elucidation of compounds (**11a–c**). Considering the ^1^H NMR spectra, these 2-deoxo-alloxazine derivatives are characterised by the presence of the strong characteristic singlet signal of the 2-CH_3_S group at *δ*_H_ 2.56–2.59 ppm, characteristic D_2_O exchangeable broad singlet signals at *δ*_H_ 12.77–12.80 ppm belong to 3-NH aromatic protons of fused benzene ring appeared at *δ*_H_ 7.38–8.14 ppm.

The IR spectra of compounds (**11a–c**) showed stretching bands at 3172–3175 and 1684–1687 cm^−1^ corresponding to the 3-NH and 4-C═O groups, respectively. The UV/vis absorption spectra of compounds (**11a–c**) showed three absorption maxima at 216–220, 348–352, and 438–456 nm, together with two additional absorption shoulders at 242–248 and 270–286 nm. Comparing these data with those of the corresponding 2-deoxo-2-thiomethyl-alloxazine-5-oxide (**9a–e**) which exhibited four absorption maxima at 272, 294–296, 360–364, and 442–448 nm, it has been revealed the loss of one absorption *λ* maxima at 294–296 nm and addition of one shoulder at 270–286 nm.

Interestingly, regioselective N^3^-alkylation of 2-deoxo-2-methyl-thioalloxazin-5-oxides (**9a–d**) was performed using excess alkyl iodide in the presence of anhydrous KCO_3_ in DMF at room temperature^19^ as shown in Scheme 3 to afford N^3^-alkyl-2-methyl-thioalloxazin-5-oxides (**12a–e**) as yellow crystals in (66–76)% yield. This alkylation is 100% regiospecific at N^3^-position, offering a highly pure product.

Regarding the ^1^H NMR spectra of the N^3^-alkyl-2-methylthio-alloxazin-5-oxides (**12a–e**), they are characterised by the absence of the characteristic broad singlet signal of the 3-NH group which was attributed for compounds **9a–e** at *δ*_H_ 12.80 ppm and the appearance of singlet signal peak for 3-NCH_3_ at *δ*_H_ 3.44–3.46 ppm for compounds (**12b,c**). And for 3-ethyl-2-deoxo-2-methylthioalloxazin-5-oxide analogues (**12a**, **12d**, and **12e**), they exhibited a characteristic triplet peak at *δ*_H_ 1.27–1.28 for 3-NCH_2_-*CH_3_* and a quartette peak at *δ*_H_ 4.03–4.07 ppm for 3-N*CH_2_*-CH_3_, with the presence of the strong characteristic singlet signal of the 2-CH_3_S group at *δ*_H_ 2.64–2.70 ppm, and the aromatic protons of the fused benzene ring appeared at *δ*_H_ 7.60–8.36 ppm.

The IR spectra of **12a–e** showed the presence of a C═O band at 1683–1697cm^−1^ with the absence of a 3-NH stretching band. The UV/vis absorption spectra of compounds (**12a–e**) exhibited four absorption maxima at 216–222, 272–280, 292–304, and 402–426 nm, together with two additional absorption shoulders at 336–346 and 436–454 nm as. The UV/vis spectrum of 7-methoxy-containing derivative (**12b)** has identical absorption maxima with loss of 2nd maximum at 272–280 nm.

Interestingly and for the first time in flavin chemistry, the 2-unsubstituted 2-deoxo-alloxazine derivatives were obtained by the dethiation approach of the corresponding 2-meththio analogues. The required 6-(*N*-anilino)pyrimidin-4(3*H*)-ones (**15a–c**), which were used as precursors for synthesis of 4-oxo-3,4-dihydro-alloxazine-5-oxides (**16a–c**), were synthesised by dethiation of 6-(N-anilino)-2-methylthio-3,4-dihydropyrimidine-4-ones (**8a,d,c**) according to the reported procedure^19^ by using Raney nickel by heating in ethanol under reflux for 0.5–1.5 h to afford 58–72% yields (Scheme 4).

Regarding the ^1^H NMR spectra, the structure of 6-(*N*-anilino)pyrimidin-4(3*H*)-ones (**15a–c**) was confirmed by the presence of a singlet signal at *δ*_H_ 4.83–5.20 corresponding to an equivalent proton resonance at the 5-position with the disappearance of the strong characteristic singlet signal of the 2-CH_3_S group which was attributed for the starting compounds, namely, 6-(N-anilino)-2-methylthio-3,4-dihydropyrimidine-4-ones (**8a,d,c**) at *δ*_H_ 2.43–2.52 ppm, and a characteristic appearance of an equivalent proton resonance at the C2-position as a singlet signal at *δ*_H_ 7.80–7.90.

The IR spectra of compounds (**15a–c**) revealed stretching bands at 3407–3302, 3220–3210, and 1671–1637 cm^−1^ corresponding to the 6-NH, 3-NH, and 4-C═O groups, respectively. The UV/vis absorption spectra of compounds (**15a–c**) showed two absorption *λ* maximum at 242–250 and 276–288 nm, in comparison with compounds (**8a,d,c**), they exhibited hypochromic shift due to loss of thiomethyl group which caused red shift as mentioned before.

The dethiated alloxazine analogues, namely, 4-oxo-3,4-dihydrobenzo[*g*]pteridin-5-oxides (**16a–c**), were synthesised by nitrosative cyclisation of 6-(*N*-anilino) pyrimidin-4(3*H*)-ones (**15a–c**) using 2–4 equiv. of sodium nitrite in acetic acid, initiated at 10–15 °C, then at room temperature for 4–8 h. Meanwhile, the formed greenish-yellow nitroso intermediate should be completely dissolved in the acetous reaction mixture by occasional warming in a boiling water bath. This warming enhances the completed cyclisation of nitroso intermediate to afford the final cyclised product. Such a step of reaction termination is needed, where the nitroso intermediate was detected as impurities in ^1^H NMR spectra. The completed cyclisation of 5-nitroso-6-(*N*-anilino) pyrimidinone intermediate into the final benzo[*g*]pteridin-5-oxide product can be easily detected by conversion of greenish-yellow colour into the yellow or yellowish brown product, with 70–77% yields.

Considering ^1^H NMR, the structures of the compounds (**16a–c**) were confirmed by the disappearance of the 5-position singlet and the presence of an equivalent proton resonance at the 2-position as a singlet signal at *δ*_H_ 8.21–8.26 and D_2_O exchangeable broad singlet signal at *δ*_H_ 12.47–12.51 ppm corresponding to the 3-NH group.

The IR spectra of compounds (**15c** and **16b**) showed stretching bands at 3250–3133 and 1705–1700 cm^−1^ corresponding to the 3-NH and 4-C═O groups, respectively (Figure S3).

The UV/vis absorption spectra of the products (**16a**, **16b**, and **16c**) exhibited four absorption maxima at 216–266, 230–290, 282–340, and 380–430 nm. They exhibited a hypsochromic shift in comparison with their corresponding 2-methylthio-analogues compound (**9a–e)**. This is assigned to the loss of the S-atom which induces a red shift (bathochromic shift) in the spectrum due to its facial polarisability^33^.

### *In vitro* antitumor activities of alloxazin derivatives against human tumour cell lines

The MTT assay developed by Mosmann^34^ was modified^35^ and used to determine the inhibitory effects of test compounds on cell growth *in vitro* as mentioned in the experimental part in detail. Twenty-nine out of 45 newly compounds were investigated *in vitro* for their growth inhibitory activities, namely, (**9d,e**), (**10a–q**), (**12a–e**), (**14a,b**), and (**16a–c**). Two human tumour cell lines, namely, the human T-cell acute lymphoblastoid leukaemia cell line (CCRF-HSB-2**)** and human oral epidermoid carcinoma cell line (KB) were used, and the antitumor agent “Ara-C” was used as a positive reference in this investigation.

Among the tested compounds, several, including **9e**, **10h**, **10j**, **12a**, **12d**, **16a**, and **16b**, demonstrated significant antitumor activity against both CCRF-HSB-2 and KB cell lines. These compounds exhibited IC_50_ values ranging from 0.87 to 6.53 µg/mL for CCRF-HSB-2 and 0.47 to 7.71 µg/mL for KB, indicating their potency (Table 1). However, despite their effectiveness, these compounds were still less potent than Ara-C, which showed exceptional inhibitory activity with IC_50_ values of 0.059 µg/mL and 0.091 µg/mL for CCRF-HSB-2 and KB, respectively.

**Table 1.** Growth inhibitory activities of target compounds against CCRF-HSB-2 and KB tumour cell lines.

Compound	Inhibitory activity (IC_50_ (µg/mL))			Compound	Inhibitory activity (IC_50_ (µg/mL))	
	CCRF-HSB-2		KB		CCRF-HSB-2	KB
**9d**	11.20		11.40	**10n**	>100	2.72
**9e**	0.87		0.47	**10o**	65.90	50.10
**10a**	>100		26.10	**10p**	11.0	11.50
**10b**	55.90		56.50	**10q**	73.2	54.40
**10c**	>100		56.90	**12a**	5.86	6.11
**10d**	68.0		43.30	**12b**	51.80	8.83
**10e**	37.90		29.20	**12c**	7.70	8.10
**10f**	>100		37.30	**12d**	6.53	6.45
**10g**	10.10		11.10	**12e**	8.34	8.36
**10h**	5.66		2.57	**14a**	10.20	4.11
**10i**	11.50		19.40	**14b**	18.0	2.89
**10j**	4.17		2.13	**16a**	3.36	6.91
**10k**	19.60		7.27	**16b**	3.13	7.71
**10l**	21.50		18.0	**16c**	8.88	8.55
**10m**	12.10	9.93		**AraC**	0.059	0.091

In addition, compounds such as **9d**, **10g**, **10m**, **10p**, **12c**, **12e**, and **16c** displayed reasonable growth inhibitory activities with IC_50_ values in the range of 7.7–12.1 µg/mL for CCRF-HSB-2 and 8.1–11.5 µg/mL for KB. While not as potent as the most active compounds, these still exhibited noteworthy antitumor effects. Furthermore, compound **12b** showed selective antiproliferative activity against KB cells with an IC_50_ of 8.83 µg/mL, compared to a much higher IC_50_ of 51.8 µg/mL against CCRF-HSB-2, indicating a preferential cytotoxic effect. Similarly, compound **10i** exhibited better activity against CCRF-HSB-2 (IC_50_ = 11.50 µg/mL) than KB (IC_50_ = 19.4 µg/mL), highlighting differential activity profiles among the compounds.

Compound (**12b**) showed selective antiproliferative activities against KB tumour cell lines over (CCRF-HSB-2) tumour cell lines. Its IC_50_ against KB tumour cell lines was 8.83 µg/mL, whereas its IC_50_ against (CCRF-HSB-2) tumour cell lines was 51.8 µg/mL, indicating a preference in its cytotoxic effect.

Compound **10i** showed better activity against CCRF-HSB-2 (IC_50_ = 11.50 µg/mL) compared to KB (IC_50_ = 19.4 µg/mL).

The extent of the activities for the compounds (**10b,d,e,o,q**) were the lowest among their analogues against both of human (CCRF-HSB-2) and (KB) cell lines, being of IC_50_ in the range of 37.9–73.2 µg/mL and 29.2–56.5 µg/mL, respectively. These compounds are significantly less potent than Ara-C and the other more active compounds in the series.

The novel alloxazin derivatives, particularly **16a** and **16b** with the unsubstituted alloxazin-5-oxide skeleton or substituted by the 9-Me group, also showed promising growth inhibitory activities, with IC_50_ values of 3.13–3.36 µg/mL for CCRF-HSB-2 and 6.91–7.71 µg/mL for KB, demonstrating their potential as effective antitumor agents. Overall, while the newly synthesised compounds show varying degrees of potency, with some exhibiting significant antitumor activities, they generally fall short when compared to the high potency of Ara-C, suggesting that further structural optimisation may be necessary to enhance their efficacy.

### Protein kinase profiling

The protein kinase profiling was carried out following the standardised procedure of KINEXUS Corp. (Vancouver, Canada), for the most potent compounds. This screening includes compound (**9e**) and (**10j**), against a panel of 20 protein kinases. Results, presented in Table 2, show the percentage change in activity and inhibition compared to the control. Intra-assay variability was below 10%. Negative (−) values indicate inhibition, while positive (+) values indicate activation. The activation/inhibition values above 25% are considered significant.

**Table 2.** % Activity or inhibition of 20 kinases in the presence of compounds **9e**, **10j**, and reference compound (**Imatinib**) using the radiometric assay method.

Kinase	9e	10j	Imatinib
ABL1	6%	6%	−81%
ACVR2A	−1%	−1%	−1%
BRAF	−1%	−4%	−25%
CAMK1	**−17%**	−12%	−20%
CAMK2 alpha	6%	6%	7%
CDK1/cyclin A1	19%	12%	8%
CK1 alpha	4%	−1%	11%
c-KIT	3%	−1%	−72%
EGFR	**−14%**	**−16%**	5%
ERK1	5%	2%	1%
FAK	−2%	4%	−11%
FGFR1 (FLT2)	0%	3%	1%
VEGRF1	−4%	2%	−29%
FLT3	**−19%**	39%	17%
HER2	2%	10%	−2%
JNK1	−4%	−3%	−21%
MET	−1%	1%	3%
PCTK1 (CDK16) cyclin	−1%	4%	−3%
PKC beta II	−1%	1%	−1%
SRC	−4%	−19%	−2%

A negative (−) value indicates inhibition. A positive (+) value indicates activation.

The bold negative values indicated the most inhibitory values. Values >25% in both activity or inhibitions are considered significant.

Using the radiometric assay method, profiling data for compounds **9e** and **10j** against the kinase panel revealed inhibitions and activations ranging from 1% to 19% for inhibitions and 1% to 39% for activations. Notably, compounds **9e** and **10j** exhibited low to moderate inhibition (10–31%) against select kinases, including CAMK1, EGFR, FLT3, SRC, and others. Only six out of the 20 tested kinases, ACVR2A, CAMK1, EGFR, FAK, FLT3, and SRC, showed varying levels of inhibition at 10 µM concentrations.

Among these, CAMK1 was inhibited by all compounds (10–17% inhibition). Compound **10j** showed a 19% inhibition of SRC kinase, and FLT3 was inhibited by compound **9e** by 19%. Imatinib, a reference compound, exhibited high inhibitions with ABL1 (81%) and c-KIT (72%) kinases.

Additionally, some kinases demonstrated moderate activations (11–39%) with compounds **9e**, **10j**, and Imatinib. Only three out of the 20 kinases, CDK1/cyclin A1, FLT3, and SRC, showed varying activation levels at 10 µM concentration, with CDK1/cyclin A1 being activated by all compounds (9–19% activation). Compound **10j** showed the highest activation of 39% with FLT3. Imatinib also exhibited slight activations with CK1 alpha (11%) and FLT3 (17%). Other measurements within the ±10% range were considered insignificant.

#### ADME-T and drug likeness predictions of target compounds

The physicochemical properties, toxicity, and pharmacokinetic (PK) properties of the most potent compounds were conducted using the Swiss ADME^36^ online web tool accessible through the Swiss Institute of Bioinformatics (SIB). This ADME tool was utilised for the calculation of the key PK properties for drug-likeness of the promising candidates **9e**, **10h**, **10j**, **10n**, **12a**, **16a**, and **16b**. The predicted PK, drug-likeness criteria, and toxicity of the most active alloxazine derivatives suggest that the compounds have exhibited promising PK properties that align with the drug-likeness criteria set by major pharmaceutical companies. These criteria, which include Ghose’s criteria developed by Amgen, Lipinski’s rule by Pfizer, Muegge’s criteria by Bayer, Veber’s criteria by GSK, and Egan’s criteria by Pharmacia, are often used to assess the drug-likeness of compounds. These criteria consider various physicochemical properties and molecular features that are desirable for a compound’s success in drug development.

**Figure 4.**
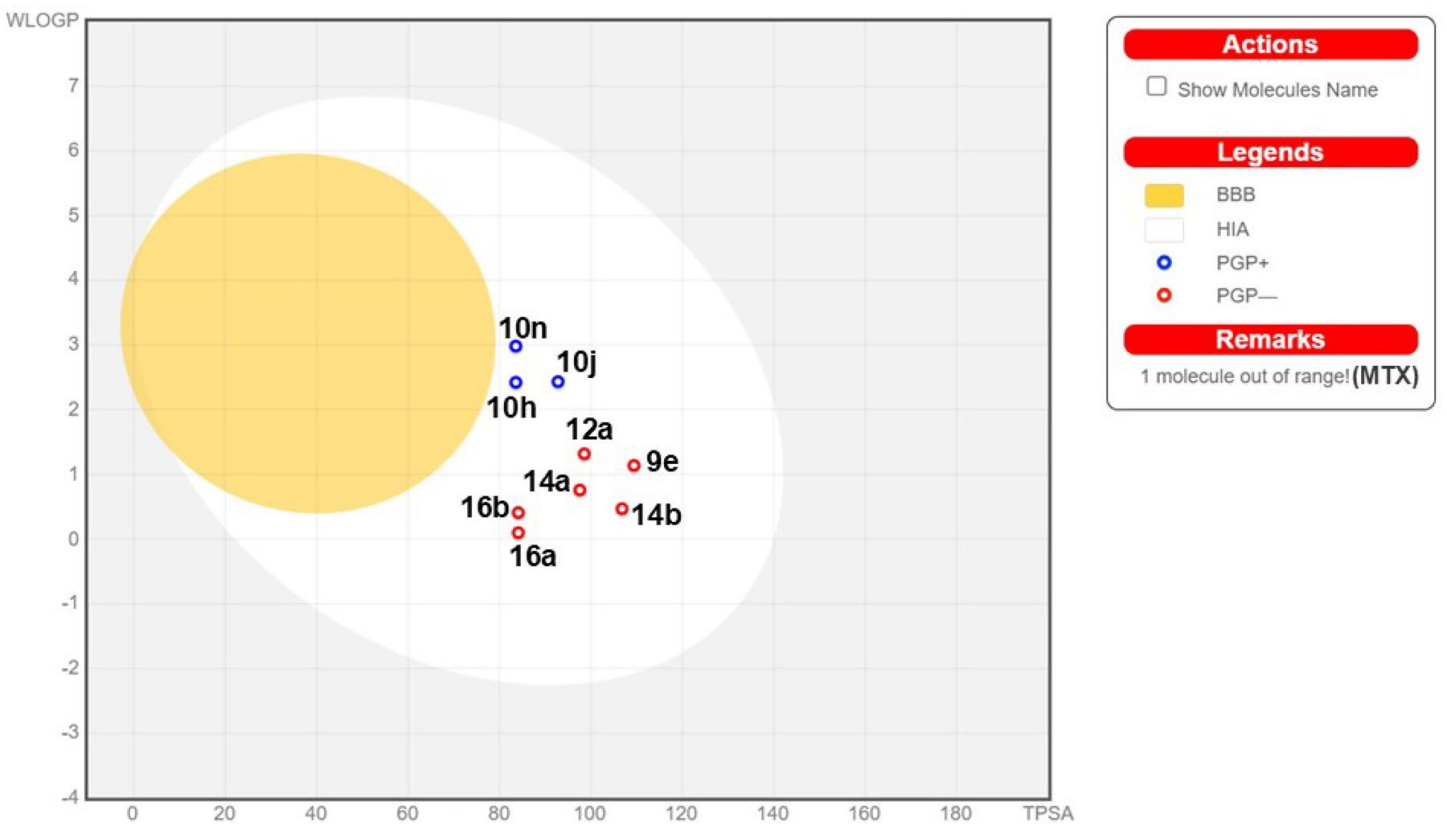
Predicted BOILED-Egg plot from SwissADME online web tool for compounds **9e**, **10h**, **10j**, **10n**, **14a**, **14b**, **12a**, **16a**, and **16b** compared to methotrexate (MTX).

Figure 4 depicts the BOILED-Egg graph for the relationship between two specific physicochemical properties: WLOGP (octanol/water partition coefficient) and TPSA (topological polar surface area) compared with methotrexate (MTX). The synthesised compounds fall within the range associated with human intestinal absorption (HIA), indicating that they are likely to be effectively absorbed in the gastrointestinal tract (GIT). This optimal absorption is attributed to the compounds’ physicochemical properties, which are within the acceptable range for oral bioavailability.

Compounds **10n** and **10h** are predicted to be the closest compounds to cross the blood–brain barrier (BBB), while compounds **9e**, **14a,b**, and **16a,b** are not expected to pass through the BBB owing to their high polarity caused by the presence of N-oxides (**9e** and **16a,b**) and 2-amino groups (**14a,b**).

To further evaluate the potential toxicity of the compounds, toxicity prediction was performed using ProTox-II software^37^ (https://tox-new.charite.de/protox_II/index.php?site=compound_input) and Swiss ADME^36^ (http://www.swissadme.ch/). The results confirmed the above findings that the 2-(substituted amino)benzo[*g*] pteridin-4(3*H*)-one derivatives “2-substituted amino alloxazines” (**10a–j**) have a toxicity class of 4 and LD50 of 825–1500 mg/kg, indicating a favourable safety profile compared to MTX which is classified as class 1, indicating high toxicity, although further studies may be required to validate these calculations (Table 3). However, 2-amino-alloxazine (**14b**) and 4-oxo-3,4-dihydro-alloxazine-5-oxide derivative (**16a**), with toxicity class of 3 and predicted LD50 of 200–300 mg/kg are anticipated to have some hepatotoxicity (Table 3).

**Table 3.** The predicted toxicity using toxicity model computation and SWISS ADME calculation for the most potent compounds (**4e**, **4j**, **4k**, **5d**, and **9f**).

Compd	Predicted LD50: mg/kg	Predicted toxicity class	Prediction accuracy %	Average similarity %	Hepato-toxicity	PAINS^a^	Brenk alerts^b^	Lead likeness^c^	GI absorption	BBB permeant
**9e**	1000	4	67.38	50.91	Active	Pyridinium	N_oxide, polycyclic aromatic HC (2), quaternary nitrogen (1)	Yes	High	No
**10h**	1500	4	67.38	51.91	Inactive	0	Polycyclic aromatic HC (2)	Yes	High	No
**10j**	825	4	67.38	51.14	Inactive	0	Polycyclic aromatic HC (2)	Yes	High	No
**10n**	1500	4	67.38	54.28	Inactive	0	Polycyclic aromatic HC (2)	Yes	High	No
**12a**	1000	4	67.38	52.82	Inactive	Pyridinium	N_oxide, polycyclic aromatic HC (2), quaternary nitrogen (1)	Yes	High	No
**14a**	1200	4	67.38	51.71	Active	0	Polycyclic aromatic HC (2)	MW < 250	High	No
**14b**	300	3	67.38	51.25	Active	0	Polycyclic aromatic HC (2)	MW < 250	High	No
**16a**	200	3	67.38	50.25	Active	Pyridinium	N_oxide, polycyclic aromatic HC (2), quaternary nitrogen (1)	MW < 250	High	No
**16b**	3000	5	67.38	50.74	Active	Pyridinium	N_oxide, polycyclic aromatic HC (2), quaternary nitrogen (1)	MW < 250	High	No
**MTX** ^d^	3.0	1	100	100	Active	0	0	MW >350 Rotors >7	Low	No

^a^ PAINS: (pan-assay interference compounds) are a prominent source of false positives in the drug-discovery process^38^.

^b^ Brenk is a filter to identify compounds that have the accepted toxic level, chemical reactivity, and metabolically unstable^39^.

^c^ Leadlikeness: 250 ≤ MW ≤ 350; *X*log*P* ≤ 3.5; rotatable bonds ≤7.

^d^ MTX: methotrexate.

### Molecular modelling study

#### Rationale for selecting target kinases

The selection of Abl (PDB: 2hyy), c-kit (PDB: 1t46), FAK (PDB: 4q9s), Src (PDB: 4mxo), B-raf (PDB: 4rzv), and VEGFR1 (PDB: 3hng) kinases for screening was done based on significant preliminary inhibition data in our previous reports^11^^,^^21^ and this study as well. These results are comparable to the inhibition observed with Imatinib, a well-known tyrosine kinase inhibitor. The selected kinases are critical in cancer-related pathways, making them important targets for therapeutic research. The validation of the screening process through reliable docking studies further supports the relevance of these findings, indicating the potential of our compounds as a promising kinase inhibitor.

### GOLD molecular docking

The accuracy of the GOLD 5.2.2 docking program was validated to ensure the reproducibility by redocking the co-crystallised ligands into their corresponding six kinases, which include: Imatinib (STI571, Gleevec) (PDB: 2hyy)^40^; Vemurafenib (PDB: 4rzv)^41^; TI-571 c-kit kinase (PDB: 1t46)^42^; 1 (3,5-dihydro[1,2,4]triazino[3,4-c][1,4]benzoxazin-2(1*H*)-one) (PDB: 4q9s)^43^; N-(4-chlorophenyl)-2-((pyridin-4-ylmethyl)amino)benzamide (PDB: 3hng)^44^; and Bosutinib (PDB: 4mxo)^45^ as we recently described in detail^21^.

The docking results of the synthesised alloxazine and alloxazine-N-oxides into ABL, C-Kit, B-Raf, FAK, VEGFR1, and SRC kinases are reported in Table 4 compared to the corresponding co-crystallised ligands. As depicted in Table 4, the highest-scored and best-docked compound are **10q** into Abl kinase; compound **10n** into B-Raf kinase, compound **10q** into C-Kit kinase, compound **10j** into FAK kinase, compound **10q** into VEGFR1 kinase, and compounds **10j** into SRC kinase.

**Table 4.** Docking results for the synthesised alloxazine and alloxazine-5-oxide analogues docked into six different kinases compared to the co-crystallised ligand using the GOLD molecular docking program.

Compound	Abl (PDB: 2hyy)	B-RAF (PDB: 4rzv)	C-Kit (PDB: 1t46)	FAK (PDB: 4q9s)	VEGFR1 (PDB: 3hng)	SRC (PDB: 4mxo)
**5a**	51.8	50.6	51.7	39.1	48.5	52.0
**5b**	54.7	53.3	56.1	38.2	52.6	52.7
**9a**	56.0	56.4	50.5	53.0	54.5	52.4
**9b**	**63.6**	57.1	51.4	44.3	56.5	55.7
**9c**	58.6	59.3	58.0	48.0	56.9	**58.8**
**9d**	56.6	**60.2**	52.5	54.2	55.0	55.0
**9e**	**62.6**	**60.5**	52.8	51.2	55.4	51.6
**10a**	55.8	53.6	53.4	50.0	57.6	49.3
**10b**	57.0	58.5	57.1	**55.5**	59.9	46.0
**10c**	**63.8**	**64.4**	**63.9**	**57.4**	**64.4**	**59.9**
**10d**	58.8	55.6	54.3	52.9	62.1	48.8
**10e**	60.1	57.6	59.4	46.1	56.5	48.2
**10f**	60.0	53.1	58.0	48.7	56.0	47.9
**10g**	58.8	55.6	57.7	44.6	56.6	50.6
**10h**	59.6	53.5	58.7	51.5	58.5	54.4
**10i**	**64.7**	**62.0**	58.2	51.5	**64.0**	**56.6**
**10j**	57.3	**63.7**	**63.5**	**58.9**	57.5	**64.1**
**10k**	61.9	**61.4**	58.2	46.9	57.9	51.5
**10l**	60.0	57.1	**61.6**	52.2	57.0	53.7
**10m**	61.5	**62.9**	56.5	52.5	57.7	50.1
**10n**	**65.3**	**65.4**	**60.9**	**57.9**	**66.8**	**59.8**
**10o**	**62.7**	**60.8**	**60.7**	48.3	**70.0**	**57.4**
**10p**	61.5	**60.6**	**63.9**	52.3	**66.6**	**60.0**
**10q**	**66.1**	53.5	**67.1**	50.2	**76.3**	52.0
**12a**	53.6	57.8	52.2	54.0	58.4	49.8
**12b**	58.9	**62.5**	54.0	47.0	59.4	**56.0**
**12c**	**63.5**	**61.2**	54.1	**55.4**	56.9	45.3
**12d**	59.5	60.6	54.4	45.6	62.3	49.5
**12e**	57.5	59.4	52.5	**55.6**	56.6	45.4
**13a**	49.7	52.9	44.7	49.4	49.8	49.1
**13b**	53.1	57.3	54.5	42.3	54.1	**56.2**
**13c**	52.1	54.1	47.5	43.0	53.3	45.8
**13d**	51.7	55.6	48.1	37.7	51.3	51.5
**14a**	56.0	52.0	51.9	41.4	53.1	48.2
**14b**	55.2	58.9	47.5	45.3	53.8	49.9
**16a**	55.0	49.1	49.7	36.1	50.0	51.4
**16b**	59.1	49.7	50.4	48.6	53.0	53.0
**16c**	58.0	54.3	51.7	43.4	52.8	52.7
Native ligand^a–f^	**104.2** ^a^	**94.8** b	**76.0** c	**41.7** d	**86.4** e	**77.2** f

^a^ Imatinib (STI571, Gleevec) (PDB: 2hyy)^40^.

^b^ Vemurafenib (PDB: 4rzv)^41^.

^c^ TI-571 c-kit kinase (PDB: 1t46)^42^.

^d^ 1 (3,5-dihydro[1,2,4]triazino[3,4-c][1,4]benzoxazin-2(1H)-one) (PDB: 4q9s)^43^.

^e^ N-(4-Chlorophenyl)-2-((pyridin-4-ylmethyl)amino)benzamide (PDB: 3hng)^44^.

^f^ Bosutinib (PDB: 4mxo)^45^.

The highest scoring values (>55) are indicated in bold and considered as significant.

**Figure 5.**
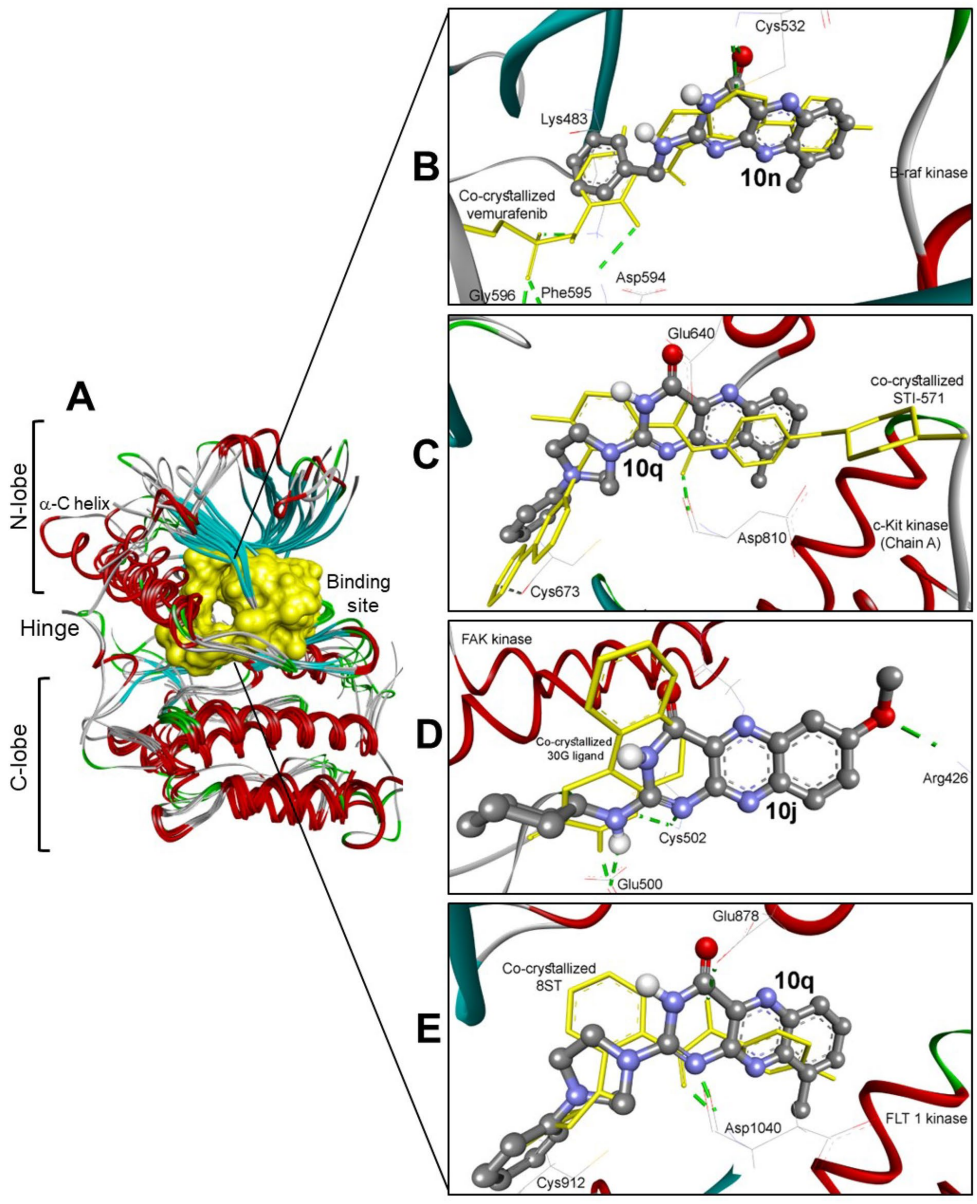
(A) The molecular alignment of six kinases including Abl (PDB: 2hyy), c-kit (PDB: 1t46), FAK (PDB: 4q9s), Src (PDB: 4mxo), B-raf (PDB: 4rzv), and **VEGFR1** (PDB: 3hng) with identical ATP binding pocket in yellow surface view. (B) The binding mode of compound **10n** into B-raf kinase (PDB: 4rzv) shows one hydrogen bond with Cys532. (C) The docking mode of compound **10q** into C-Kit kinase chain A (PDB: 1t46) interacting hydrophobically within RMSD of 1.27 Å from the co-crystallised STI-571 ligand. (D) The binding poses of compound **10j** into FAK kinase (PDB: 4q9s) showing three hydrogen bonds with Arg426, Glu 500, and Cys502 identical to the co-crystallised 30 G ligand. (E) The docking of compound **10q** into **VEGFR1** kinase (PDB: 3hng) revealing one hydrogen bond with the key amino acid ASP1040.

The best docked compounds into these six kinases are **10j**, **10n**, and **10q** (Figure 5). These results are correlated to some extent to the MTT antiproliferative assay, particularly for compounds **10j** and **10n**.

### AutoDock molecular docking study

Molecular docking is an extensively used tool in computer-aided structure-based rational drug design, assessing how small molecules (ligands: substrate, inhibitor, etc.) and target macromolecules (receptors: receptor, enzyme, etc.) fit together. AutoDock Tools (ADT), available from http://autodock.scripps.edu/, automates docking to predict ligand binding to known 3D-structure target proteins. Besides generating binding energies, it visualises ligand positions in the host’s binding site, aiding drug candidate development and understanding of binding nature.

Protein tyrosine kinases are crucial in cancer and targeted for therapeutic intervention. Receptor and non-receptor PTK inhibitors show promise as antitumor agents, impacting different cancer cell features including proliferation, survival, invasion, and angiogenesis. Investigating AutoDock binding affinities for synthesised flavin analogues (**5a,b**, **7d,e**, **8d,e**, **9d,e**, **10a–q**, **11a–c**, **12a–e**, **13a–d**, **14a,b**, **15a–c**, and **16a–c**), a computer-simulated automated docking study optimises lead compounds for antitumor activity. Autodock 4.2 facilitates flexible ligand–protein docking, utilising a genetic algorithm with a local search (GALS) and precomputed grids for interaction energy evaluation.

This study utilised the advanced and commonly used molecular grid-based docking software programs^46–48^. Specifically, Autodock 4.2^49^ was employed for docking a flexible ligand within a partially flexible protein. Autodock explores the active site to identify low-energy binding models and orientations of the probe molecule. It employs precomputed grids and a modified GALS to evaluate interaction energy for low-energy binding models and probe molecule orientations.

The target PTK enzyme (PDB code 1t46), was isolated using Accelrys Discovery Studio 3.5 software, developed by Accelrys Inc. (San Diego, CA) (2005). Within the 5 Å vicinity surrounding the embedded native ligand, STI-571 (Imatinib or Gleevec), the key amino acids of the ligand-binding site were meticulously selected. Subsequently, the results obtained from 10 randomly seeded runs for each of the docked inhibitors underwent thorough analysis. In the process, the docked inhibitors were categorised into clusters if the atomic coordinates displayed a root mean square deviation (RMSD) of ≤0.5 Å. These clusters were then ranked spanning from the lowest to the highest energy. The comprehensive analysis focused on the top 10 docking clusters. Then, each cluster revealing significant negative binding interaction energies (Δ*G*_b_) was investigated by Accelrys Discovery Studio visualise.

### AutoDock binding affinities of the synthesised and designed compounds into PTK

The binding free energies (Δ*G*_b_, kcal/mol) and inhibition constants (*K_i_*) were used to evaluate the binding affinity in addition to hydrogen bonding RMSD values. The compounds which exhibited the highest binding affinities within PTK are represented in Table 5. These compounds include 2-deoxo-2-thiomethyl-alloxazin-5-oxides (**9d,e**), 2-(substituted amino)benzo[*g*]pteridin-4(3*H*)-ones (**10a,b,d,e,g–i,m–o**), 2-methylthiobenzo[*g*]pt-eridin-4(3*H*)-ones (**11a,b**), and N^3^-alkyl-2-methylthio-alloxazin-5-oxides (**12a,c–e**).

**Table 5.** The flexible docking results (AutoDock 4.2) of compounds docked into PTK, including the binding free energies (Δ*G*_b_, kcal/mol), inhibition constants (*K_i_*), distances (Å), and angles of hydrogen bonds between compounds and amino acids involved in PTK, and RMSD from co-crystallised STI-571 ligand.

	Δ*G*_b_^a^ (kcal/mol)		Hydrogen bonds between atoms of compounds and amino acids of PTK				
Comp.		*K_i_* ^b^	Atom of comp.	Amino acid	Distance (Å)	Angle (°)	RMSD^c^ (Å)
**5a**	−11.10	7.31 nM	N^3^	NH of Cys673	1.76	155.2	6.38
**5b**	−10.28	29.05 nM	2NHa 2NHb	O═C of Ile789 O═C of His790	2.27 2.15	120.7 161.1	4.94
**7d**	−10.53	19.08 nM	4 C═O	NH of Cys673	2.00	155.4	6.07
**7e**	−9.40	129.24 nM	N^1^	O═C of Asp810	2.18	151.3	7.10
**8d**	−10.79	12.27 nM	6NH	O═C of His790	1.98	116.7	9.70
**8e**	−10.08	40.59 nM	6NH	O═C of His790	2.26	122.9	7.83
**9d**	−12.35	882.03 pM	5 N–O 4 C═O	NH of Cys673 HO of Thr 670	2.03 1.92	131.9 123.1	6.32
**9e**	−12.11	1.32 nM	5 N–O 4 C═O	NH of Cys673 NH of Cys673	2.05 2.15	151.2 118.7	7.44
**10a**	−12.31	955.22 pM	N^3^H	NH of Thr670	1.86	156.0	5.83
**10b**	−12.94	328.83 pM	N^3^H	NH of Thr670	1.88	149.9	5.52
**10c**	−11.08	7.60 nM	N^1^ N^10^ 2NH	HN of Asp810 HN of Asp810 O═C of Glu640	2.12 2.24 2.10	133.7 161.0 100.9	0.87
**10d**	−12.74	458.93 pM	N^1^ N^10^	HN of Asp810 HN of Asp810	1.92 2.26	129.6 160.3	0.83
**10e**	−14.00	54.98 pM	4 C═O	NH of Cys673	2.11	148.1	6.35
**10f**	−11.28	5.39 nM	4 C═O	NH of Cys673	2.00	145.5	6.69
**10g**	−12.30	965.45 pM	N^3^H	O═C of Asp810	2.06	151.1	2.51
**10h**	−12.91	346.76 pM	4 C═O N^10^	HN of Lys623 HN of Asp810	1.84 2.05	146.9 136.8	0.12
**10i**	−12.44	764.37 pM	4 C═O	NH of Cys673	2.18	132.5	7.22
**10j**	−9.23	170.69 nM	N^3^H	O═C of Ile789	2.26	161.4	7.33
**10k**	−11.31	5.11 nM	N^3^H	O═C of His790	2.14	123.6	7.78
**10l**	−11.88	1.97 nM	N^10^	HN of Asp810	2.19	150.4	1.88
**10m**	−14.44	25.88 pM	4 C═O	HN of Asp677	2.23	174.9	10.28
**10n**	−12.79	422.27 pM	4 C═O	HN of Cys673	1.78	147.6	7.57
**10o**	−12.47	729.34 pM	4 C═O	HN of Cys673	1.98	140.5	7.54
**10p**	−10.55	18.59 nM	2NH	OOC of Asp810	1.82	105.8	9.73
**10q**	−10.73	13.55 nM	2NH N^3^H	O═C of His790 O═C of His790	2.23 1.78	134.9 146.3	7.89
**11a**	−12.76	446.32 pM	N^10^	HO of Thr 670	2.46	100.9	4.02
**11b**	−12.42	786.40 pM	N^10^	HN of Asp810	2.01	135.4	1.03
**11c**	−11.60	3.16 nM	N^10^	HN of Asp810	2.39	136.9	1.11
**12a**	−12.40	811.38 pM	^*d*^				7.46
**12b**	−11.48	3.84 nM	5 N–O	HN of Cys673	1.92	166.2	8.82
**12c**	−12.80	415.63 pM	5 N–O 4 C═O	HN of Cys673 HO of Thr670	2.37 1.95	130.4 128.0	6.58
**12d**	−12.75	454.69 pM	5 N–O	HO of Thr670	1.98	138.7	5.48
**12e**	−12.76	442.22 pM	4 C═O	HN of Cys673	2.50	148.2	5.62
**13a**	−11.41	4.33 nM	2 C═O N^3^H	HN of Asp810 O═C of Asp810	2.12 1.91	153.3 143.5	3.88
**13b**	−11.70	2.67 nM	4 C═O 5 N–O	HN of Cys673 HO of Thr670	2.01 1.99	127.2 131.2	5.49
**13c**	−12.01	1.57 nM	4 C═O 5 N–O	HN of Cys673 HO of Thr670	2.02 1.98	135.3 135.6	5.72
**13d**	−12.00	1.60 nM	4 C═O 5 N–O	HN of Lys623 HO of Thr670	2.14 1.92	141.0 132.3	4.98
**14a**	−11.67	2.80 nM	4 C═O	HN of Asp677	2.13	152.9	9.95
**14b**	−10.68	14.73 nM	4 C═O	HN of Asp810	1.85	159.2	3.51
**15a**	−10.23	31.52 nM	6NH	OH of Thr670	1.92	142.4	3.44
**15b**	−10.08	40.99 nM	4 C═O	HN of Lys623	2.33	146.1	5.42
**15c**	−10.36	25.63 nM	N^3^H 6NH	O═C of Glu640 OH of Thr670	2.17 2.00	140.1 162.4	3.61
**16a**	−11.07	7.67 nM	4 C═O N^3^H	HN of Lys623 O═C of Asp810	2.07 2.30	134.4 127.9	3.64
**16b**	−11.63	2.97 nM	4 C═O 5 N–O	HO of Thr670 HN of Cys673	1.94 1.80	133.7 143.1	7.20
**16c**	−11.70	2.65 nM	4 C═O 5 N–O	HN of Cys673 HO of Thr670	1.95 2.04	138.7 135.1	5.11
**STI** ^e^	−12.55	627.24 pM	C═O (amide) NH (amide)	HN of Asp810 O═C of Glu640	2.26 2.35	131.9 107.1	0.25

^a^Binding free energy.

^b^Inhibition constant.

^c^Root means square deviation.

^d^No hydrogen bond detected.

^e^The native co-crystallised bound ligand (STI-571) of PTK (PDB code: 1t46).

The main groups of the docked flavin analogues involved in hydrogen binding within the PTK receptor site are: 4C═O, 5N–O, 2NH, 6NH, N^3^H, N^10^, and to the lower extent N^1^ atom. The main amino acids of the PTK receptor involved are C═O, NH of Asp810, C═O of His790, NH of Cys673, OH of Thr670, C═O of Glu640, C═O of Ile789, and to a lower extent NH of Asp677, and NH of Lys623. The formed hydrogen bonds are established within RMSD of 0.12–10.28 Å, of a more abundant range of 3.0–7.0 Å as cited in Table 5. One to three hydrogen bonds were exhibited between the docked inhibitors and PTK. Where compound **10c** revealed the three bonds, while compounds **5b**, **9e**, **10d,h,q**, **13a–d**, **15c**, and **16a–c** bind within PTK by two hydrogen bonds. All 2,4-dioxo-1,2,3,4-tetrahydrobenzo-[*g*]pteridin-5-oxides (**13a–d**), 4-oxo-3,4-dihydrobenzo[*g*]pteridin-5-oxide (**16a–c**) exhibited two hydrogen bonds into the target PTK. These formed H bonds are presumably attributed to 4C═O, 5N–O, and N^3^H moieties, which enhance remarkably the binding affinities into PTKs.

An excellent correlation can be easily noticed for compounds **9e**, **10h**, and **12a,c–e**, where these compounds revealed binding free energies (Δ*G*_b_) being −13.30, −12.90, and −12.40 to −12.80 kcal/mol with corresponding biological antitumor activity against both of CCRF-HSB-2 and KB human tumour cell lines of IC_50_ of (0.87, 5.66, 5.86, and 6.53–8.34), and (0.47, 2.57, 6.11, and 6.45–8.36), (8.1–11.5 µg/mL), respectively.

**Figure 6.**
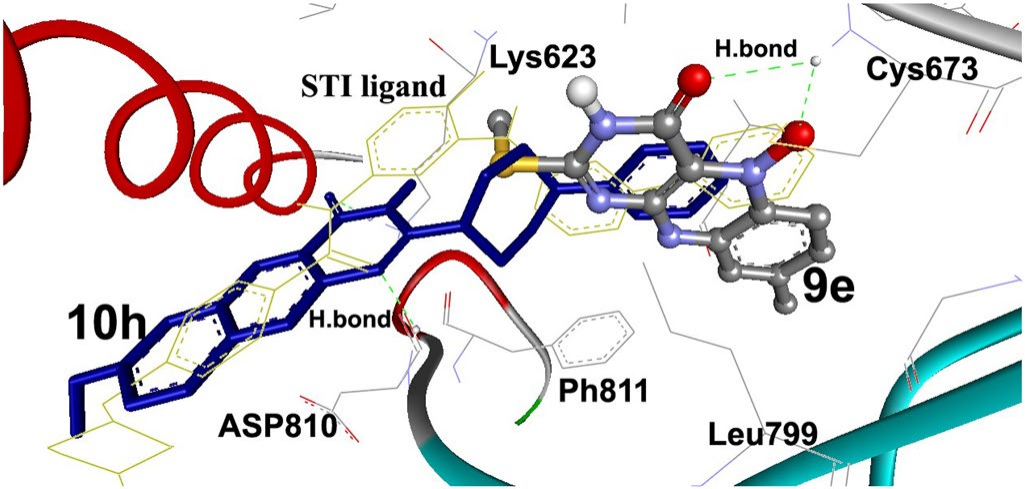
AutoDock differential binding affinities of the synthesised 8-methyl-2-methylthio-4-oxo-3,4-dihydrobenzo[*g*]pteridin-5-oxide (**9e**; coloured by element, ball, and stick), 2-(cyclohexyl amino)-benzo[*g*] pteridin-4(3*H*)-one (**10h**; blue stick) into PTK (1t46). Compounds **9e** and **10h** exhibited two hydrogen bonds. The amino acids of the binding site of the PTK are shown as labelled lines, coloured by the element, and the STI ligand is shown as dark yellow lines. The hydrogen bonds are shown as green dashed lines.

Compounds **9e** and **10h** were docked deeply embedded into the catalytic and activation loops of the ATP-binding cleft of the c-Kit receptor tyrosine kinase domain, revealing high and low binding energies in comparison with the docking of other derivatives of Δ*G*_b_ being −13.20 and −12.91 kcal/mol, respectively. Compound **9e** fits into the target site by two hydrogen bonds between its 4C═O and HN of Cys673 within RMSD of 7.44 Å. Also, compound **10h** binds by two hydrogen bonds between its 4C═O and N^10^ and HN of Lys623, and Asp810, respectively within RMSD of 0.12 Å (Figure 6).

**Figure 7.**
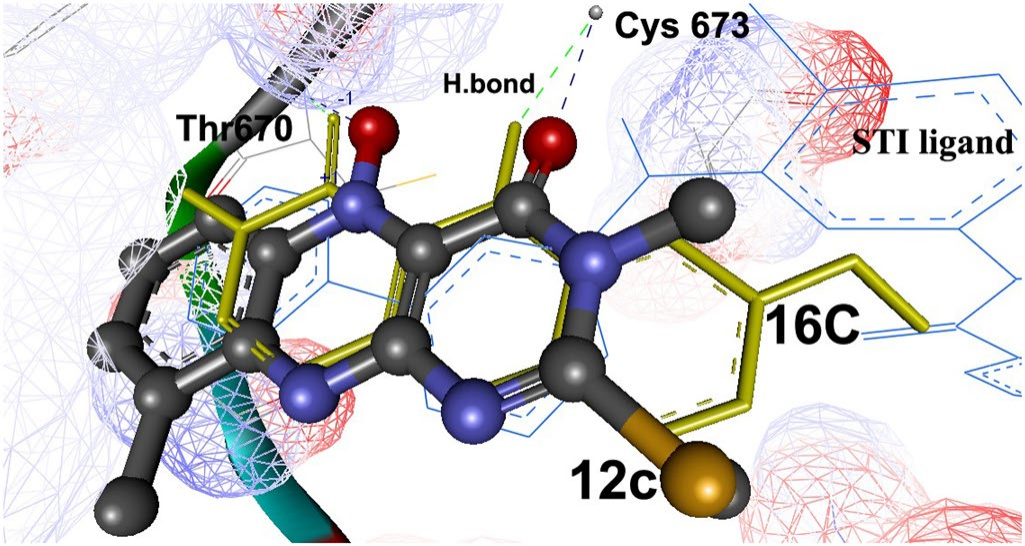
Stereo view for the mode of binding of 3-methyl-9-methyl-2-deoxo-2-methylthioalloxazin-5-oxide **(12c)** and 7-methoxy-4-oxo-3,4-dihydro-benzo[*g*]pteridin-5-oxide **(16c)**. They are embedded deeply into the catalytic and activation loops into the ATP-binding cleft of the c-Kit receptor PTK domain with its bound STI ligand. The docked compounds are shown in ball and stick, coloured by element and yellow sticks, and their hydrogen bonds between PTK are shown as blue and green dashed lines, respectively.

The docking of many compounds was identical especially those having similar biological activities, such as 3,9-dimethyl-2-methylthioalloxazin-5-oxide (**12c**) and 7-methoxy-4-oxo-3,4-dihydro- alloxazine-5-oxide (**16c**). Both revealed high binding energies Δ*G*_b_ being −12.80 and −11.70 kcal/mol, respectively, and seem as superimposed and docked overlapped on the native STI ligand within RMSD 6.58 and 5.11 Å, respectively, as shown in Figure 7. They fit into PTK binding site exhibiting two hydrogen bonds for both with Cys673 and Thr670 amino acids via their 4C═O and N–O moieties.

**Figure 8.**
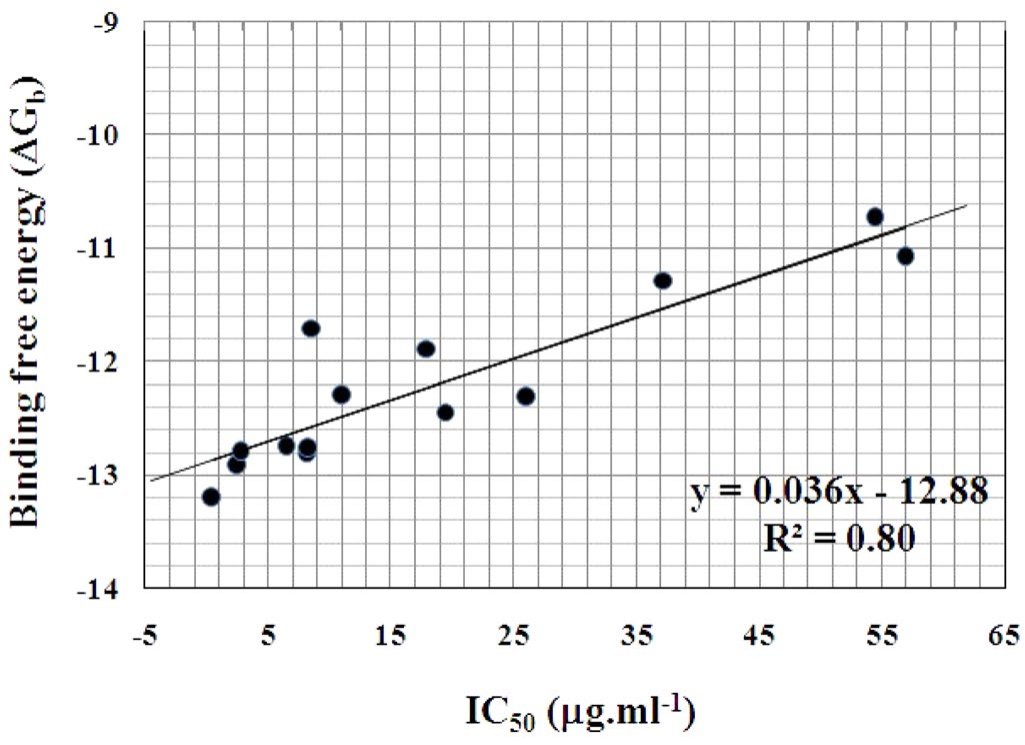
Correlation between the binding free energy (Δ*G*_b_) and IC_50_ (µg.mL^−1^) of 8-methyl-2-methylthio-4-oxo-alloxazine-5-oxide **(9e)**, 2-(substituted amino) alloxazines **(10a,c,f–i,l,n,q)**, 3-alkyl-2-deoxo-2-methylthio-alloxazin-5-oxides **(12c–e)**, and 7-methoxy-4-oxo-3,4-dihydro-alloxazine-5-oxide **(16c)** against human oral epidermoid carcinoma cell line (KB).

In the analysis of docking results, we tried to find a correlation between the biological results and docking studies. Whereas, the overall correlation between the IC_50_ (µg/mL) of the synthesised flavin analogues towards KB and CCRF-HSB-2 tumour cell lines and the binding affinities was very good for some compounds. Considering the growth inhibition against the human oral epidermoid carcinoma cell line (KB), it was noticed that the correlation between IC_50_ of compounds (**9e**, **10a,c,f–i,l,n,q**, **12c–e**, and **16c)** and their AutoDock binding free energies was good, revealing a correlation coefficient (*R*^2^) of 0.80 as represented in Figure 8.

**Figure 9.**
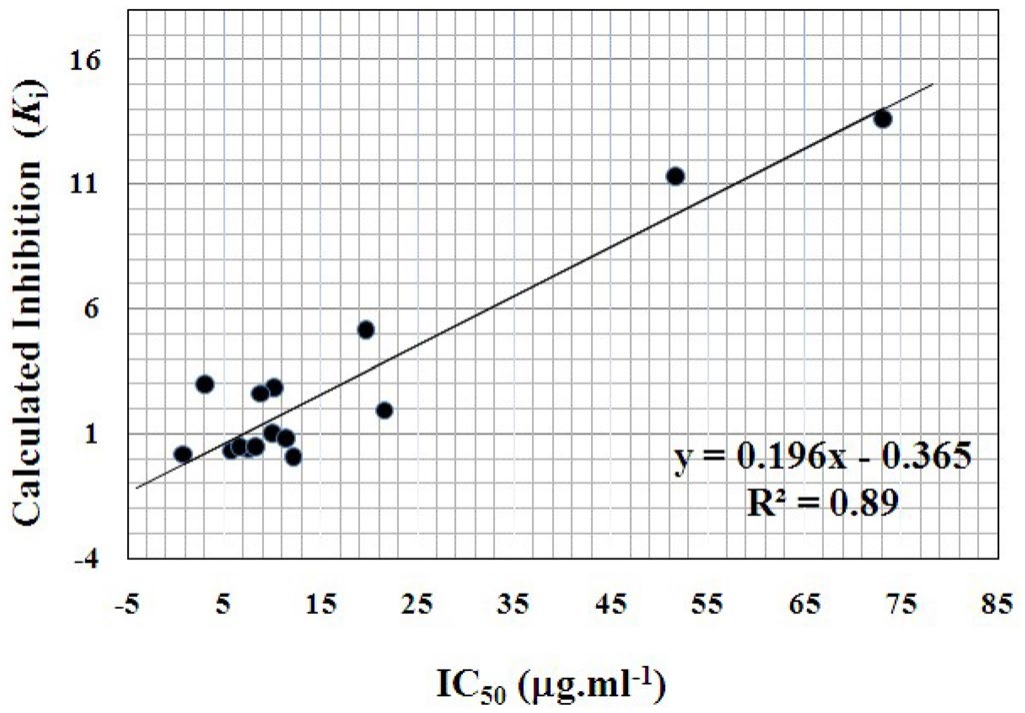
Correlation between calculated inhibition (*K_i_*) and IC_50_ (µg.mL^−1^) of 8-methyl-2-methylthio-4-oxo-alloxazine-5-oxide **(9e)**, 2-(substituted amino) alloxazines **(10g–i,k–m,q)**, 3-alkyl-2-deoxo-2-methylthio-alloxazin-5-oxides **(12b–e)**, 2-amino-9-methyl-alloxazine **(14a)**, and 4-oxo-3,4-dihydro alloxazine-5-oxides **(16b,c)** against human T-cell acute lymphoblastoid leukaemia cell line (CCRF-HSB-2).

Whereas, the growth inhibition against T-cell acute lymphoblastoid leukaemia cell line (CCRF-HSB-2), revealed a better correlation with Autodock binding free energies for compounds (**9e**, **10g–i,k–m,q**, **12b–e**, **14a**, and **16b,c**) of correlation coefficient (*R*^2^) of 0.89 as shown in Figure 9.

## Discussion

In our investigation utilising SBDD to explore various flavin analogues for the discovery of novel and specific antitumor agents^50^^,^^51^, the most promising antitumor activity was observed in compounds having the following structural characteristics: (1) the scaffold of flavin-5-oxide, 5-deazaflavin, and alloxazine, (2) SMe or NH_2_ group at the C2 position, (3) H (or alloxazine conformation) or Me group at the N10 position, and (4) unsubstituted quinoxaline nucleus or one substituted with a 7-Me or 9-Me group.

The pyrimidine ring of alloxazine-5-oxide is prone to nucleophilic substitution reactions at carbon atoms neighbouring the ring nitrogens. These reactions are well-documented and easily explained due to the electron-deficient nature of the π electrons. These reactions are well authenticated and readily explicable because of the π electron-deficient nature. The reactivity of the 2-methylthiopyrimidine containing oxo group in the 4-position can be rationalised since π electron deficient heterocycles containing hydroxyl groups in positions alpha to ring nitrogens are known to exist as cyclic lactams^52^. Hence, they are capable of electron-attracting properties which are apparent from canonical structures. This electron-attracting ability tends to favour nucleophilic substitution reactions; therefore, various aminations were applied for the replacement of 2-amino as shown in the preparation of the 2-amino-alloxazines (**14a,b**) and the 2-(substituted amino)-2-deoxo-alloxazine (**10a–q**).

For the first time in flavin chemistry, 2-unsubstituted 2-deoxo-alloxazine derivatives were prepared by dethiation of their corresponding 2-meththio analogues. The key precursors, 6-(N-anilino) pyrimidin-4(3*H*)-ones (**15a–c**), were synthesised by dethiation of 6-(N-anilino)-2-methylthio-3,4-dihydropyrimidine-4-ones (**8a,d,c**) using Raney nickel to afford yields of 58–72%.

UV/vis, IR, and NMR spectra and elemental analysis were used for the determination and identification of the newly assigned structures. The cyclised 2-amino-10-alkyl-4-oxo-4,10-dihydro-alloxazine-5-oxide (2-deoxy-2-aminoflavin-5-oxides) (**5a,b**) were differentiated from the non-cyclised compounds (**4a–e**) by the disappearance of the singlet signal of the proton at the 5-position in their ^1^H NMR spectra and loss of one ortho-aromatic proton by the oxidative cyclisation. The UV spectra of 2-deoxo-2-methylthio-alloxazin-5oxide (**9d,e**) exhibited longer wavelength than those of some 2-(substituted amino)-2-deoxo-alloxazin and this is attributed to the S-atom which causes a generally red shift (bathochromic shift) in the spectrum due to its easier polarisability^33^. The UV/vis absorption spectra of compounds (**10g,m**) exhibited five absorption maxima in the range of 212–470 nm, together with an absorption shoulder at 248–252 nm. It was observed that the 9-methyl analogue revealed more bathochromic shift at 470 nm as the Me group has an inductive effect + I effect and a mesomeric effect of the 2-NH group. The UV spectra of the **10i** compound exhibited three absorption maxima at 220–428 nm and this is due to the presence of the 7-methoxy group which has + M mesomeric effect and –I effect resulting in a change in the UV spectra and result in loss of two absorption maxima at 282–284 and 330–354 nm and loss of one shoulder at 248–252 nm.

Considering the ^1^H NMR spectra of 2-amino-2-deoxo-alloxazin derivatives (**14a,b**), they are characterised by the disappearance of the strong singlet signals of the 2-methylthio groups which were assigned for the starting compounds (**9d,c)** at *δ*_H_ 2.62–2.63 ppm, characteristic D_2_O exchangeable broad singlet signals at 6.80–7.02 ppm belong to exocyclic 2-NH_2_, broad singlet signals of 3-NH at *δ*_H_ 11.40–11.60 ppm, and the aromatic protons of the fused benzene ring appeared at *δ*_H_ 7.51–7.

Regarding the antitumor activity, the alloxazine analogues **10n** and **14a,b** exhibited highly selective antiproliferative activity against KB tumour cell lines. Their IC_50_ values for KB tumour cells are 2.72, 4.11, and 2.89 µg/mL, while for CCRF-HSB-2 tumour cells, the IC_50_ values are >100, 10.20, and 18.0 µg/mL, respectively.

The results considering SAR revealed that the highest antitumor activities were obtained with the structure features of alloxazine or alloxazin-5-oxide skeleton; SMe at the C-2 position and substituted quinoxaline nucleus by 8-Me group for compound **9e**, also the presence of cyclohexyl amino, butyl amino, benzyl amino, and amino substituent at C-2 position with unsubstituted quinoxaline nucleus or substituted by 7-OMe, or 9-Me group revealed good antitumor activity for compounds **10h,j,n** and **14b**, being of growth inhibitory activities against KB human tumour cell lines of IC_50_: 2.57, 2.13, 2.72, and 2.89 µg/mL, respectively. Also, derivatives with N^3^-ethyl substitution with 2-SMe and unsubstituted or substituted quinoxaline nucleus by the 7-Me group, namely **12a,b** exhibited good antitumor activity.

The SAR revealed moderate antitumor activity against both (CCRF-HSB-2) and (KB) human tumour cell lines of IC_50_ in the range of 7.7–12.1 µg/mL and 8.1–11.5 µg/mL, respectively. These potencies were obtained with the structure features of SMe, butyl amino, and *N*-phenyl piperazine at the C-2 position and similar features as cited before, as shown for compounds **9d**, **10i,g,m,p**, and **16c**. Also, derivatives with N^3^-ethyl substitution with 2-SMe and unsubstituted or substituted quinoxaline nucleus by 7-OMe, or 9-Me group, namely **12c,e** exhibited reasonable antitumor activity.

Regarding the protein kinase profiling, the synthesised compounds showed low to moderate inhibition (10–31%) against a panel of 20 kinases, including CAMK1, EGFR, FLT3, and SRC. CAMK1 exhibited consistent inhibition by all compounds, while Imatinib showed high inhibitions against ABL1 and c-KIT.

The predicted wlogP range for the compounds falls between 0.47 and 2.98, showing moderate lipophilicity. They also demonstrate reasonable water solubility and high GIT absorption.

Based on the information provided, compounds **10a**, **10c**, and **10f** exhibited differential inhibitory effects on KB cells without inducing toxic effects on CCRF-HSB-2 cell lines. Specifically, compound **10n** (with predicted LD50 = 1500 mg/kg) seems to be non-toxic to CCRF-HSB-2 cell lines and very potent against KB cells. This suggests that these compounds may have selective inhibitory effects against KB cells without affecting the viability of CCRF-HSB-2 cells. The predicted toxicity, PK, and drug-likeness criteria using ProTox-II and Swiss ADME software demonstrated that alloxazines (**10a–j**) had a toxicity class of 4 with LD50 of 825–1500 mg/kg, suggesting a favourable safety profile compared to MTX. However, compound **14b** and compound **16a**, with a toxicity class of 3 and predicted LD50 of 200–300 mg/kg, may have hepatotoxicity. The toxicity of compound **16a** could be attributed to its N-oxide features violating Brenk guidelines. This predicted toxicity of compound **16a** might be explained by its violation of Brenk guidelines with N-oxide, polycyclic aromatic HC (2), and quaternary nitrogen (1) structural features. Further research is necessary to validate these findings.

The *in silico* docking study was conducted to rationalise the obtained biological data and to explain the possible interactions between the tested derivatives into the crystal structure of the PTK enzyme (PDB: 1t46). Many of the docked compounds exhibited identical binding modes to the native bound ligand especially those having similar biological activities, such as compounds **12c** and **16c** with high binding energies. They fit into PTK binding site exhibiting two hydrogen bonds for both with Cys673 and Thr670 amino acids via their 4C═O and N–O moieties.

## Methods

### Chemistry

Melting points were determined using an electrothermal capillary melting point apparatus and were uncorrected. Infra-red (IR) spectra were recorded with a Perkin Elmer model 137 IR spectrophotometer, employing a KBr disc. Proton nuclear magnetic resonance (^1^H NMR) spectra were acquired using a Varian Mercury VXR-300 MHz spectrophotometer, with chemical shifts reported in delta (*δ*) values (parts per million) relative to tetramethylsilane (TMS) as an internal standard. Coupling constants are expressed in Hz. Exchangeable NH and OH protons were substituted with deuterium oxide (D_2_O). Mass spectra were obtained with a GCMS-QP 1000 EX Shimadzu Gas Chromatography-Mass Spectrometer, utilising electron impact (EI) at 70 eV. Elemental analyses were conducted using an Automatic CHN analyser, Vario E1Ш, at the Microanalytical Unit, Faculty of Science, Cairo University, Giza, Egypt.

All reagents used were commercially sourced and utilised without additional purification. Organic solvents were dehydrated with suitable drying agents and stored over appropriate molecular sieves.

Starting materials and reagents were procured from Sigma-Aldrich (St. Louis, MO) and Alfa Aesar (Ward Hill, MA) and utilised without additional purification. Various solvents were obtained from Sigma-Aldrich (St. Louis, MO) and Fisher Scientific (Waltham, MA) and employed directly without further purification. Chemicals including 3-(3,4-dimethylthiazol-2-yl)-2,5-diphenyltetrazolium bromide (MTT) were obtained from Sigma (St. Louis, MO). RPMI1640 media, penicillin/streptomycin, foetal bovine serum (FBS), trypsin–EDTA, l-glutamine, and dimethyl sulphoxide (DMSO) were obtained from El-Gomhouria Co. for Trading Medicines, Chemicals & Medical appliances (Shubra El Kheima, Egypt). The progression of reactions was monitored via analytical thin-layer chromatography (TLC) on silica gel 60F254-coated aluminium sheets (Merck, Rahway, NJ), and the resulting products were visualised under UV light *λ*_254_ nm.

### General procedure for the preparation of 2-aminopyrimidine-4,6-diol (1)

Sodium metal (**10g**, 0.43 mol) was dissolved in 256 mL of ethanol absolute; guanidine hydrochloride (40 g, 0.42 mol) was added portion wise with stirring for 1 h, then filtered. Another sodium ethoxide solution was prepared by dissolving sodium metal (19.2 g, 0.84 mol) in absolute ethanol (416 mL), to which was added diethyl malonate (67 g, 0.42 mol) and stirred for 5 min, then adding the guanidine solution. The reaction mixture was refluxed for 2 h, followed by evaporation *in vacuo* till dryness. The residue was taken up in water and pH was adjusted to 6 with acetic acid. The deposited product was filtered and washed with ethanol, then ether, and dried to be used in the next reaction step without further purification. Yield (48.0 g, 90%); m.p. >300 °C as reported^15^.

### Procedure for the preparation of 4,6-dichloropyrimidin-2-amine (2)

2-Aminopyrimidine-4,6-diol (**1**, 10.0 g, 0.079 mol) was mixed with phosphorous oxychloride (120.9 g, 0.79 mol); add triethylamine (17 mL, 3 equiv.). The reaction mixture was heated under reflux for 5–7 h, cooled to room temperature, and then poured with vigorous stirring into ice water bath. The yellow solid product was obtained by vacuum filtration and washed with water till neutral washings. Yield (9.85 g, 76%); m.p. 210–214 °C as reported^16^.

### Procedure for the preparation of 2-amino-6-chloropyrimidine-4-ol (3)

4,6-Dichloropyrimidin-2-amine (**2**, 10 g, 0.06 mol) was refluxed with sodium hydroxide solution (1 N, 122 mL) for 5 h, then the product was precipitated with acetic acid, filtered, and washed with water. Yield (7.77 g, 89%); m.p. 261 °C as reported^17^.

### General procedure for the preparation of 2-amino-6-(N-alkylanilino) pyrimidin-4(3H)-ones (4a,b)

A mixture of 2-amino-6-chloropyrimidine-4-ol (**3**, 2.0 g, 0.014 mol) and *N*-alkylaniline (0.069 mol) was fused at 190 °C for 7 h. After cooling, the crude product was treated with ether and washed with water. Yield (67–84%) as reported^18^.

#### 2-amino-6-(N-methyl anilino) pyrimidin-4(3H)-one (4a)

Yield (2.03 g, 67%); m.p. 274–276 °C, as reported^18^.

#### 2-amino-6-(N-ethylanilino)pyrimidin-4(3H)-one (4b)

Yield (2.7 g, 84%); m.p. 275–276 °C, as reported^18^.

### General procedure for the preparation of 2-amino-10-alkyl-4-oxo-4,10-dihydro-alloxazine-5-oxide (5a,b)

This standard protocol aligns with our previously published work^9^, wherein a clear solution of 2-amino-6-(*N*-alkylanilino) pyrimidin-4(3*H*)-one (**4**, 0.01 mol) in acetic acid (5–15 mL) was chilled in an ice bath to 10–15 °C. Sodium nitrite (0.02–0.04 mol) was added portion-wise to the cold solution during stirring, and the mixture was continued stirred at r.t. for 2–5 h. The separated solid product was filtered under vacuum and washed with water. The filtrate was condensed under vacuum and the residue was mixed with water or neutralised (pH 7) using aqueous ammonia to afford the second yield. The obtained crude product, dried and recrystallised from DMF–H_2_O to afford the desired product as red crystals in 62–66% yields.

#### 2-Amino-10-methyl-4-oxo-4,10-dihydrobenzo[g]pteridin-5-oxide (5a)

Yield (1.5 g, 62%, DMF/H_2_O); m.p. 200 °C (decomposed); UV (EtOH): *λ*_max_/nm (log *ε*/dm^3^ mol^−1^ cm^−1^): 250 (4.25), 274 (4.33), 340 (3.82), 456 (3.94); IR (*ν*_max_/cm^−1^): 3420, 3287 (imino NH), 1647 (C═O); ^1^H NMR [(CD3)_2_SO]: *δ* 4.07 (3H, s, 10-NMe), 4.24 (1H, br s, 2-imino exchangeable with D_2_O), 7.35–7.40 (1H, m, 7-H), 7.66–7.71 (1H, m, 8-H), 8.02–8.15 (1H, m, 9-H), 8.40–8.44 (1H, m, 6-H), 9.9 (1H, br s, 3-NH, exchangeable with D_2_O). Anal. calcd. for C_11_H_9_N_5_O_2_: C, 54.32; H, 3.73; N, 28.79. Found: C, 54.43; H, 3.92; N, 28.98. MS; EI, C_11_H_9_N_5_O_2_ *m/z* (R.A. %); (M-2) at 241 (20%), 77 (100%).

#### 2-Amino-10-ethyl-4-oxo-4,10-dihydrobenzo[g]pteridin-5-oxide (5b)

Yield (1.7 g, 66%, DMF/H_2_O), m.p. 230–232 °C (decomposed); UV (EtOH): *λ*_max_/nm (log *ε*/dm^3^ mol^−1^ cm^−1^): 244 (4.36), 274 (4.14), 334 (3.84), 452 (3.4); IR (*ν*_max_/cm^−1^): 3435, 3201 (imino NH), 1648 (C═O);^1^H NMR [(CD_3_)_2_SO]: *δ* 1.12–1.39 (3H, m, 10-NCH_2_-C*H_3_*), 4.0 (1H, br s, 2-imino exchangeable with D_2_O), 4.62–4.71 (2H, m, 10-NC*H_2_*-CH_3_), 7.07–7.15 (1H, m, 7-H), 7.20–7.34 (1H, m, 8-H), 7.85–8.18 (2H, m, 6,9-H), 10.0 (1H, br s, 3-NH, exchangeable with D_2_O). Anal. calcd. for C_11_H_11_N_3_OS: C, 56.63; H, 4.75; N, 18.01. Found: C, 56.40; H, 5.13; N, 18.23.

### Procedure for the preparation of 6-amino-2-thiouracil (6)

This compound was prepared according to the reported method^22–24^ by adding ethyl cyanoacetate (22.6 g) of sodium ethoxide solution; prepared by dissolving 9.0 g sodium metal in 200 mL absolute ethanol. After 15 min., thiourea (15.2 g, 0.2 mol) was added portionwise with shaking and the mixture was allowed to stand at room temperature for one hour with occasional shaking. The reaction mixture was refluxed for 2 h, and the white precipitate formed was collected by filtration then dissolved in boiling diluted potassium hydroxide and reprecipitated with acetic acid to afford the product which crystallised from ethanol to afford 22 g, 77% yield; m.p. >300 °C as reported.

### General procedure for the preparation of 2,3-dihydro-6-(N-anilino)-2-thioxopyrimidin-4(1H)-ones (7a–e)

6-Amino-2-thiouracil (**6**, 7.10 g, 0.05 mol), was mixed with aniline (0.1 mol) and anilinium chloride (0.05 mol), then heated to fusion at 170 °C for 9 h. The treated mixture was diluted with EtOH (65%; 200 mL). The separated solid product was filtered, treated with diethyl ether, dried and then washed with water. The collected residue was dissolved in 5% NaOH solution under a steam bath, and reprecipitated by neutralisation using 10% HCl. The separated solid product was filtrated off, washed, dried, and crystallised from DMF/H_2_O to afford colourless crystals of the product in 38–77% yields.

#### 2,3-Dihydro-6-(N-anilino)-2-thioxopyrimidin-4(1H)-one (7a)

Yield (6 g, 55%, DMF–H_2_O), (lit.^19^: m.p. 296–298 °C (decomposed)).

#### 2,3-Dihydro-6-(p-tolylamino)-2-thioxopyrimidin-4(1H)-one (7b)

Yield (7.82 g, 67%, DMF–H_2_O), (lit.^19^: m.p. 293–295 °C (decomposed)).

#### 2,3-Dihydro-6-(p-anisidino)-2-thioxopyrimidin-4(1H)-one (7c)

Yield (9.60 g, 77%, DMF–H_2_O), (lit.^19^: m.p. 284–286 °C (decomposed)).

#### 2,3-Dihydro-6-(o-tolylamino)-2-thioxopyrimidin-4(1H)-one (7d)

Yield (8.0 g, 69%, DMF–H_2_O), m.p. 178–180 °C (decomposed); UV (EtOH): *λ*_max_/nm (log *ε*/dm^3^ mol^−1^ cm^−1^): 272 (4.34); IR (*ν*_max_/cm^−1^): 3288, 3185, 3090 (NH), 1633 (C═O); ^1^H NMR [(CD_3_)_2_SO]: *δ* 2.19 (3H, s, 2′-Me), 4.45 (1H, s, 5-H), 7.23–7.33 (4H, m, Ar-*o,m, p-*H), 7.85 (1H, s, 6-NH, exchangeable with D_2_O), 11.63 (1H, s, 1-NH, exchangeable with D_2_O), 11.84 (1H, s, 3-NH, exchangeable with D_2_O). Anal. calcd. for C_11_H_11_N_3_OS: C, 56.63; H, 4.75; N, 18.01. Found: C, 56.87; H, 5.13; N, 18.18.

#### 2,3-Dihydro-6-(m-tolylamino)-2-thioxopyrimidin-4(1H)-one (7e)

Yield (4.5 g, 39%, DMF–H_2_O), m.p. 282–284 °C; UV (EtOH): *λ*_max_/nm (log *ε*/dm^3^ mol^−1^ cm^−1^): 278 (4.39); IR (*ν*_max_/cm^−1^): 3324, 3170, 3143 (NH), 1663 (C═O); ^1^H NMR [(CD_3_)_2_SO]: *δ* 2.27 (3H, s, 3′-Me), 4.94 (1H, s, 5-H), 7.02 (1H, m, 5′-H), 7.26–7.28 (2H, m,4′,6′-H), 7.31 (1H, br s, 2′-H), 8.12 (1H, s, 6-NH, exchangeable with D_2_O), 11.49 (1H, s, 1-NH, exchangeable with D_2_O), 11.88 (1H, s, 3-NH, exchangeable with D_2_O). Anal. calcd. for C_11_H_11_N_3_OS: C, 56.63; H, 4.75; N, 18.01. Found: C, 56.27; H, 4.93; N, 17.73.

### General procedure for the preparation of 6-N-anilino-2-methylthio-3,4-dihydropyrimidine-4-ones (8a–e)

A solution containing 6-*N*-anilino-4-oxo-2-thioxo-1,2,3,4-tetrahydropyrimi-dines (**7a–e**, 0.01 mol) in 2 N NaOH (250 mL) was cooled in an ice bath to 0–5 °C. Methyl iodide (2.84 g, 0.02 mol) was added slowly in portions, and the solution was vigorously shaken while being kept cold for 30 min. The solid precipitate was filtered, then the filtrate was then neutralised with 10% HCl to get the second portion. The resulting precipitate was dissolved again in hot 5% NaOH solution and reprecipitated by adding 10% HCl to obtain pure products. The crude product was dried and subjected to recrystallisation from an appropriate solvent, yielding the desired product as silver-coloured crystals in yields ranging from 69 to 94%.

#### 6-N-Anilino-2-methylthio-3,4-dihydropyrimidin-4-one (8a)

Yield (2.2 g, 94%); m.p. 294–295 °C, as reported^19^.

#### 6-N-(p-tolylamino)-2-methylthio-3,4-dihydropyrimidin-4-one (8b)

Yield (1.80 g, 73%); m.p. 252–254 °C, as reported^19^.

#### 6-N-(p-anisidino)-2-methylthio-3,4-dihydropyrimidin-4-one (8c)

Yield (2.24 g, 85%); m.p. 229–231 °C, as reported^19^.

#### 6-N-(o-tolylamino)-2-methylthio-3,4-dihydropyrimidin-4-one (8d)

Yield (2.20 g, 89%, DMF–H_2_O): m.p. 256–258 °C; UV (EtOH): *λ*_max_/nm (log *ε*/dm^3^ mol^−1^ cm^−1^): 246 (4.33), 300 sh (3.82); IR (*ν*_max_/cm^−1^): 3238, 3138 (NH), 1627 (C═O); ^1^H NMR [(CD_3_)_2_SO]: *δ* 2.20 (3H, s, 2′-Me), 2.43 (3H, s, 2-SMe), 4.80 (1H, s, 5-H), 7.00–7.20 (4H, m, Ar-H), 8.50 (1H, s, 6-NH, exchangeable with D_2_O), 11.7 (1H, br s, 3-NH, exchangeable with D_2_O). Anal. calcd. for C_12_H_13_N_3_OS: C, 58.28; H, 5.30; N, 16.99. Found: C, 58.45; H, 5.68; N, 17.19.

#### 6-N-(m-tolylamino)-2-methylthio-3,4-dihydropyrimidin-4-one (8e)

Yield (1.70 g, 69%, DMF–H_2_O): m.p. 250–252 °C; UV (EtOH): *λ*_max_/nm (log *ε*/dm^3^ mol^−1^ cm^−1^): 258 (4.34), 304 sh (4.10); IR (*ν*_max_/cm^−1^): 3259, 3139 (NH), 1631 (C═O); ^1^H NMR [(CD_3_)_2_SO]: *δ* 2.30 (3H, s, 3′-Me), 2.52 (3H, s, 2-SMe), 5.3 (1H, s, 5-H), 6.80–6.90 (1H, m, 5′-H), 7.20–7.30 (2H, m, 4′,6′-H), 7.35 (1H,br s, 2′-H), 9.10 (1H, s, 6-NH, exchangeable with D_2_O), 11.90 (1H, s, 3-NH, exchangeable with D_2_O). Anal. calcd. for C_12_H_13_N_3_OS: C, 58.28; H, 5.30; N, 16.99. Found: C, 58.52; H, 5.52; N, 16.63.

### General procedure for the preparation of 2-deoxo-2-methyl-thio-alloxazin-5-oxides (2-methylthio-4-oxo-3,4-dihydrobenzo[g]pteridin-5-oxides) (9a–e)

Sodium nitrite (0.02–0.04 mol) was gradually added to a stirred solution containing 6-anilino-2-methylthio-4-oxo-3,4-dihydropyrimidines (**8**, 0.01 mol) in acetic acid (5–15 mL) while maintaining an ice bath at 10–15 °C. The mixture was then stirred at r.t. with occasional warming in a water bath for 2–5 h to facilitate cyclisation. The resulting solid precipitate was filtered and washed with water. The filtrate was then concentrated under vacuum, and the residue was treated with water or neutralised (pH 7) by aqueous solution of ammonia to obtain the second crop. The respective product was crystallised from a mixture of DMF/H_2_O, resulting in yellow-orange crystals of the titled compound with yields ranging from 62% to 89%.

#### 2-Methylthio-4-oxo-3,4-dihydrobenzo[g]pteridin-5-oxide (9a)

Yield (2.32 g, 89%); m.p. >300 °C, as reported^19^.

#### 7-Methyl-2-methylthio-4-oxo-3,4-dihydrobenzo[g]pteridine-5-oxide (9b)

Yield (1.81 g, 66%); m.p. >300 °C, as reported^19^.

#### 7-Methoxy-2-methylthio-4-oxo-3,4-dihydrobenzo[g]pteridin-5-oxide (9c)

Yield (2.38 g, 82%); m.p. >300 °C, as reported^19^.

#### 9-Methyl-2-methylthio-4-oxo-3,4-dihydrobenzo[g]pteridin-5-oxide (9d)

Yield (1.95 g, 71%, DMF); m.p. 250–251 °C; UV (EtOH): *λ*_max_/nm (log *ε*/dm^3^ mol^−1^ cm^−1^): 250 sh (4.3), 272 (4.46), 296 (4.39), 360 (3.73), 448 (3.72); IR (*ν*_max_/cm^−1^): 3184 (NH), 1695 (C═O); ^1^H NMR [(CD_3_)_2_SO]: *δ* 2.54 (3H, s, 9-Me), 2.62 (3H, s, 2-SMe), 7.47–7.49 (1H, m, 7-H), 7.60–7.70 (1H, m, 8-H), 8.10–8.20 (1H, m, 6-H), 12.80 (1H, br s, 3-NH). Anal. calcd. for C_12_H_10_N_4_O_2_S: C, 52.54; H, 3.67; N, 20.43. Found: C, 52.75; H, 3.50; N, 20.55. MS; EI, C_12_H_10_N_4_O_2_S *m/z* (R.A. %): 274 (M^+^, 42%), 257 (100%), 183 (40%), 156 (32%), 129 (18%).

#### 8-Methyl-2-methylthio-4-oxo-3,4-dihydrobenzo[g]pteridin-5-oxide (9e)

Yield (1.70 g, 62%, DMF–H_2_O); m.p. 262–264 °C; UV (EtOH): *λ*_max_/nm (log *ε*/dm^3^ mol^−1^ cm^−1^): 246 sh (4.26), 272 (4.5), 294 (4.47), 364 (3.87), 442 (3.75); IR (*ν*_max_/cm^−1^): 3176 (NH), 1687 (C═O); ^1^H NMR [(CD_3_)_2_SO]: *δ* 2.60 (3H, s, 8-Me), 2.63 (3H, s, 2-SMe), 7.40–8.30 (3H, m, 6,7,8-H), 12.80 (1H, br s, 3-NH). Anal. calcd. C_12_H_10_N_4_O_2_S. C, 52.54; H, 3.67; N, 20.43. Found: C, 52.75; H, 3.50; N, 20.55.

### General procedure for the preparation 2-(substituted amino)-alloxazines (10a–q)

A solution containing 2-methylthio-4-oxo-3,4-dihydrobenzo[*g*]pteridine-5-oxide derivative (9, 0.01 mol) and an appropriate quantity of amine (0.1–0.2 mol) in DMF (30 mL) was heated under reflux for 5–14 h (monitored by TLC). Yellow crystals were obtained and collected after refrigerating the solution overnight, followed by filtration to obtain the first crop. The filtrate was then concentrated under vacuum to remove excess amine, and the residue was washed with ether and water to obtain a second crop devoid of amine. The isolated solid product was dried and subjected to crystallisation from either DMF or a DMF/water mixture, resulting in pure products as yellow needles in 44–87% yields.

#### 2-(Morpholin-4-yl)benzo[g]pteridin-4(3H)-one (10a)

Yield (1.90 g, 67%, DMF); m.p. >300 °C; UV (EtOH): *λ*_max_/nm (log *ε*/dm^3^ mol^−1^ cm^−1^): 222 (4.43), 270 (4.48), 286 sh (4.38), 342 (3.74), 438 (3.98); IR (*ν*_max_/cm^−1^): 3167 (NH), 1680 (C═O); ^1^H NMR [(CD_3_)_2_SO]: *δ* 3.70–3.74 (4H, m, 3′ and 5′-NCH_2_), 3.78–3.82 (4H, m, 2′ and 6′-OCH_2_), 7.73–7.80 (1H, m, 7-H), 7.91–7.98 (2H, m, 6, 8-H), 8.10–8.20 (1H, m, 9-H), 11.90 (1H, br s, 3-NH, exchangeable with D_2_O). Anal. calcd. C_14_H_13_N_5_O_2_: C, 59.36; H, 4.63; N, 24.72. Found: C, 59.75; H, 4.95; N, 25.10. MS; EI, C_14_H_13_N_5_O_2_ *m/z* (R.A. %): (M + 1) at 284 (33%), 226 (80%), 170 (100%).

#### 2-(Pipridin-1-yl)benzo[g]pteridin-4(3H)-one (10b)

Yield (1.80 g, 64%, DMF–H_2_O); m.p. 300 °C; UV (EtOH): *λ*_max_/nm (log *ε*/dm^3^ mol^−1^ cm^−1^): 216 (4.47), 252 sh (4.13), 272 (4.29), 290 (4.3), 336 (3.68), 430 (3.79); IR (*ν*_max_/cm^−1^): 3179 (NH), 1682 (C═O); ^1^H NMR [CDCl_3_]: *δ* 1.65–1.78 (6H, m, 3′,4′ and 5′-CH_2_), 3.82–3.90 (4H, m, 2′ and 6′-NCH_2_), 7.68 (1H, t, *J*_6,7_ = *J*_7,8_ = 7.8 Hz, 7-H), 7.80 (1H, t, *J*_7,8_ = *J*_8,9_ = 7.8 Hz, 8-H), 8.0 (1H, d, *J*_6,7_ = 7.8 Hz, 6-H), 8.20 (1H, br d, *J*_8,9_ = 7.8 Hz, 9-H), 11.47 (1H, br s, 3-NH, exchangeable with D_2_O). Anal. calcd. C_15_H_15_N_5_O: C, 64.04; H, 5.37; N, 24.90. Found: C, 63.86; H, 5.73; N, 24.74. MS; EI, C_15_H_15_N_5_O *m/z* (R.A. %): (M^+^) at 281 (M^+^, 52%), 169 (43%), 84 (100%).

#### 7-Methyl-2-(morpholin-4-yl)benzo[g]pteridin-4(3H)-one (10c)

Yield (1.65 g, 56%, DMF–H_2_O); m.p. >300 °C; UV (EtOH): *λ*_max_/nm (log *ε*/dm^3^ mol^−1^ cm^−1^): 226 (4.37), 250 sh (4.21), 272 (4.39), 286 (4.36), 346 (3.8), 428 (3.87); IR (*ν*_max_/cm^−1^): 3195 (NH), 1698 (C═O); ^1^H NMR [(CD_3_)_2_SO]: *δ* 2.57 (3H, s,7-Me), 3.71–3.73 (4H, m, 3′ and 5′-NCH_2_), 3.75–3.85 (4H, m, 2′ and 6′-OCH_2_), 7.60–8.0 (3H, m, 6,8,9-H), 11.80 (1H, br s, 3-NH, exchangeable with D_2_O). Anal. calcd. For C_15_H_15_N_5_O_2_: C, 60.60; H, 5.09; N, 23.56. Found: C, 60.72; H, 5.45; N, 23.81.

#### 8-Methyl-2-(morpholin-4-yl)benzo[g]pteridin-4(3H)-one (10d)

Yield (2.0 g, 67%, DMF–H_2_O); m.p. >300 °C; UV (EtOH): *λ*_max_/nm (log *ε*/dm^3^ mol^−1^ cm^−1^): 222 (4.38), 272 (4.37), 286 sh (4.21), 338 (3.62), 440 (3.79); IR (*ν*_max_/cm^−1^): 3148 (NH), 1696 (C═O); ^1^H NMR [(CD_3_)_2_SO]: *δ* 2.77 (3H, s, 8-Me), 3.70–3.72 (4H, m, 3′ and 5′-NCH_2_), 3.76–3.78 (4H, m, 2′ and 6′-OCH_2_), 7.60–8.0 (3H, m, 6,7,9-H), 11.87 (1H, br s, 3-NH, exchangeable with D_2_O). Anal. calcd. C_15_H_15_N_5_O_2_: C, 60.60; H, 5.09; N, 23.56. Found: C, 60.29; H, 4.61; N, 23.33.

#### 9-Methyl-2-(piperidin-1-yl)benzo[g]pteridin-4(3H)-one (10e)

Yield (1.70 g, 58%, DMF); m.p. >300 °C; UV (EtOH): *λ*_max_/nm (log *ε*/dm^3^ mol^−1^ cm^−1^): 224 (4.4), 254 sh (4.35), 272 (4.47), 294 (4.42), 348 (3.91), 436 (3.88); IR (*ν*_max_/cm^−1^): 3152 (NH), 1701 (C═O); ^1^H NMR [CDCl_3_]: *δ* 1.63–1.75 (2H, m, 4′-CH_2_), 2.68 (3H, s, 9-Me), 3.45–3.50 (4H, m, 3′ and 5′-CH_2_), 3.75–3.82 (4H, m, 2′ and 6′-CH_2_), 7.60–7.90 (3H, m, 6,7,8-H), 11.80 (1H, br s, 3-NH, exchangeable with D_2_O). Anal. calcd. C_16_H_17_N_5_O: C, 65.07; H, 5.80; N, 23.71. Found: C, 70.48; H, 5.92; N, 31.92.

#### 9-Methyl-2-(morpholin-4-yl)benzo[g]pteridin-4(3H)-one (10f)

Yield (1.30 g, 44%, DMF); m.p. >300 °C; UV (EtOH): *λ*_max_/nm (log *ε*/dm^3^ mol^−1^ cm^−1^): 224 (4.38), 254 sh (4.29), 270 (4.45), 286 (4.39), 346 (3.82), 432 (3.94); IR (*ν*_max_/cm^−1^): 3166 (NH), 1700 (C═O); ^1^H NMR [(CD_3_)_2_SO]: *δ* 2.66 (3H, s, 9-Me), 3.70–3.72 (4H, m, 3′ and 5′-NCH_2_), 3.76–3.78 (4H, m, 2′ and 6′-OCH_2_), 7.58 (1H, t, *J*_6,7_ = *J*_7,8_ = 8.1 Hz, 7-H), 7.66 (1H, br d, *J*_7,8_ = 8.1 Hz, 8-H), 7.92 (1H, br d, *J*_6,7_ = 8.1 Hz, 6-H), 11.80 (1H, br s, 3-NH, exchangeable with D_2_O). Anal. calcd. C_15_H_15_N_5_O_2_: C, 60.60; H, 5.09; N, 23.56. Found: C, 60.94; H, 5.40; N, 23.35.

#### 2-(Butylamino)benzo[g]pteridin-4(3H)-one (10g)

Yield (2.0 g, 74%, DMF–H_2_O); m.p. 288–290 °C; UV (EtOH): *λ*_max_/nm (log *ε*/dm^3^ mol^−1^ cm^−1^): 222 (4.44), 248 sh (4.29), 264 (4.42), 282 (4.39), 330 (3.68), 412 (3.84); IR (*ν*_max_/cm^−1^): 3426, 3235 (NH), 1710 (C═O); ^1^H NMR [(CD_3_)_2_SO]: *δ* 0.98 (3H, t, 2-NH-(CH_2_)_3_-*CH_3_*), 1.40–1.50 (2H, m, 2-NH-(CH_2_)_2_-*CH_2_*-CH_3_), 1.58–1.65 (2H, m, 2-NH-CH_2_-*CH_2_*-CH_2_-CH_3_), 3.30–3.55 (2H, m, 2-NH-*CH_2_*-(CH_2_)_2_-CH_3_), 6.90 (1H, br s, 2-NH, exchangeable with D_2_O), 7.70–8.10 (4H, m, 6,7,8,9-H), 11.40 (1H, br s, 3-NH, exchangeable with D_2_O). Anal. calcd. C_14_H_15_N_5_O: C, 62.44; H, 5.61; N, 26.01. Found: C, 62.21; H, 6.00; N, 25.80.

#### 2-(Cyclohexylamino)benzo[g]pteridin-4(3H)-one (10h)

Yield (1.80 g, 61%, DMF–H_2_O); m.p. 168–170 °C; UV (EtOH): *λ*_max_/nm (log *ε*/dm^3^ mol^−1^ cm^−1^): 222 (4.43), 270 (4.47), 338 (3.79), 436 (3.86); IR (*ν*_max_/cm^−1^): 3376, 3286 (NH), 1699 (C═O); ^1^H NMR [CDCl_3_]; *δ* 1.30–1.90 (6H, m, 3′, 4′, and 5′-CH_2_), 2.02–2.18 (4H, m, 2′ and 6′-CH_2_), 4.06–4.21 (1H, m,1′-CH), 7.70–8.10 (4H, m, 6,7,8,9-H), 4.21 (1H, br s, 2-NH, exchangeable with D_2_O), 13.50 (1H, br s, 3-NH, exchangeable with D_2_O). Anal. calcd. for C_16_H_17_N_5_O: C, 65.07; H, 5.80; N, 23.71. Found: C, 65.27; H, 6.16; N, 23.89.

#### 2-(Butyl amino)-7-methoxybenzo[g]pteridin-4(3H)-one (10i)

Yield (1.60 g, 54%, DMF–H_2_O); m.p. 186–188 °C; UV (EtOH): *λ*_max_/nm (log *ε*/dm^3^ mol^−1^ cm^−1^): 220 (4.33), 272 (4.45), 428 (3.85); IR (*ν*_max_/cm^−1^): 3400, 3220 (NH), 1703 (C═O); ^1^H NMR [(CD_3_)_2_SO]: *δ* 0.93 (3H, t, *J* = 7.2 Hz, 2-NH-(CH_2_)_3_-*CH_3_*), 1.39 (2H, hextet, *J* = 7.2 Hz, 2-NH-(CH_2_)_2_-*CH_2_*-CH_3_), 1.57 (2H, quintet, *J* = 7.2 Hz, 2-NH-CH_2_-*CH_2_*-CH_2_-CH_3_), 3.37–3.41 (2H, m, 2-NH-*CH_2_*-(CH_2_)_2_-CH_3_), 3.93 (3H, s,7-OMe), 6.70 (1H, br s, 2-NH, exchangeable with D_2_O), 7.40–7.80 (3H, m, 6,8 and 9-H), 11.2 (1H, br s, 3-NH, exchangeable with D_2_O). Anal. calcd. C_15_H_17_N_5_O_2_.0.2DMF: C, 59.68; H, 5.91; N, 23.20. Found: C, 56.59; H, 4.61; N, 23.33.

#### 2-(Cyclohexylamino)-7-methoxybenzo[g]pteridin-4(3H)-one (10j)

Yield (1.80 g, 55%, DMF–H_2_O); m.p. 200 °C; UV (EtOH): *λ*_max_/nm (log *ε*/dm^3^ mol^−1^ cm^−1^): 224 (4.36), 276 (4.56), 330 (3.63), 452 (3.88); IR (*ν*_max_/cm^−1^): 3396, 3242 (NH), 1697 (C═O); ^1^H NMR [CDCl_3_]: *δ* 1.21–1.30 (2H, m, 4′-CH_2_), 1.42–1.55 (4H, m, 3′ and 5′-CH_2_), 1.85–1.99 (4H, m, 2′ and 6′-CH_2_), 3.72 (3H, s, 7-OCH_3_), 3.94–5.10 (1H, m, 1′-CH), 7.70–8.10 (3H, m, 6,8,9-H), 5.0 (1H, br s, 2-NH, exchangeable with D_2_O), 13.10 (1H, br s, 3-NH, exchangeable with D_2_O). Anal. calcd. C_17_H_19_N_5_O_2_: C, 62.75; H, 5.89; N, 21.52. Found: C, 63.07; H, 6.21; N, 21.40.

#### 2-(Butyl amino)-7-methylbenzo[g]pteridin-4(3H)-one (10k)

Yield (1.95 g, 69%, DMF–H_2_O); m.p. 214–216 °C; UV (EtOH): *λ*_max_/nm (log *ε*/dm^3^ mol^−1^ cm^−1^): 224 (4.43), 272 (4.48), 286 sh (4.28), 336 (3.71), 442 (3.86); IR (*ν*_max_/cm^−1^): 3394, 3246 (NH), 1704 (C═O); ^1^H NMR [(CD_3_)_2_SO]: *δ* 1.25–1.42 (3H, m, 2-NH-(CH_2_)_3_-*CH_3_*), 1.80–1.90 (2H, m, 2-NH-(CH_2_)_2_-*CH_2_*-CH_3_), 2.01–2.20 (2H, m, 2-NH-CH_2_-*CH_2_*-CH_2_-CH_3_), 2.95 (3H, s, 7-Me), 3.81–3.96 (2H, m, 2-NH-*CH_2_*-(CH_2_)_2_-CH_3_), 7.40 (1H, br s, 2-NH, exchangeable with D_2_O), 7.80–7.90 (1H, m, 9-H), 8.0–8.20 (2H, m,6 and 8-H), 11.8 (1H, br s, 3-NH, exchangeable with D_2_O). Anal. calcd. C_15_H_17_N_5_O: C, 63.59; H, 6.05; N, 24.72. Found: C, 63.40; H, 6.43; N, 24.97.

#### 2-(Cyclohexylamino)-7-methylbenzo[g]pteridin-4(3H)-one (10l)

Yield (1.90 g, 62%, DMF–H_2_O); m.p. 248–250 °C; UV (EtOH): *λ*_max_/nm (log *ε*/dm^3^ mol^−1^ cm^−1^): 224 (4.43), 272 (4.53), 338 (3.77), 448 (3.94); IR (*ν*_max_/cm^−1^): 3398, 3229 (NH), 1695 (C═O); ^1^H NMR [CDCl_3_]: *δ* 0.95–1.14 (2H, m, 4′-CH_2_), 1.42–1.55 (4H, m, 3′ and 5′-CH_2_), 1.84–1.95 (4H, m, 2′ and 6′-CH_2_), 2.24 (3H, s, 7-Me), 3.80–4.10 (1H, m, 1′-CH), 6.86–7.62 (3H, m, 6,8,9-H), 4.95 (1H, br s, 2-NH, exchangeable with D_2_O), 12.50 (1H, br s, 3-NH, exchangeable with D_2_O). Anal. calcd. C_17_H_19_N_5_O: C, 66.00; H, 6.19; N, 22.64. Found: C, 66.37; H, 6.52; N, 22.36.

#### 2-(Butyl amino)-9-methylbenzo[g]pteridin-4(3H)-one (10m)

Yield (1.50 g, 53%, DMF); m.p. 190–192 °C; UV (EtOH): *λ*_max_/nm (log *ε*/dm^3^ mol^−1^ cm^−1^): 212 (4.3), 252 sh (4.24), 272 (4.48), 284 (4.29), 354 (3.71), 470 (3.77); IR (*ν*_max_/cm^−1^): 3407, 3234 (NH), 1645 (C═O); ^1^H NMR [(CD_3_)_2_SO]: *δ* 0.89–0.97 (3H, m, 2-NH-(CH_2_)_3_-*CH_3_*), 1.40 (2H, hextet, *J* = 7.2, 2-NH-(CH_2_)_2_-*CH_2_*-CH_3_), 1.59 (2H, quintet, *J* = 7.2, 2-NH-CH_2_-*CH_2_*-CH_2_-CH_3_), 2.62 (3H, s, 9-Me), 3.54–3.61 (2H, m, 2-NH-*CH_2_*-(CH_2_)_2_-CH_3_), 7.67 (1H, br s, 2-NH, exchangeable with D_2_O), 7.37–7.47 (1H, m, 7-H), 7.63 (1H, br d, *J_7,8_* = 7.6 Hz, 8-H), 8.15 (1H, br d, *J_6,7_* = 7.6 Hz, 6-H), 10.67 (1H, br s, 3-NH, exchangeable with D_2_O). Anal. calcd. C_15_H_17_N_5_O: C, 63.59; H, 6.05; N, 24.72. Found: C, 63.88; H, 6.43; N, 24.96.

#### 2-(Benzyl amino)-9-methylbenzo[g]pteridin-4(3H)-one (10n)

Yield (2.0 g, 63%, DMF); m.p. 238–240 °C; UV (EtOH): *λ*_max_/nm (log *ε*/dm^3^ mol^−1^ cm^−1^): 224 (4.5), 256 sh (4.46), 270 (4.54), 280 (4.43), 344 (3.87), 442 (4.03); IR (*ν*_max_/cm^−1^): 3390, 3220 (NH), 1610 (C═O); ^1^H NMR [(CDCl_3_]: *δ* 2.78 (3H, s, 9-Me), 4.69 (2H, s, 2-NH-*CH_2_*-Ph), 7.26–7.39 (5H, m, *o*,*m,p*Ph-H), 7.59–7.85 (3H, m, 6,7,8-H), 8.20 (1H, br s, 2-NH, exchangeable with D_2_O), 9.40 (1H, br s, 3-NH, exchangeable with D_2_O). Anal. calcd. for C_18_H_15_N_5_O: C, 68.13; H, 4.76; N, 22.07. Found: C, 68.00; H, 5.15; N, 22.25.

#### 2-(4-Phenylpiperazin-1-yl)benzo[g]pteridin-4(3H)-one (10o)

Yield (3.10 g, 87%, DMF); m.p. >300 °C; UV (EtOH): *λ*_max_/nm (log *ε*/dm^3^ mol^−1^ cm^−1^): 220 sh (4.52), 254 (4.51), 270 (4.53), 284 (4.49), 338 (3.75), 436 (3.94); IR (*ν*_max_/cm^−1^): 3426 (OH tautomer of C═O); ^1^H NMR [(CD_3_)_2_SO]: *δ* 3.16–3.23 (4H, m, 2′ and 6′-NCH_2_), 3.45 (1H, br s, 4-OH, exchangeable with D_2_O); 3.96–3.99 (4H, m, 3′ and 5′-NCH_2_), 6.75 (1H, dt, *J*_3″,4″_ = *J*_4″,5″_ = 5.1 Hz, *J*_2″,4″_ = *J*_4″,6″_ = 2.1 Hz, Ph-*p*H), 6.91–6.98 (2H,m, Ph-*o*H), 7.10–7.30 (2H, m, Ph-*m*H), 7.60 (1H,t, *J*_6,7_ = *J*_7,8_ = 8.1 Hz, 7-H), 7.80 (1H, t, *J*_7,8_ = *J*_8,9_ = 8.1 Hz, 8-H), 7.84 (1H, br d, *J*_6,7_ = 8.1 Hz, 6-H), 8.0 (1H, br d, *J*_8,9_ = 8.1 Hz, 9-H). Anal. calcd. C_20_H_18_N_6_O: C, 67.02; H, 5.06; N, 23.45. Found: C, 67.36; H, 5.35; N, 23.62.

#### 7-Methoxy-2-(4-phenylpiperazin-1-yl)benzo[g]pteridin-4(3H)-one (10p)

Yield (2.60 g, 67%, DMF); m.p. 282–284 °C; UV (EtOH): *λ*_max_/nm (log *ε*/dm^3^ mol^−1^ cm^−1^): 222 sh (4.51), 248 sh (4.42), 276 (4.65), 332 (3.64), 448 (3.93); IR (*ν*_max_/cm^−1^): 3432 (OH tautomer of C═O); ^1^H NMR [(CD_3_)_2_SO]: *δ* 3.11–3.20 (4H, m, 2′ and 6′-NCH_2_), 3.32 (1H, br s, 4-OH, exchangeable with D_2_O), 3.93–4.20 (4H, m, 3′ and 5′-NCH_2_), 3.94 (3H, s, 7-OMe), 6.80 (1H, dt, *J*_3″,4″_ = *J*_4″,5″_ = 5.1 Hz, *J*_2″,4″_ = *J*_4″,6″_ = 2.1 Hz, Ph-*p*H), 6.95–7.10 (2H,m, Ph-*o*H), 7.23–7.30 (2H, m, Ph-*m*H), 7.40–7.45 (2H, m, 6 and 8-H), 7.79 (1H, d, 9-H). Anal. calcd. C_21_H_20_N_6_O_2_: C, 64.94; H, 5.19; N, 21.64. Found: C, 64.77; H, 5.52; N, 21.78.

#### 9-Methyl-2-(4-phenylpiperazin-1-yl)benzo[g]pteridin-4(3H)-one (10q)

Yield (3.0 g, 81%, DMF); m.p. 285–288 °C; UV (EtOH): *λ*_max_/nm (log *ε*/dm^3^ mol^−1^ cm^−1^): 224 sh (3.88), 252 (4.53), 270 (4.5), 286 (4.45), 346 (3.81), 434 (3.9); IR (*ν*_max_/cm^−1^): 3436 (OH tautomer of C═O); ^1^H NMR [(CD_3_)_2_SO]: *δ* 2.50 (1H, s, 9-Me) 3.17–3.19 (4H, m, 2′ and 6′-NCH_2_), 3.39 (1H, br s, 4-OH, exchangeable with D_2_O); 3.92–3.96 (4H, m, 3′ and 5′-NCH_2_), 6.80 (1H, br t, *J*_3″,4″_ = *J*_4″,5″_ = 6.7 Hz, Ph-*p*H), 6.91–6.99 (2H,m, Ph-*o*H), 7.19–7.22 (2H, m, Ph-*m*H), 7.27 (1H, t, *J*_6,7_ = *J*_7,8_ = 7.7 Hz, 7-H), 7.60 (1H, br d*, J*_7,8_ = 7.7 Hz, 8-H), 7.8 (1H, br d *J*_6,7_ = 7.7 Hz, 6-H). Anal. calcd. C_21_H_20_N_6_O: C, 67.73; H, 5.41; N, 22.57. Found: C, 67.89; H, 5.80; N, 22.85.

### General procedure for the preparation of 2-(methylthio)-alloxazines (11a–c)

A mixture of 2-methylthio-4-oxo-3,4-dihydrobenzo[*g*]pteridin-5-oxide analogue (**9**, 1.9 mmol) and sodium dithionite (0.67 g, 3.8 mmol) in 20 mL of H_2_O was stirred at r.t. for 12 h. The yellow precipitate was obtained by filtration, washed thoroughly with water, and recrystallised from DMF, to afford the pure products as yellow crystals in 65–67% yields.

#### 2-(Methylthio)benzo[g]pteridin-4(3H)-one (11a)

Yield (0.30 g, 65%, DMF); m.p. 270–272 °C; UV (EtOH): *λ*_max_/nm (log *ε*/dm^3^ mol^−1^ cm^−1^): 220 (4.17) 248 sh (4.26), 270 sh (4.43), 352 (3.8), 438 (3.66); IR (*ν*_max_/cm^−1^): 3172 (NH), 1686 (C═O); ^1^H NMR [(CD_3_)_2_SO]: *δ* 2.59 (3H, s, 2-SMe), 7.38–7.75 (4H, m, 6,7,8,9-H), 12.80 (1H, br s, 3-NH, exchangeable with D_2_O). Anal. calcd. C_11_H_8_N_4_OS: C, 54.09; H, 3.30; N, 22.94. Found: C, 54.37; H, 3.67; N, 22.65. MS; EI, C_11_H_8_N_4_OS *m/z* (R.A. %): 244 (M^+^, 100%), 197 (12%), 186 (18%), 169 (54%).

#### 7-Methyl-2-(methylthio)benzo[g]pteridin-4(3H)-one (11b)

Yield (0.32 g, 65%, DMF); m.p. >300 °C; UV (EtOH): *λ*_max_/nm (log *ε*/dm^3^ mol^−1^ cm^−1^): 216 (4.1) 244 sh (4.09), 274 sh (4.45), 350 (3.69), 444 (3.66); IR (*ν*_max_/cm^−1^): 3175 (NH), 1687 (C═O); ^1^H NMR [(CD_3_)_2_SO]: *δ* 2.52 (3H, s, 7-Me), 2.56 (3H, s, 2-SMe), 7.76–7.82 (1H, m, 8-H), 7.88–7.96 (1H, m, 9-H), 8.14 (1H, s, 6-H), 12.79 (1H, br s, 3-NH, exchangeable with D_2_O). Anal. calcd. C_12_H_10_N_4_OS: C, 55.80; H, 3.90; N, 21.69. Found: C, 56.00; H, 4.30; N, 21.53. MS; EI, C_12_H_10_N_4_OS *m/z* (R.A. %): 258 (M^+^, 100%), 211 (15%), 183 (51%).

#### 7-methoxy-2-(methylthio)benzo[g]pteridin-4(3H)-one (11c)

Yield (0.35 g, 67%, DMF); m.p. >300 °C; UV (EtOH): *λ*_max_/nm (log *ε*/dm^3^ mol^−1^ cm^−1^): 216 (4.06) 242 sh (3.87), 286 sh (4.47), 348 (3.54), 456 (3.67); IR (*ν*_max_/cm^−1^): 3172 (NH), 1684 (C═O); ^1^H NMR [(CD_3_)_2_SO]: *δ* 2.58 (3H, s, 2-SMe), 3.95 (3H, s, 7-OMe), 7.58 (1H, d, *J_8,9_* = 9.3, 8-H), 7.65 (1H, s, 6-H), 7.91 (1H, d, *J_8,9_* = 9.3 Hz 9-H), 12.77 (1H, br s, 3-NH, exchangeable with D_2_O). Anal. calcd. C_12_H_10_N_4_O_2_S: C, 52.54; H, 3.67; N, 20.43. Found: C, 52.14; H, 4.00; N, 20.15. MS; EI, C_12_H_10_N_4_O_2_S *m/z* (R.A. %): 274 (M^+^, 73%), 273 (97%), 199 (100%).

### General procedure for the preparation of 3-alkyl-2-deoxo-2-methylth-ioalloxazin-5-oxides (3-alkyl-2-methylthio-4-oxo-3,4-dihydrobenzo[g]pteridin-5-oxides) (12a–c)

A solution containing 2-methylthio-4-oxo-3,4-dihydrobenzo[*g*]pteridine-5-oxide (**9**, 5.0 mmol) was mixed with an appropriate alkyl iodide (0.02 mol) in dried DMF (30 mL) along with anhydrous KCO_3_ (2.76 g, 0.02 mol). The mixture was stirred at r.t. for 10 h and the reaction was monitored by TLC. Once the reaction was complete, the solid KCO_3_ was filtered off and rinsed with dichloromethane. The filtrate was then concentrated under vacuum. The resulting residue was extracted with dichloromethane (3 × 50 mL), dried using anhydrous magnesium sulphate, and filtered. The solvent was then removed under vacuum to yield the solid product. The crude product was dried and subjected to crystallisation from DMF, resulting in the desired product as yellow crystals in 66–76% yields.

#### 3-Ethyl-2-deoxo-2-methylthioalloxazin-5-oxide (12a)

Yield (1.10 g, 76%, DMF–H_2_O); m.p. 204–206 °C; UV (EtOH): *λ*_max_/nm (log *ε*/dm^3^ mol^−1^ cm^−1^): (4.53), 272 (4.51), 298 (4.42), 338 sh (3.99), 402 (3.84), 442 sh (3.72); IR (*ν*_max_/cm^−1^): 1697 (C═O); ^1^H NMR [(CD_3_)_2_SO]: *δ* 1.27 (3H, t, *J* = 6.9, 3-NCH_2_-*CH_3_*), 2.66 (3H, s, 2-SMe), 4.03 (2H, q, *J* = 6.9 Hz, 3-N*CH_2_*-CH_3_), 7.71 (1H, br t, *J_6,7_* = *J_7,8_* = 7.8 Hz, 7-H), 7.91 (1H, br t, *J_7,8_* = *J_8,9_* = 7.8 Hz, 8-H), 8.00 (1H, br d *J_8,9_* = 7.8 Hz, 9-H), 8.36 (1H, br d *J_6,7_* = 7.8 Hz, 6-H). Anal. calcd. C_13_H_12_N_4_O_2_S: C, 54.15; H, 4.20; N, 19.43. Found: C, 54.26; H, 4.53; N, 19.59. MS; EI, C_13_H_12_N_4_O_2_S *m/z* (R.A. %): 288 (M^+^, 35%), 272 (24%), 257 (39%), 243 (16%), 229 (100%), 214 (18%).

#### 3-Methyl-7-methoxy-2-deoxo-2-methylthioalloxazin-5-oxide (12b)

Yield (1.15 g, 76%, DMF); m.p. 262–246 °C; UV (EtOH): *λ*_max_/nm (log *ε*/dm^3^ mol^−1^ cm^−1^): 216 (4.11), 292 (4.52), 340 sh (3.76), 436 (3.68), 454 sh (3.63); IR (*ν*_max_/cm^−1^): 1690 (C═O); ^1^H NMR [(CD_3_)_2_SO]: *δ* 2.64 (3H, s, 2-SMe), 3.44 (3H, s, 3-NMe), 3.96 (3H, s, 7-OMe), 7.60 (1H, dd, *J_8,9_* = 9.3, *J_6,8_* = 2.5 Hz, 8-H), 7.66 (1H, d, *J_6,8_* = 2.5 Hz, 6-H), 7.94 (1H, d, *J_8,9_* = 9.3 Hz 9-H). Anal. calcd. C_13_H_12_N_4_O_3_S: C, 51.31; H, 3.97; N, 18.41. Found: C, 51.61; H, 4.07; N, 18.50.

#### 3-Methyl-9-methyl-2-deoxo-2-methylthioalloxazin-5-oxide (12c)

Yield (1.10 g, 76%, DMF); m.p. 238–240 °C; UV (EtOH): *λ*_max_/nm (log *ε*/dm^3^ mol^−1^ cm^−1^): 222 (3.95), 274 (4.49), 304 (4.48), 346 sh (3.64), 424 (3.66), 452 sh (3.44); IR (*ν*_max_/cm^−1^): 1690 (C═O); ^1^H NMR [(CD_3_)_2_SO]: *δ* 2.67 (3H, s, 9-Me), 2.69 (3H, 2-SMe), 3.46 (3H, s, 3-NMe), 7.68 (1H, t, *J_6,7_* = *J_7,8_* = 7.5 Hz,7-H), 7.78 (1H, br d, *J_7,8_* = 7.5 Hz, 8-H), 8.13 (1H, br d, *J_6,7_* = 7.5 Hz, 6-H). Anal. calcd. C_13_H_12_N_4_O_2_S: C, 54.15; H, 4.20; N, 19.43. Found: C, 54.21; H, 4.43; N, 19.31.

#### 3-Ethyl-7-methyl-2-deoxo-2-methylthioalloxazin-5-oxide (12d)

Yield (1.0 g, 66%, DMF); m.p. 204–206 °C; UV (EtOH): *λ*_max_/nm (log *ε*/dm^3^ mol^−1^ cm^−1^): 220 (4.1), 280 (4.5), 300 (4.45), 336 sh (3.8), 426 (3.69), 450 sh (3.55); IR (*ν*_max_/cm^−1^): 1683 (C═O); ^1^H NMR [(CD_3_)_2_SO]: *δ* 1.28 (3H, t, *J* = 6.9, 3-N-CH_2_-*CH_3_*), 2.55 (3H, s, 7-Me), 2.67 (3H, s, 2-SMe), 4.05 (2H, q, *J* = 6.9 Hz, 3-N*CH_2_*-CH_3_), 7.79 (1H, dd, *J_8.9_* = 7.9 Hz, *J_6,8_* = 1.4 Hz, 8-H), 7.92 (1H, d, *J_8.9_* = 7.9 Hz, 9-H), 8.18 (1H, s, 6-H). Anal. calcd. C_14_H_14_N_4_O_2_S: C, 55.61; H, 4.67; N, 18.53. Found: C, 55.45; H, 5.09; N, 18.62.

#### 3-Ethyl-9-methyl-2-deoxo-2-methylthioalloxazin-5-oxide (12e)

Yield (1.0 g, 66%, DMF); m.p. 200–202 °C; UV (EtOH): *λ*_max_/nm (log *ε*/dm^3^ mol^−1^ cm^−1^): 222 (3.95), 274 (4.48), 304 (4.51), 344 sh (3.68), 426 (3.67), 450 sh (3.5); IR (*ν*_max_/cm^−1^): 1690 (C═O); ^1^H NMR [(CD_3_)_2_SO]: *δ* 1.28 (3H, t, *J* = 6.9, 3-NCH_2_-*CH_3_*), 2.67 (3H, s, 9-Me), 2.70 (3H, s, 2-SMe), 4.07 (2H, q, *J* = 6.9 Hz, 3-N*CH_2_*-CH_3_), 7.68 (1H, t, *J_6,7_* = *J_7,8_* = 7.2 Hz,7-H), 7.78 (1H, d, *J_7,8_* = 7.2 Hz, 8-H), 8.21 (1H, d, *J_6,7_* = 7.2 Hz, 6-H). Anal. calcd. C_14_H_14_N_4_O_2_S: C, 55.61; H, 4.67; N, 18.53. Found: C, 55.82; H, 5.30; N, 18.61.

### General procedure for the preparation of 2,4-dioxo-1,2,3,4-tetrahydrobenzo[g]pteridin-5-oxides (13a–d)

To 2-methylthio-4-oxo-3,4-dihydrobenzo[*g*]pteridine-5-oxide analogue (**9**, 0.01 mol) was added 5 N HCl (250 mL). Then, the mixture was heated under reflux for 15 h and the resulting yellow crystals were filtered off. The filtrate was concentrated in vacuum to remove the excess acid, and the combined first crop crystals and residue of the filtrate were taken with water and neutralised with aqueous ammonia to pH 7. The precipitated powdery product was filtered, washed well with water, dried, and recrystallised from glacial AcOH to afford yellow crystals in 77–78% yields.

#### 2,4-Dioxo-1,2,3,4-tetrahydrobenzo[g]pteridin-5-oxide (13a)

Yield (1.80 g, 78%, glacial acetic acid); m.p. >300 °C, (decomp., from glacial acetic acid); UV (EtOH): *λ*_max_/nm (log *ε*/dm^3^ mol^−1^ cm^−1^): 214 (4.26), 242 (4.29), 258 (4.26), 322 (3.67), 380 (3.59); IR (*ν*_max_/cm^−1^): 3240, 3099 (NH), 1720, 1665 (C═O); ^1^H NMR [(CD_3_)_2_SO]: *δ* 7.63–7.68 (1H, m, 7-H), 7.75–7.80 (1H, m, 8H), 8.15 (1H, br d, *J_8,9_* = 8.5 Hz, 9-H), 8.32 (1H, br d, *J_6,7_* = 8.5 Hz, 6-H), 11.73 (1H, br s,1-NH, exchangeable with D_2_O), 11.84 (1H, br s, 3-NH, exchangeable with D_2_O). Anal. calcd. C_10_H_6_N_4_O_3_: C, 52.18; H, 2.63; N, 24.34. Found: C, 52.37; H, 2.96; N, 24.46.

#### 2,4-Dioxo-7-methoxy-1,2,3,4-tetrahydrobenzo[g]pteridin-5-oxide (13b)

Yield (2.0 g, 77%, glacial acetic acid); m.p. >300 °C; UV (EtOH): *λ*_max_/nm (log *ε*/dm^3^ mol^−1^ cm^−1^): 216 (4.21), 242 sh (4.12), 268 (4.45), 318 (3.71), 424 (3.67); IR (*ν*_max_/cm^−1^): 3252, 3093 (NH), 1699, 1630 (C═O); ^1^H NMR [(CD_3_)_2_SO]: *δ* 3.98 (3H, s,7-OMe), 7.59–7.65 (2H, m, 6 and 8-H), 7.81–7.95 (1H, m, 9-H), 11.60 (2H, br s, 1-NH and 3-NH, exchangeable with D_2_O). Anal. calcd. C_11_H_8_N_4_O_4_: C, 50.77; H, 3.10; N, 21.53. Found: C, 51.00; H, 3.10; N, 21.47.

#### 2,4-Dioxo-7-methyl-1,2,3,4-tetrahydrobenzo[g]pteridin-5-oxide (13c)

Yield (1.90 g, 78%, glacial acetic acid); m.p. >300 °C; UV (EtOH): *λ*_max_/nm (log *ε*/dm^3^ mol^−1^ cm^−1^): 216 (4.12), 240 sh (4.14), 268 (4.32), 334 (3.3), 404 (3.51); IR (*ν*_max_/cm^−1^): 3186, 3081 (NH), 1702, 1620 (C═O); ^1^H NMR [(CD_3_)_2_SO]: *δ* 2.54 (3H, s, 7-Me), 7.70 (1H, br d, *J_8,9_* = 8.7 Hz, 8-H), 7.92 (1H, br d, *J_8,9_* = 8.7 Hz, 9-H), 8.12 (1H, br s, 6-H), 11.39 (1H, br s, 1-NH, exchangeable with D_2_O), 11.69 (1H, br s, 3-NH exchangeable with D_2_O). Anal. calcd. C_11_H_8_N_4_O_3_: C, 54.10; H, 3.30; N, 22.94. Found: C, 54.33; H, 3.60; N, 23.09.

#### 2,4-Dioxo-9-methyl-1,2,3,4-tetrahydrobenzo[g]pteridin-5-oxide (13d)

Yield (1.90 g, 78%, glacial acetic acid); m.p. >300 °C; UV (EtOH): *λ*_max_/nm (log *ε*/dm^3^ mol^−1^ cm^−1^): 216 (3.96), 242 sh (4.18), 272 (4.42), 328 (3.57), 408 (3.49); IR (*ν*_max_/cm^−1^): 3194, 3107 (NH), 1737, 1696 (C═O); ^1^H NMR [(CD_3_)_2_SO]: *δ* 2.61 (3H, s, 9-Me), 7.51 (1H, t, *J_6,7_* = *J_7,8_* = 7.8 Hz,7-H), 7.92 (1H, br d, *J_7,8_* = 7.8 Hz, 8-H), 8.15 (1H, br d, *J_6,7_* = 7.8 Hz, 6-H), 11.39 (1H, br s, 1-NH exchangeable with D_2_O), 11.55 (1H, br s, 3-NH exchangeable with D_2_O). Anal. calcd. C_11_H_8_N_4_O_3_ 0.1H_2_O: C, 53.70; H, 3.36; N, 22.77. Found: C, 53. 52; H, 3.49; N, 22.52.

### General procedure for the preparation of 2-amino-benzo[g]pteridin-4(3-H)-ones (14a,b)

A mixture of 2-methylthio-4-oxo-3,4-dihydrobenzo[g]pteridine-5-oxide analogue (**9**, 4.0 mmol) and ammonium acetate (0.2 mol) was fused under stirring at 160–165 °C for 0.5–3.0 h. The reaction mixture was cooled, diluted with water (15 mL), neutralised with aqueous ammonia (pH 7), and cooled in the refrigerator overnight. The resulting brown crystals were collected by filtration, dried, and recrystallised from DMF to give the corresponding products as brown needles in 69–74% yields.

#### 2 -amino-9-methyl-benzo[g]pteridin-4(3H)-one (14a)

Yield (0.67 g, 74%, DMF); m.p. >300 °C; UV (EtOH): *λ*_max_/nm (log *ε*/dm^3^ mol^−1^ cm^−1^): 214 (4.28), 258 (4.37), 280 sh (4.21), 340 (3.72), 400 (3.71); IR (*ν*_max_/cm^−1^): 3269, 3148 (NH), 1698 (C═O); ^1^H NMR [(CD_3_)_2_SO]: *δ* 2.63 (3H, s, 9-Me), 7.02 (2H, br s, 2-NH_2_, exchangeable with D_2_O), 7.55 (1H, t, *J_6,7_* = *J_7,8_* = 6.7 Hz, 7-H), 7.67 (1H, br d, *J_7,8_* = 6.7 Hz, 8-H), 7.91 (1H, br d, *J_6,7_* = *6.7* Hz, 6-H), 11.60 (1H, br s, 3-NH, exchangeable with D_2_O). Anal. calcd. C_11_H_9_N_5_O: C, 58.14; H, 3.99; N, 30.82. Found: C, 57.90; H, 4.16; N, 30.52.

#### 2-Amino-7-methoxybenzo[g]pteridin-4(3H)-one (14b)

Yield (0.67 g, 69%, DMF); m.p. >300 °C; UV (EtOH): *λ*_max_/nm (log *ε*/dm^3^ mol^−1^ cm^−1^): 220 (4.27), 268 (4.36), 326 (3.67), 424 (3.8); IR (*ν*_max_/cm^−1^): 3256, 3140 (NH), 1698 (C═O); ^1^H NMR [(CD_3_)_2_SO]: *δ* 3.96 (3H, s, 7-OMe), 6.80 (2H, br s, 2-NH_2_, exchangeable with D_2_O), 7.51 (1H, dd, *J_8,9_* = 9.6 Hz, *J_6,8_* = 2.7 Hz, 8-H), 7.80 (1H, d, *J_8,9_* = 9.6 Hz, 9-H), 7.95 (1H, br s, 6-H), 11.40 (1H, br s, 3-NH, exchangeable with D_2_O). Anal. calcd. C_11_H_9_N_5_O_2_: C, 54.32; H, 3.73; N, 28.79. Found: C, 54.13; H, 4.08; N, 28.48.

### General procedure for the preparation of 6-(N-anilino) pyrimidin-4(3H)-ones (15a–c)

A stirring solution of Raney nickel catalyst (8.0 g, 0.14 mol) in absolute EtOH (100 mL) was mixed with 2,3-dihydro-6-(*N*-anilino)-2-thioxopyrimidin-4(1*H*)-ones (**7**, 0.004 mol). The mixture was then refluxed for a 0.5–1.5 h. The mixture was filtered while still hot to eliminate the catalyst, which was thoroughly washed with boiling EtOH (4 × 20 mL). The combined filtrate and washings were then evaporated to dryness. The resulting residue underwent recrystallisation from water, yielding colourless crystals in 58–72% yields.

#### 6-(N-Anilino) pyrimidin-4(3H)-one (15a)

Yield (0.54 g, 72%, H_2_O); m.p. 200 °C; UV (EtOH): *λ*_max_/nm (log *ε*/dm^3^ mol^−1^ cm^−1^): 250 (4.17), 288 (4.18); IR (*ν*_max_/cm^−1^): 3302, 3213 (NH), 1671 (C═O); ^1^H NMR [(CD_3_)_2_SO]: *δ* 5.09 (1H, s, 5-H), 7.20 (1H, br t, *J*_3′,4′_ = *J*_4′,5′_ = 8.1 Hz, Ph-*p*H), 7.50 (2H, br t, *J*_2′,3′_ = *J*_3′,4′_ = *J*_4′,5′_ = *J*_5′,6′_ = 8.1 Hz, Ph*-m*H), 7.54 (2H, br d, *J*_2′,3′_ = *J*_5′,6′_ = 8.1, Ph*-o*H), 7.97 (1H, s, 2-H), 8.79 (1H, s, 6-NH, exchangeable with D_2_O), 10.15 (1H, br s, 3-NH, exchangeable with D_2_O). Anal. calcd. C_10_H_9_N_3_O: C, 64.16; H, 4.85; N, 22.45. Found: C, 64.35; H, 5.20; N, 22.62. MS; EI, C_13_H_12_N_4_O_2_S *m/z* (R.A. %): 187 (M^+^, 7%), 186 (20%), 158 (47%), 144 (100%).

#### 6-(o-Tolylamino)pyrimidin-4(3H)-one (15b)

Yield (0.56 g, 70%, H_2_O); m.p. 152–154 °C; UV (EtOH): *λ*_max_/nm (log *ε*/dm^3^ mol^−1^ cm^−1^): 242 (4.12), 276 (3.93); IR (*ν*_max_/cm^−1^): 3405, 3210 (NH), 1637 (C═O); ^1^H NMR [(CD_3_)_2_SO]: *δ* 2.18 (3H, s, Ph-*o*Me), 4.83 (1H, s, 5-H), 7.11–7.28 (4H, m, Ph*-o,m, p*H), 7.80 (1H, s, 2-H), 8.53 (1H, s, 6-NH, exchangeable with D_2_O), 11.60 (1H, br s, 3-NH, exchangeable with D_2_O). Anal. calcd. C_11_H_11_N_3_O.0.4H_2_O: C, 63.39; H, 5.71; N, 20.16. Found: C, 63.13; H, 6.08; N, 19.85.

#### 6-(p-Anisidino) pyrimidin-4(3H)-one (15c)

Yield (0.50 g, 58%, H_2_O); m.p. 268–270 °C; UV (EtOH): *λ*_max_/nm (log *ε*/dm^3^ mol^−1^ cm^−1^): 248 (4.32), 286 (4.23); IR (*ν*_max_/cm^−1^): 3407, 3220 (NH), 1638 (C═O); ^1^H NMR [(CD_3_)_2_SO]: *δ* 3.73 (3H, s, Ph-*p*OMe), 5.20 (1H, s, 5-H), 6.90 (2H, br d, *J*_2′,3′_ = *J*_5′,6′_ = 8.51, Ph*-o*H), 7.20 (2H, br d, *J*_2′,3′_ = *J*_5′,6′_ = 8.5 Hz, Ph*-m*H), 7.90 (1H, s, 2-H), 8.81 (1H, s, 6-NH, exchangeable with D_2_O), 11.60 (1H, br s, 3-NH, exchangeable with D_2_O). Anal. calcd. C_11_H_11_N_3_O_2_: C, 60.82; H, 5.10; N, 19.34. Found: C, 61.14; H, 5.36; N, 19.52.

### General procedure for the preparation of 4-oxo-3,4-dihydrobenzo[g]pteridin-5-oxide (16a–c)

To a solution of 6-(*N*-anilino) pyrimidin-4(3*H*)-ones (**15**, 0.002 mol) in AcOH (5–15 mL) at 10–15 °C was added NaNO_2_ (0.008–0.01 mol) portion wise, and the mixture was stirred at r.t. with occasional warming to enhance cyclisation of the nitroso intermediate for 2–5 h. The precipitation was filtered and washed with water. The filtrate was concentrated in vacuum and the residue was diluted with excess water or neutralised with aqueous ammonia (pH 7) to afford the second crop. The dried solid was recrystallised from DMF to afford the corresponding products as yellow to orange crystals in 70–77% yields.

#### 4-Oxo-3,4 dihydrobenzo[g]pteridin-5-oxide (16a)

Yield (0.30 g, 70%, DMF); m.p. >300 °C; UV (EtOH): *λ*_max_/nm (log *ε*/dm^3^ mol^−1^ cm^−1^): 256 (4.33), 266 (4.35), 286 (4.00), 380 (3.68); IR (*ν*_max_/cm^−1^): 3220 (NH), 1700 (C═O); ^1^H NMR [(CD_3_)_2_SO]: *δ* 7.60 (1H, dt, *J*_6,7_ = *J*_7,8_ = 7.5 Hz, *J*_7,9_ = 1.2 Hz, 7-H), 7.80 (1H, dt, *J*_7,8_ = *J*_8,9_ = 7.5 Hz, *J*_6,8_ = 1.2 Hz, 8-H), 7.84 (1H, dd, *J*_6,8_ = 1.2 Hz*, J*_6,7_ = 7.5 Hz, 6-H), 8.0 (1H, dd, *J*_8,9_ = 7.5 Hz, *J*_7,9_ = 1.2 Hz, 9-H), 8.26 (1H, s, 2-H), 12.47 (1H, s, 3-NH, exchangeable with D_2_O). Anal. calcd. C_10_H_6_N_4_O_2_: C, 56.08; H, 2.82; N, 26.16. Found: C, 55.75; H, 3.11; N, 26.32.

#### 9-Methyl-4-oxo-3, 4-dihydrobenzo[g]pteridin-5-oxide (16b)

Yield (0.35 g, 77%, DMF); m.p. >300 °C; UV (EtOH): *λ*_max_/nm (log *ε*/dm^3^ mol^−1^ cm^−1^): 266 (4.39), 290 (4.00), 342 (3.43), 430 (3.53); IR (*ν*_max_/cm^−1^): 3133 (NH), 1705 (C═O); ^1^H NMR [(CD_3_)_2_SO]: *δ* 2.69 (3H, s, 9-Me), 7.67 (1H, t, *J*_6,7_ = *J*_7,8_ = 7.7 Hz, 7-H), 7.82 (1H, br d, *J*_7,8_ = 7.7 Hz,8-H), 8.22 (1H, br d *J_6,7_* = 7.7 Hz, 6-H), 8.25 (1H, s, 2-H), 12.47 (1H, s, 3-NH, exchangeable with D_2_O). Anal. calcd. C_11_H_8_N_4_O_2_: C, 57.89; H, 3.53; N, 24.55. Found: C, 58.15; H, 3.91; N, 24.84.

#### 7-Methoxy-4-oxo-3,4-dihydrobenzo[g]pteridin-5-oxide (16c)

Yield (0.35 g, 72%, DMF); m.p. >300 °C; UV (EtOH): *λ*_max_/nm (log *ε*/dm^3^ mol^−1^ cm^−1^): 216 (4.17), 230 (3.97), 282 (4.36), 418 (3.73); IR (*ν*_max_/cm^−1^): 3250 (NH), 1702 (C═O); ^1^H NMR [(CD_3_)_2_SO]: *δ* ^1^H NMR [(CD_3_)_2_SO]: *δ* 3.98 (3H, s, 7-OMe), 7.62–7.72 (3H, m, 6,8 and 9-H), 8.21 (1H, s, 2-H), 12.51 (1H, s, 3-NH, exchangeable with D_2_O). Anal. calcd. C_11_H_8_N_4_O_3._0.2H_2_O: C, 53.31; H, 3.42; N, 22.61. Found: C, 53.15; H, 3.59; N, 22.42.

### Growth inhibitory activities of test compounds against human tumour cell lines

This protocol adheres to the methodology outlined in our earlier publication^51^, where the anti-proliferative effects of the synthesised compounds on human tumour cell lines were assessed through the MTT assay. AraC, a commercially available product from Yamasa Corporation (Choshi, Japan), and 3-(3,4-dimethylthiazol-2-yl)-2,5-diphenyltetrazolium bromide (MTT) obtained from Sigma (St. Louis, MO) were utilised in this study. Two human tumour cell lines, CCRF-HSB-2 (human T-cell acute lymphoblastoid leukaemia) and KB (human oral epidermoid carcinoma), were obtained from the National Institute of Biomedical Innovation, Health, and Nutrition (NIBIOHN)-JCRB Cell Bank (Japanese Collection of Research Bioresources Cell Bank) (Osaka, Japan) with the reference numbers: JCRB0031 and JCRB9027, respectively. These cells were cultured separately in RPMI 1640 medium supplemented with 10% heat-inactivated FBS and 60 µg/mL of kanamycin. The modified MTT assay, based on the method described by Mosmann^34^ and further modified^35^ was used to evaluate the inhibitory effects of the experimental compounds on cell growth *in vitro*. In brief, cells (CCRF-HSB-2 or KB) were seeded into individual wells of a 96-well microplate, with the compound solution added concurrently to triplicate wells before adjusting the final volume to 100 µL. The microplate was then incubated at 37 °C for 72 h in a humidified atmosphere containing 5% CO_2_. After the incubation period, MTT solution (5 mg/mL in phosphate-buffered saline lacking calcium and magnesium) was added to each well and incubated for an additional 4 h at 37 °C. Subsequently, a solution consisting of 0.02 N HCl, 50% DMF, and 20% SDS was added to dissolve any MTT-formazan formed. The optical density of each well was measured at *λ*_570_ nm using a microplate reader, and the percentage inhibition of cell growth was calculated using the formula (1 − *T*/*C*) × 100, where *C* represents the mean optical density of the control group and *T* represents that of the treated group. The half-maximal inhibitory concentration (IC_50_) was determined from the dose–response curve.

### Protein kinase assays

The experimental conditions for the protein kinase assays were optimised to ensure satisfactory enzymatic activity and a high signal-to-noise ratio. A radioisotope assay format was employed to profile the evaluation of protein kinase targets, and all assays were conducted within a designated radioactive work area. The protein kinase assays were carried out at room temperature for 30 min in a final volume of 25 µL, following this assay reaction recipe: (1) 5 µL of diluted active protein kinase (∼10–50 nM final concentration in the assay); (2) 5 µL of substrate stock solution; (3) 5 µL of kinase assay buffer; (4) 5 µL of the test compound or 10% DMSO; and (5) 5 µL of 33 P-ATP (50 µM stock solution, 0.8 µCi). The assay commenced with the addition of ^33^P-ATP, and the reaction mixture was then incubated at room temperature for 30 min. Following the incubation period, the assay was halted by spotting 10 µL of the reaction mixture onto a Multiscreen phosphocellulose P81 plate. This plate underwent three washes, each lasting approximately 15 min, with a 1% phosphoric acid solution. The radioactivity on the P81 plate was measured in the presence of scintillation fluid using a Trilux scintillation counter. A blank control was established, which included all assay components except for the substrate (replaced with an equivalent volume of assay dilution buffer). The corrected activity for the protein kinase target was determined by subtracting the blank control value.

### Molecular docking study

Using ADT, we conducted the docking study of potential antitumor compounds utilising our previously published protocol^53^ to calculate the position of docked ligands and flexible residues involved in this interaction. The main task is to compare the energies of the interaction in different conformations and determine the best fit.

### Procedure^49^

AutoDock tool consists of two main programs: Auto Grid pre-calculates these grids. AutoDock performs the docking of the ligand to a set of grids describing the target protein, and working with ADT includes major three steps.

## Preparation of target protein and ligand files

### Preparation of the protein

The protein structure was retrieved from the Protein Data Bank (PDB) website at http://www.rcsb.org/ in .pdb format, a specialised format for protein structures derived from X-ray crystallography or NMR studies. These structures are represented as text files containing essential information about the molecule, such as atom counts, atom names, bond lengths, angles, dihedral angles, residue numbers, etc. Sometimes, the structure file may contain excessive or insufficient information for a specific purpose, necessitating editing. In certain applications, the inclusion of hydrogen atoms in the file is deemed unnecessary (for instance, due to the substantial increase in file size resulting from the large number of hydrogen atoms in proteins, and the simplicity of adding hydrogens later). Therefore, in this step, it is essential to reintroduce all hydrogen atoms for ADT calculations. Additionally, it is imperative to eliminate water molecules from the protein surface, as the presence of excess water molecules could obscure the protein surface from the ligand.

### Preparation of the ligand

The ligands were created using Chem3D Ultra 8.0 software (Chemical Structure Drawing Standard; Cambridge Soft Corporation, Cambridge, MA) to generate standard 3D structures in pdb format. Subsequently, they were optimised to 100 conformations using MOPAC iterations and minimised to achieve a gradient RMS of 0.10. It is advisable to ensure that the file contains hydrogen atoms before using ADT. Once the ligand is loaded, it becomes visible, enabling ADT to automatically compute Gasteiger charges (empirical atomic partial charges) and identify the type and hybridisation state of each atom. Preparation involves determining the rotatable bonds of the ligand to generate various conformers for docking.

### Preparation of the flexible residue file

The used software was unique in its consideration of the flexibility of both the ligand and the enzyme during the docking process. This means that ADT can model the positioning of flexible residues in addition to docking the ligand to the protein. To utilise this feature, we need to identify flexible residues and rotatable bonds. Flexible residues refer to amino acids in the binding site region of the protein that can change their position through conformational changes induced by ligand binding. These residues are identified through comparisons of different crystallised structures or molecular dynamic simulations. Based on the literature, the flexible residues are Glu640, Thr670, and Asp810. Rotatable bonds are utilised by the program to generate rotational isomers of amino acids and present enzyme structures with these conformers.

## Calculation of affinity maps by using a 3D grid embracing the protein and ligand

AutoGrid, a component of ADT, calculates the energy of interactions between protein and probe atoms positioned across a lattice of grid points, identifying the region of interest within the macromolecule where ligand binding is being investigated. AutoGrid generates multiple files corresponding to the number of probe atoms employed. Approximately, 30 distinct types of grid maps are produced, each illustrating interaction energies for specific atom types such as aliphatic/aromatic carbons and hydrogen bonding oxygen. The grid itself is a defined box located at the protein’s surface where ligand interaction is presumed to occur, serving as the fundamental area of study.

The generated 3D grids are of dimensions 60 × 60 × 60 Å (*x*, *y*, *z*) with a spacing of 0.375 Å, centred at coordinates 27.696, 26.657, and 39.342 Å, encompassing the active site containing the co-crystallised ligand, STI-571 (Gleevec or Imatinib), within the PTK receptor for docking inhibitors. This modelling phase involves determining the ligand’s interacting area with the enzyme, its size, and the specific types of atoms involved in the PTK enzyme interaction. The position and size of the grid box define the first two parameters, while the map type determines the third parameter. AutoGrid calculates grid parameter files for each atom type within the designated area after configuring these parameters into a single file.

### Defining the docking parameters and running the docking simulation

In the ground to work of the input files (ligand/protein) and the designing of the affinity maps were properly accomplished, AutoDock will automatically perform the docking via the newest docking algorithm (Lamarckian genetic algorithm).

### Preparation of the docking parameter file (.dpf)

After completing all files, we need to postulate for the program what specific ligand, protein, map, and flexible protein, we need to work with and also required algorithm, which, how many iterations are requisite, and so on. That information is generally kept in a .dpf.

### Running Autodock 4 and viewing the docking results

As a consequence of the calculations of AutoDock, we find the output file in our case 10 conformers of the ligand–protein complex with flexible residues and the ligand located in the binding pocket. Each structure is recorded and graded by the program via the calculated interaction energy.

## Supplementary Material

Supplementary_Information.docx

## Data Availability

All the spectroscopic characterisation data, schemes, screenings, and detailed experimental procedures are available in the Supplementary Information. All further relevant details are available upon request from the corresponding author.
