# Supplementary material for: Novel alloxazine analogues: design, synthesis, and antitumour efficacy enhanced by kinase screening, molecular docking, and ADME studies
Source: J Enzyme Inhib Med Chem. 2024 Sep 17;39(1):2398551. doi: 10.1080/14756366.2024.2398551 (PMC11409418; doi:10.1080/14756366.2024.2398551)
Supplement: Supplementary_Information.docx [file IENZ_A_2398551_SM8971.docx]

**Supplementary Information (S.I.)**

**Novel Alloxazine Analogs: Design, Synthesis, and Antitumor Efficacy Enhanced by Kinase Screening,**

**Molecular Docking, and ADME Studies**

Doaa Samaha^1,2^, Sawsan Mahmoud^1^, Mosaad S. Mohamed^3^, Rokaia S. Abdullah^4,6^, Nageh A. Abou Taleb^1^,

Tomohisa Nagamatsu^5^, Hamed I. Ali^6,*^

*^1^Pharmaceutical Chemistry Department, Faculty of Pharmacy, Helwan University, Helwan 11795, Egypt*

^2^ Institute of Chemistry, Humboldt-Universität zu Berlin, Brook-Taylor-Str. 2, 12489 Berlin, Germany

*^3^Pharmaceutical Organic Chemistry Department, Faculty of Pharmacy, Helwan University, Helwan 11795, Egypt*

*^4^Environment Division, National Institute of Oceanography and Fisheries (NIOF), Kayet Bey, El‑Anfoushy,*

*Alexandria, Egypt.*

*^5^Laboratory of Curative Creation Study for Geriatric-diseases Prevention, Faculty of Pharmacological Sciences, Sojo University, 4-22-1 Ikeda, Nishi-ku, Kumamoto, 860-0082, Japan*

*^6^Department of Pharmaceutical Sciences, Irma Lerma Rangel School of Pharmacy, Texas A&M University, College Station, Texas 77843, United States*


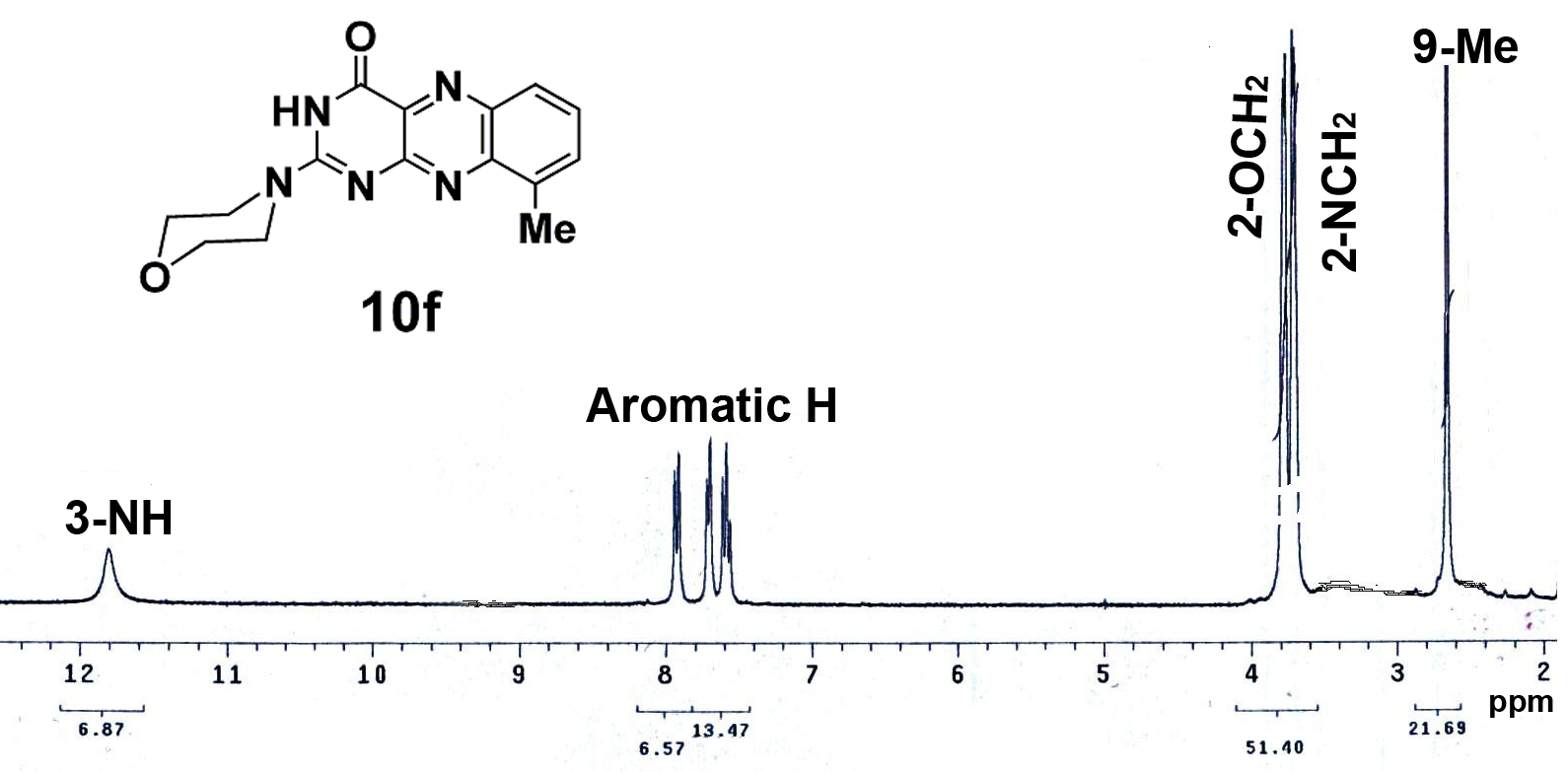


**Figure S1**. The NMR spectra of compound 9-methyl-2-(morpholin-4-yl) alloxazine (**10f**).


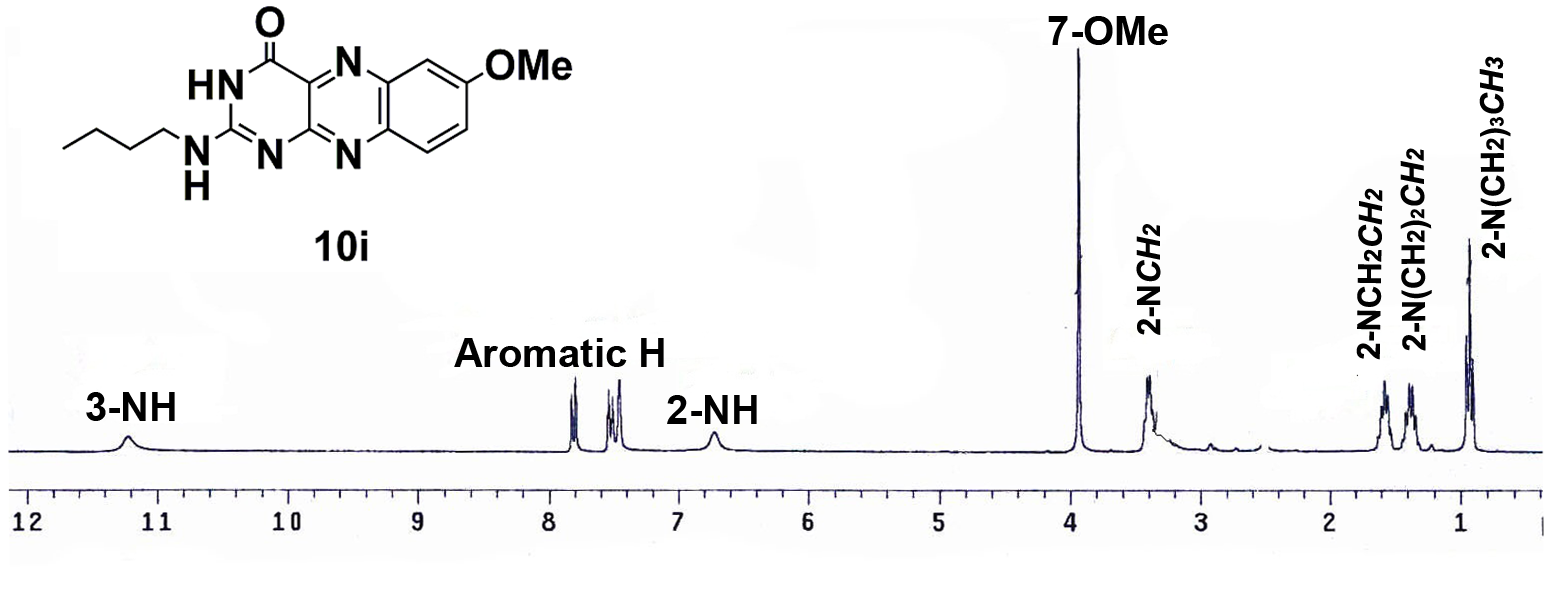


**Figure S2**. The NMR spectrum of compound 2-(butylamino)-7-methoxy-alloxazine (**10i**).


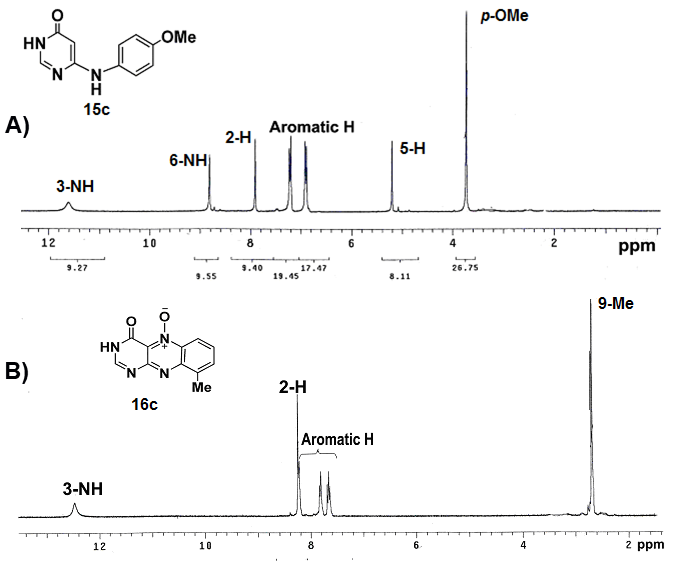


**Figure S3**. Comparative NMR spectra of compound 6-(*p*-anisidino) pyrimidin-4(3*H*)-one **(15c)** and 9-methyl-4-oxo-3,4-dihydro-alloxazine-5-oxide **(16b).**


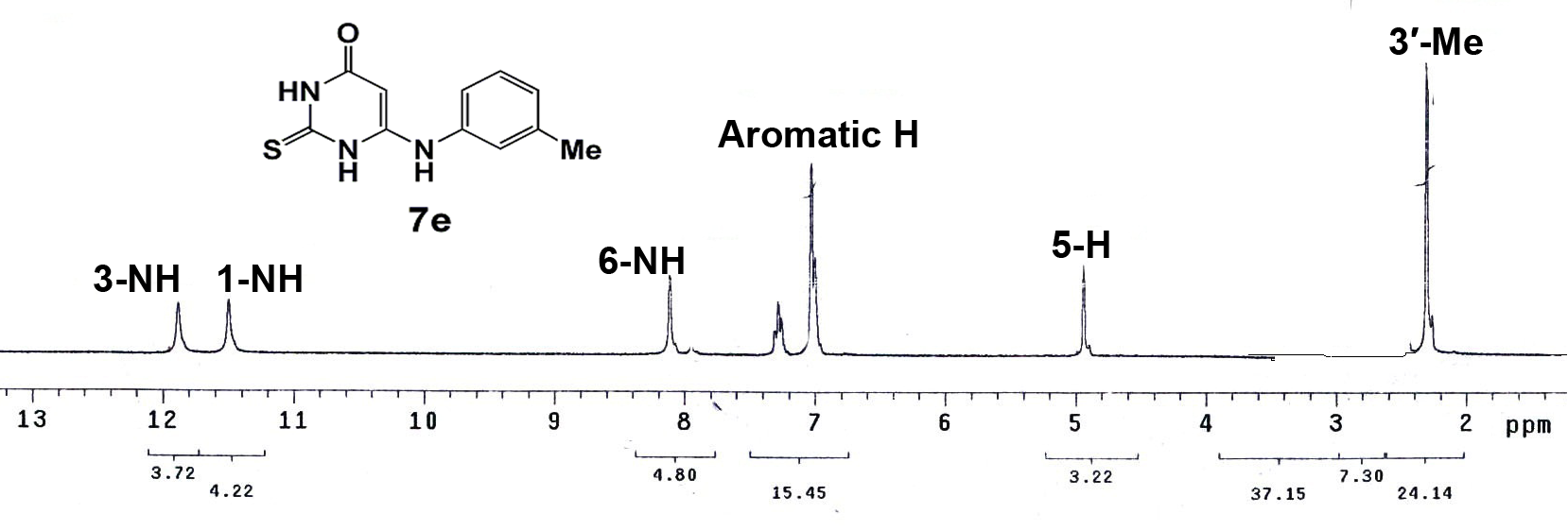


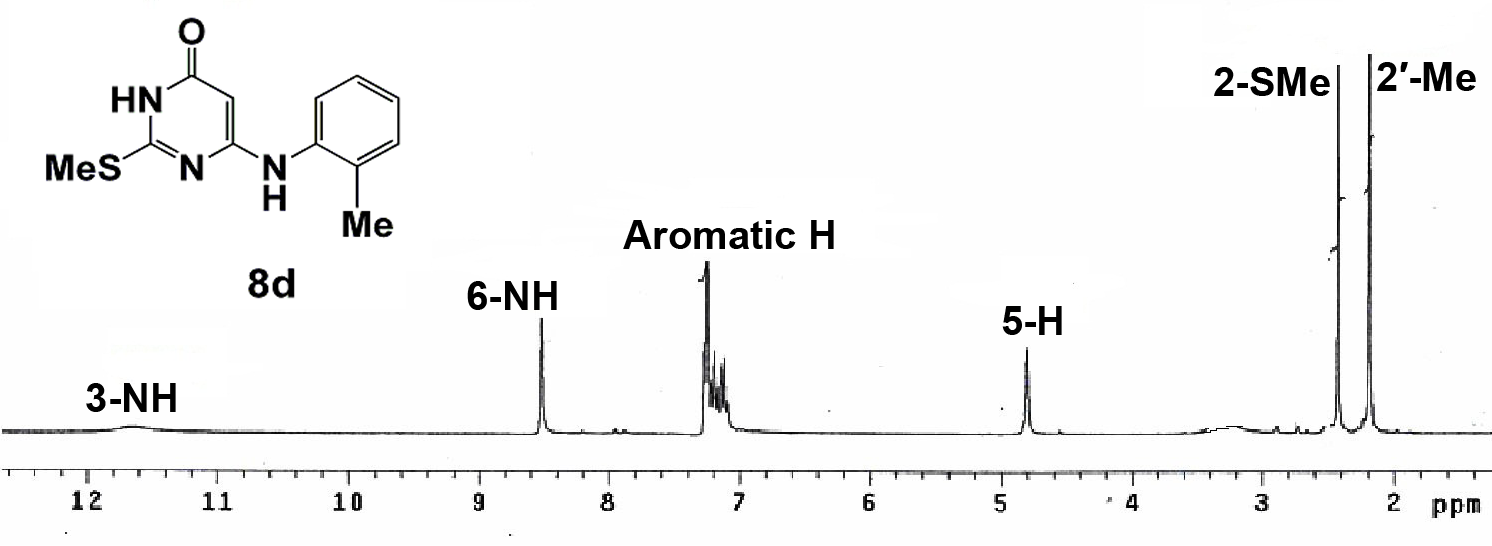


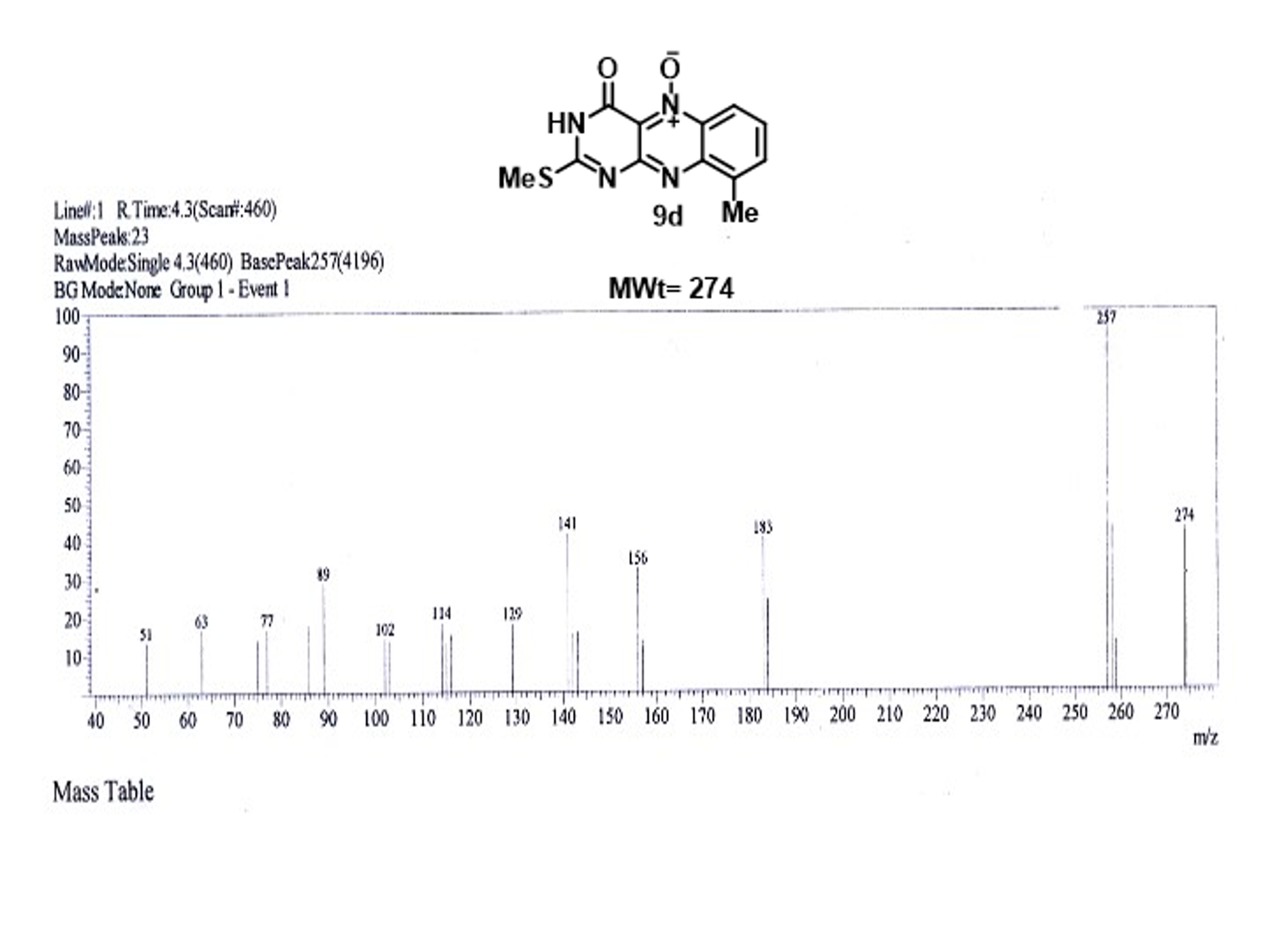


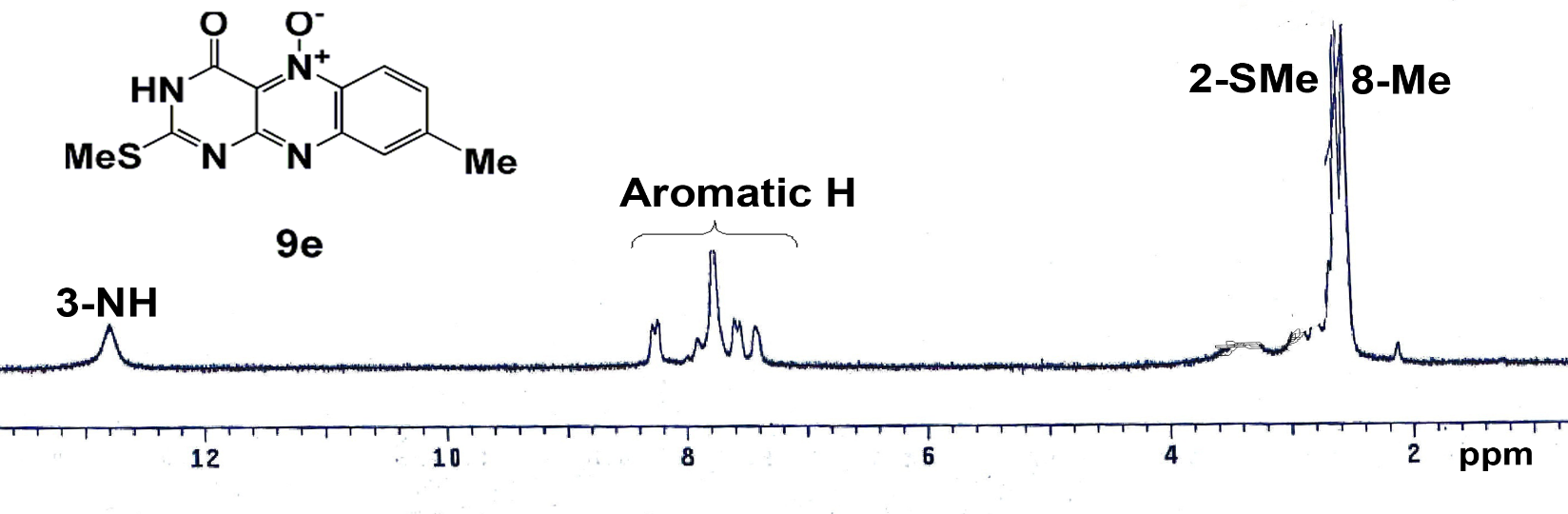


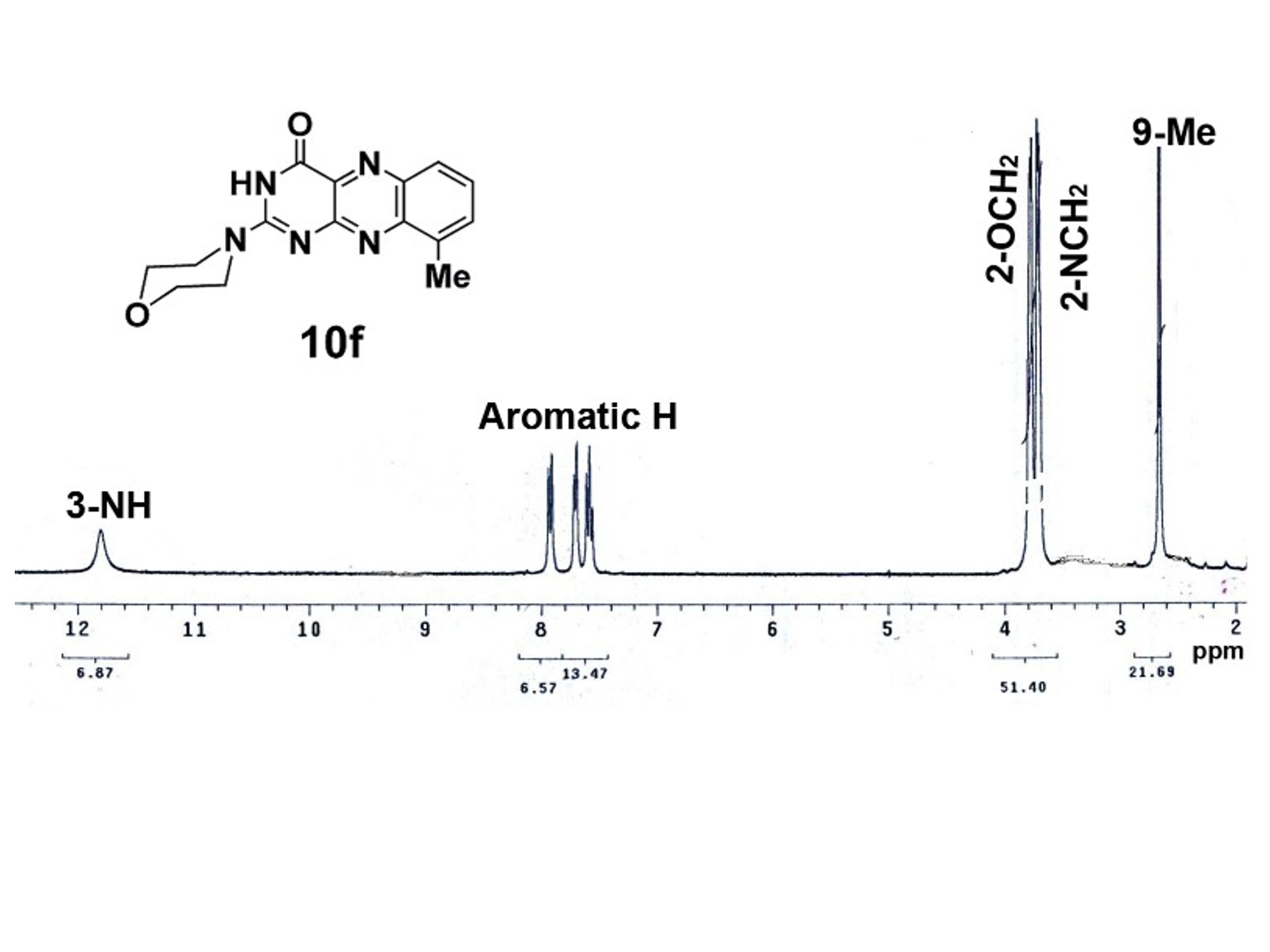


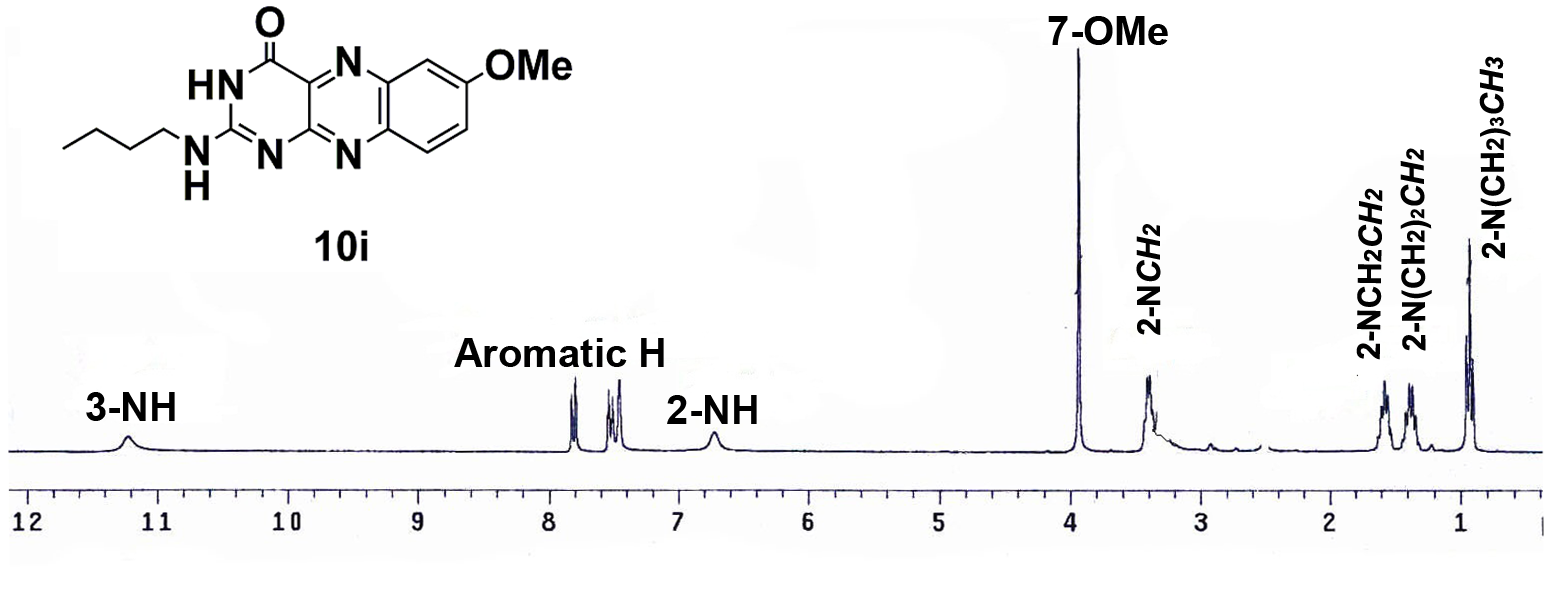


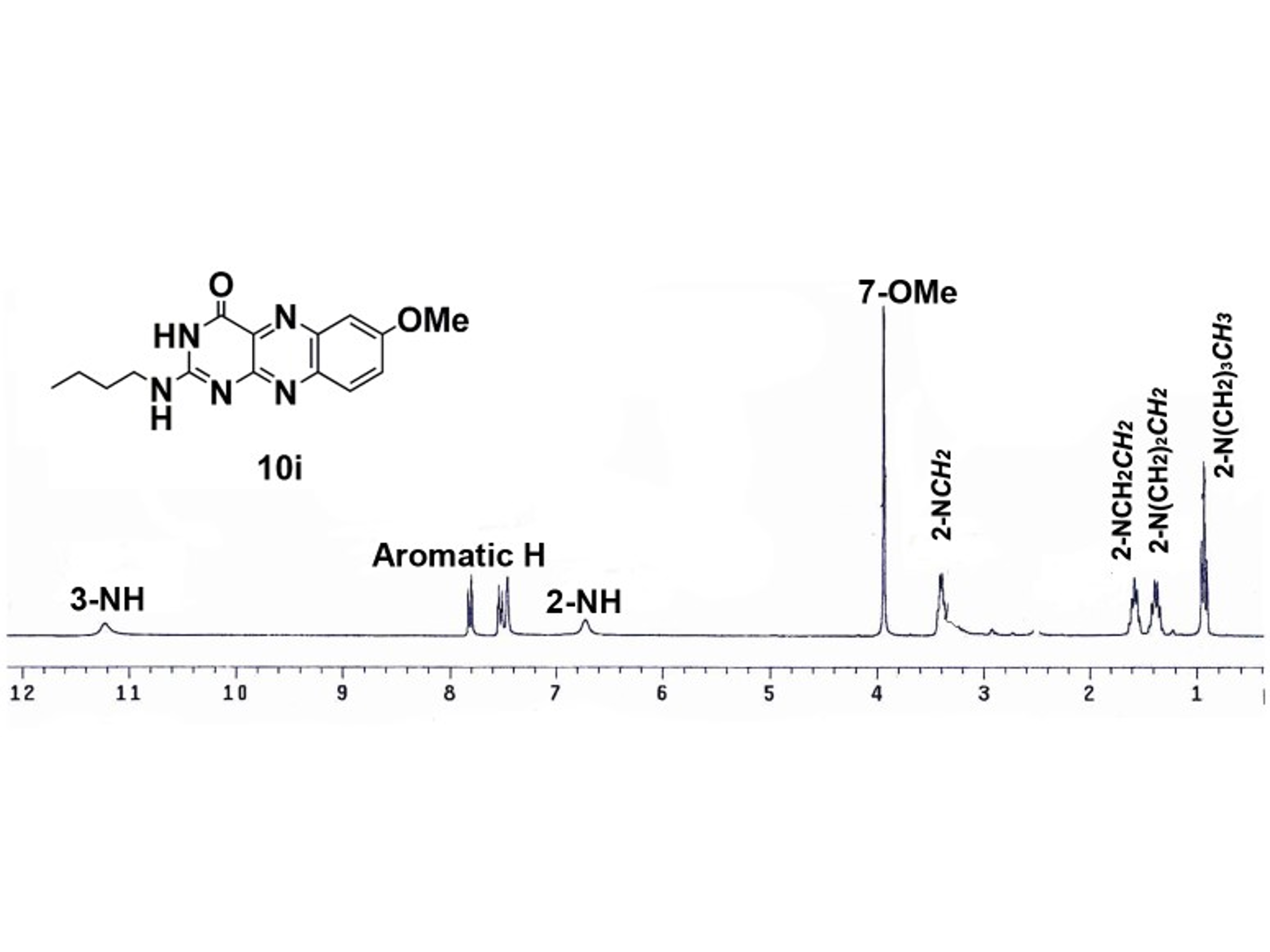


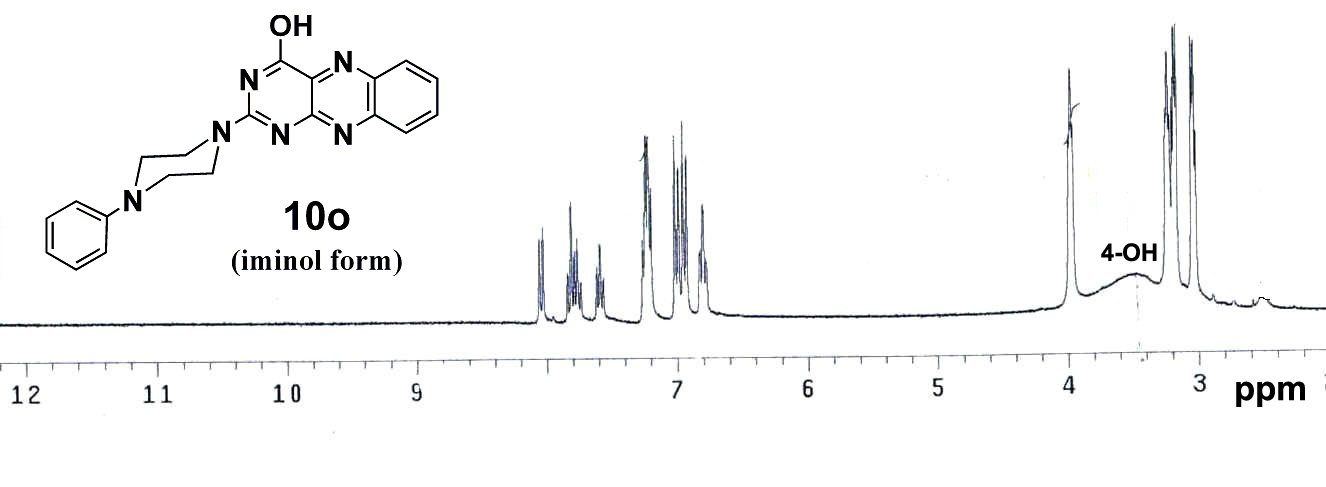

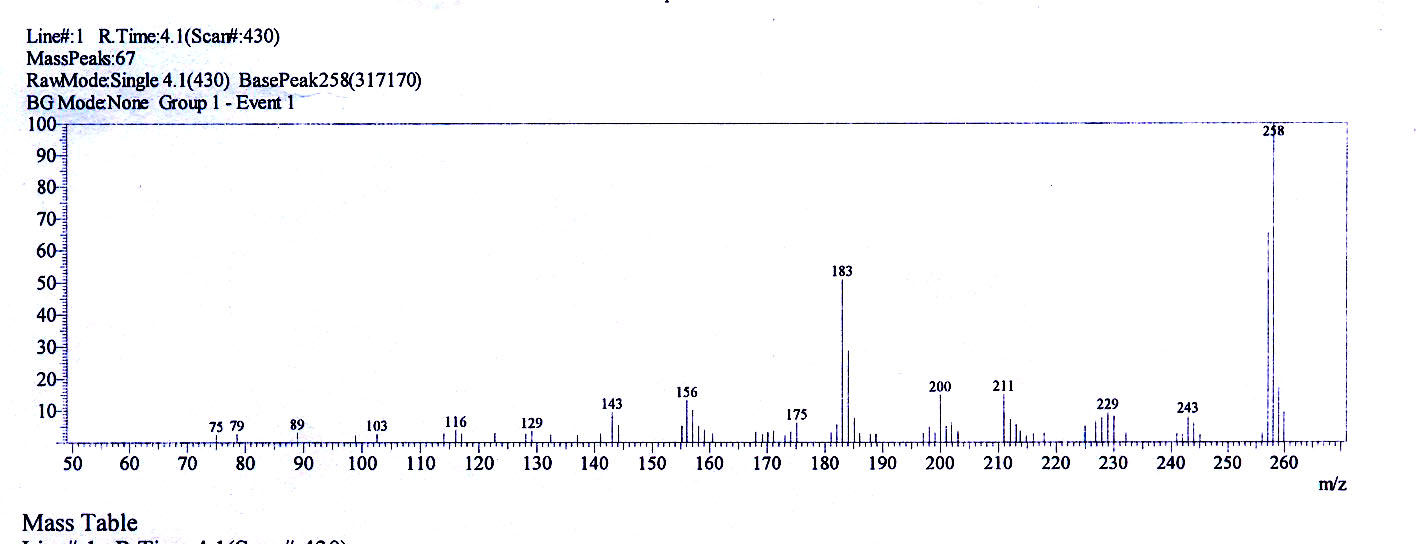


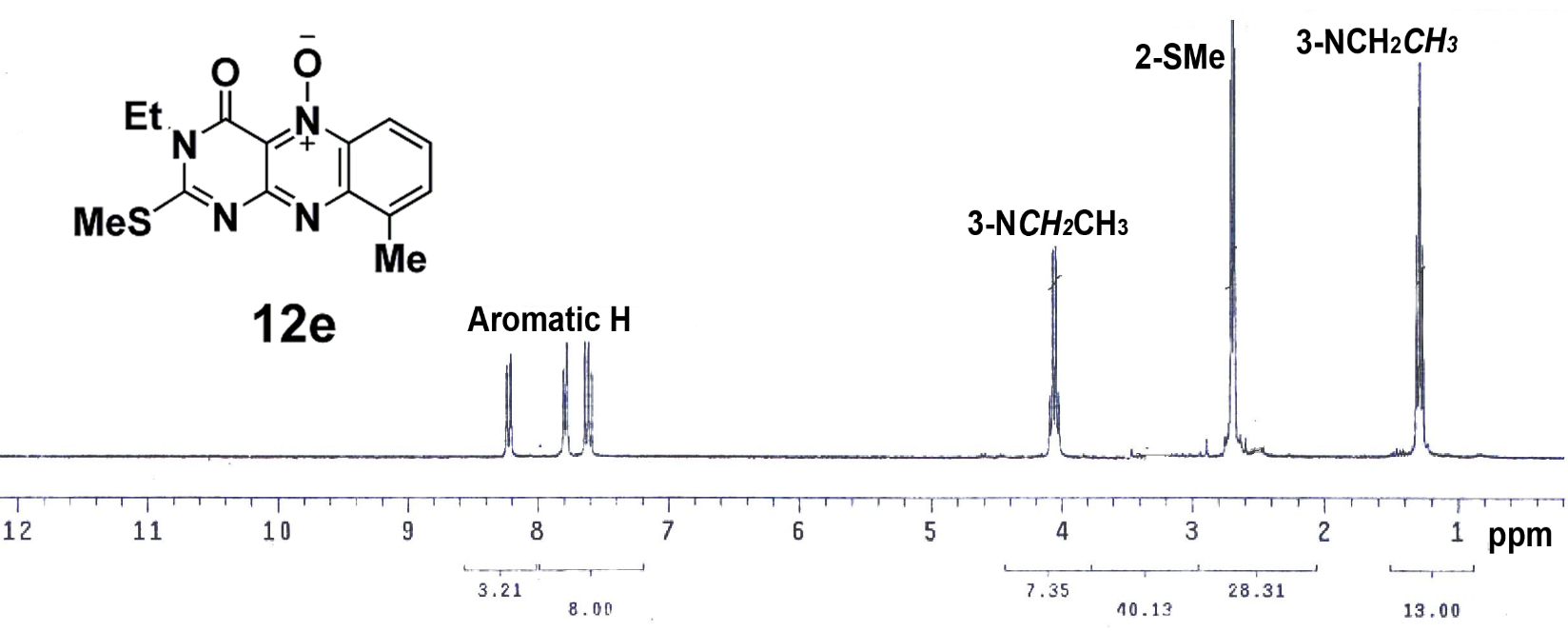


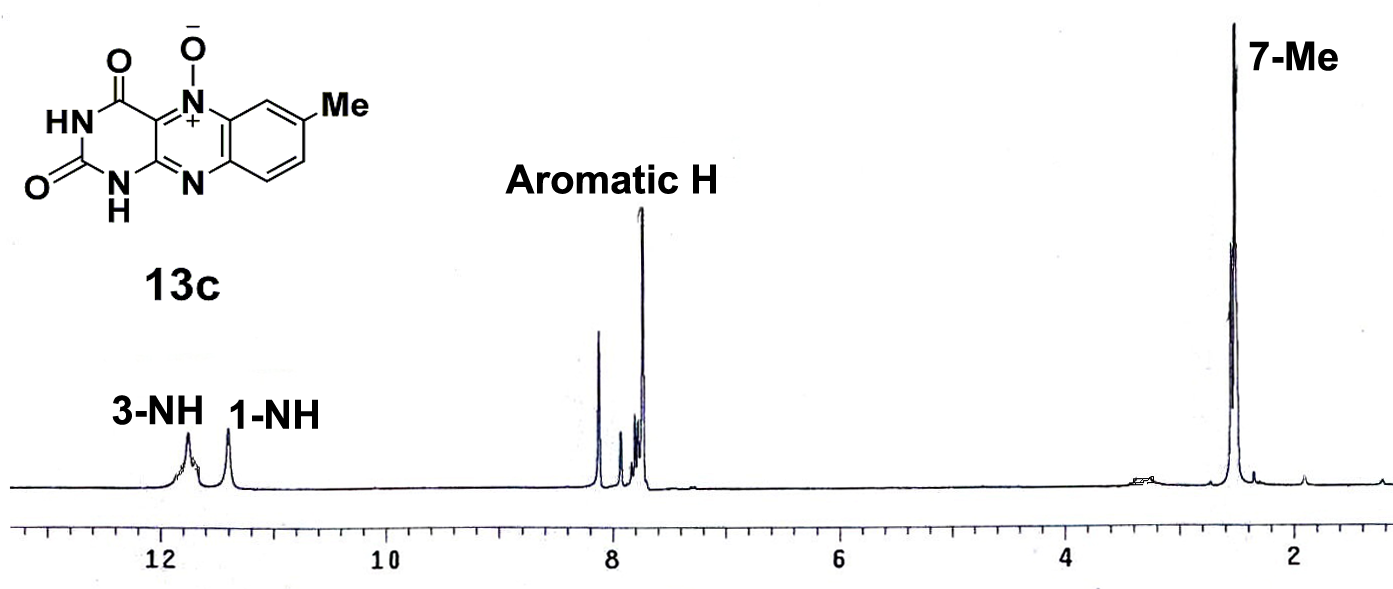


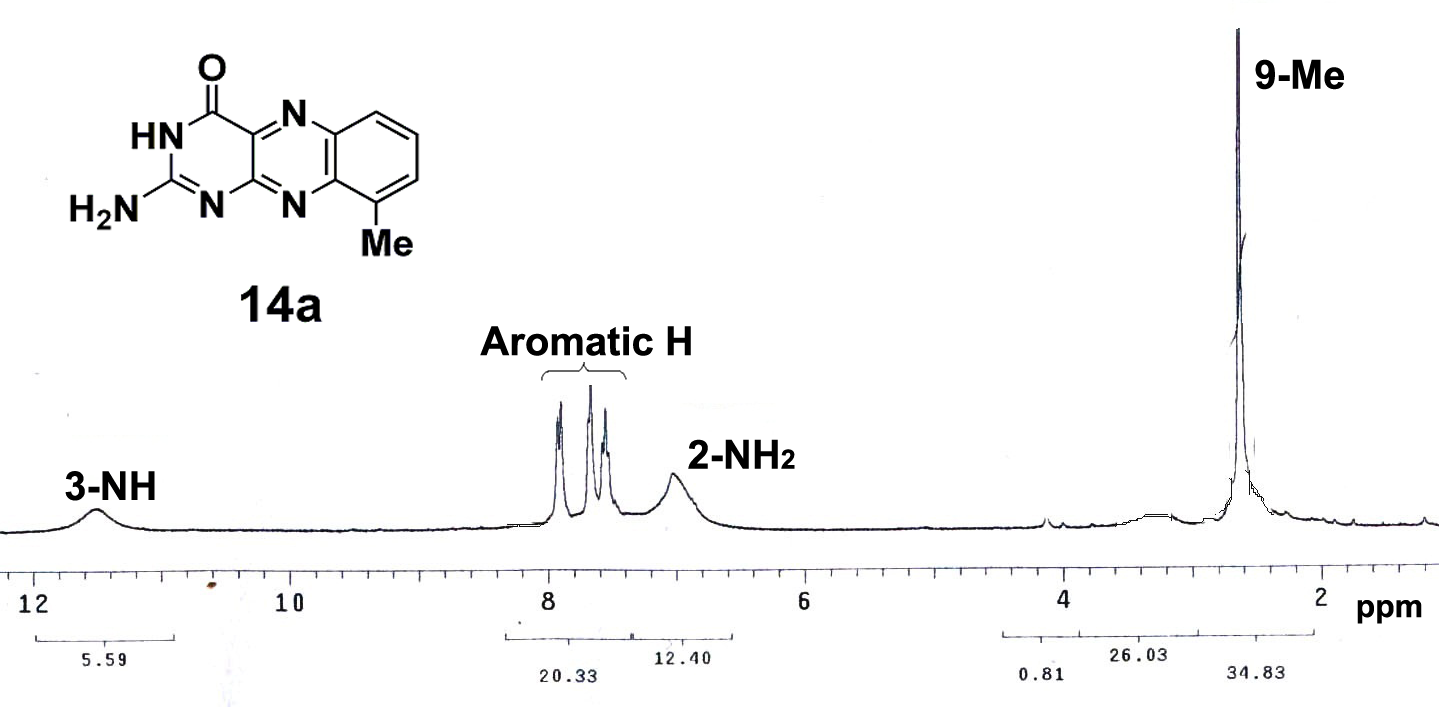


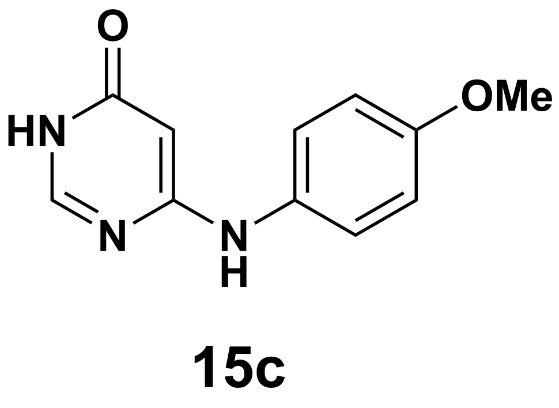


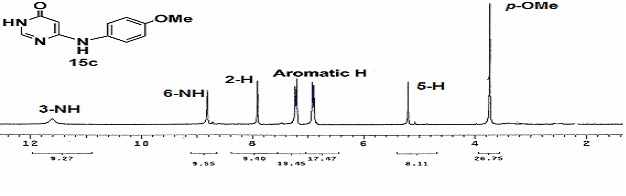


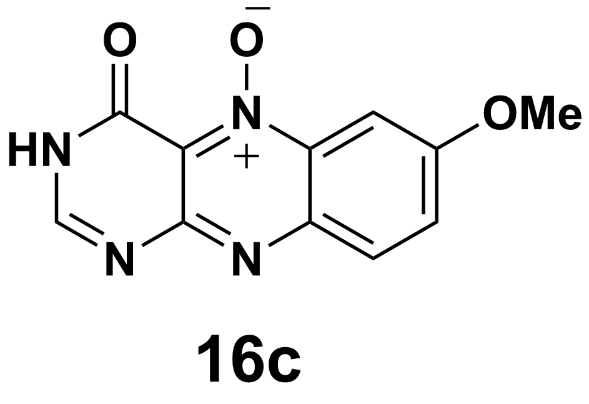


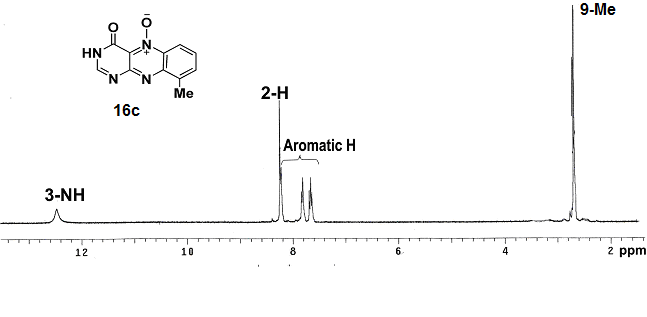


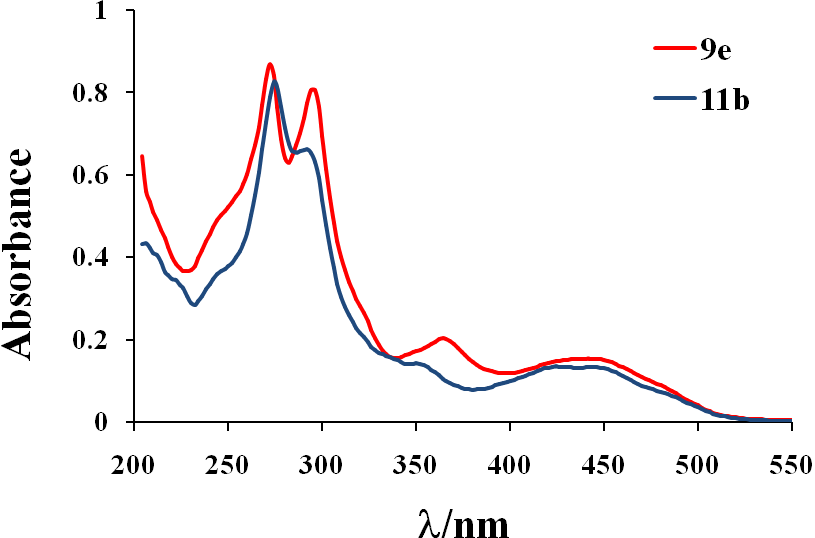


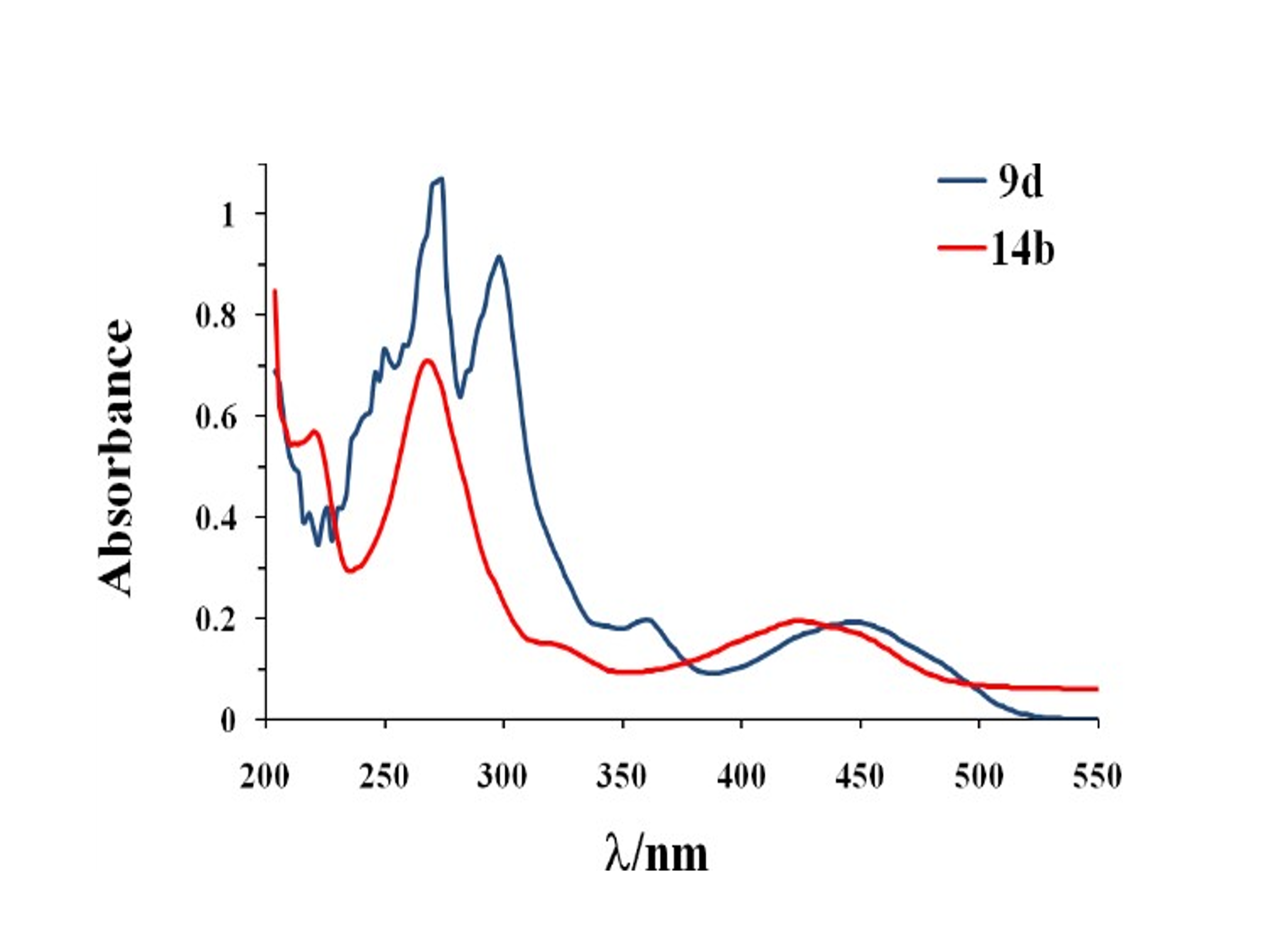


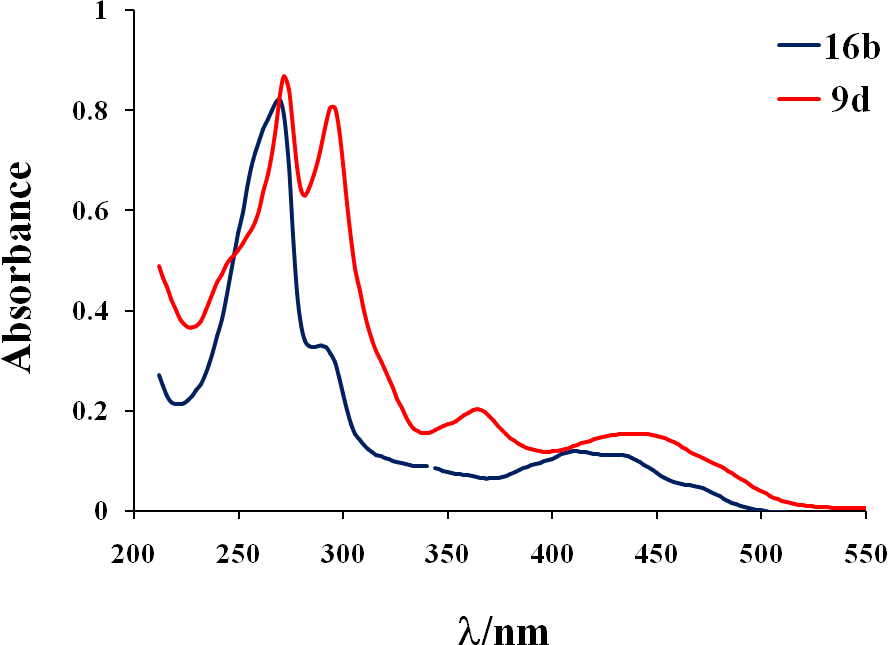


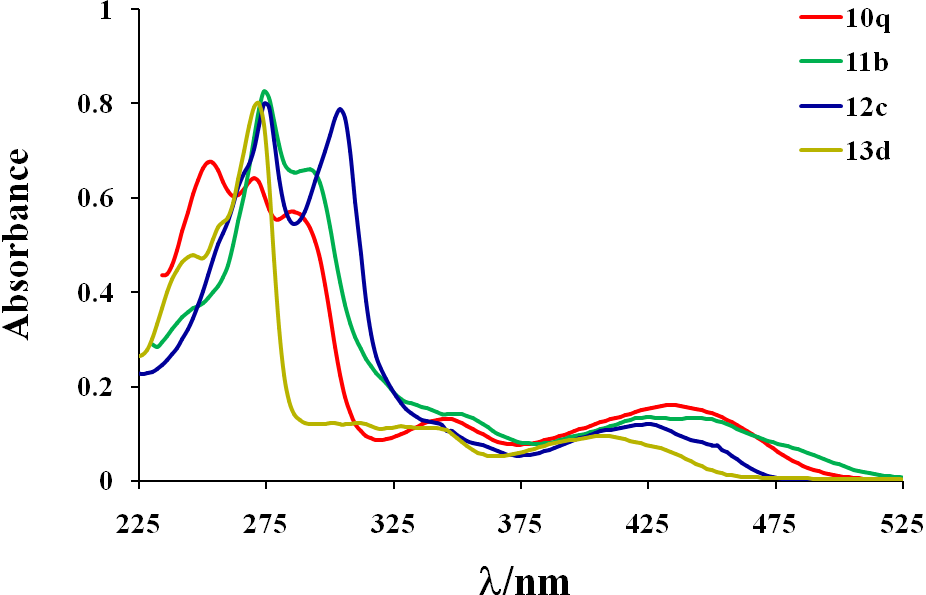


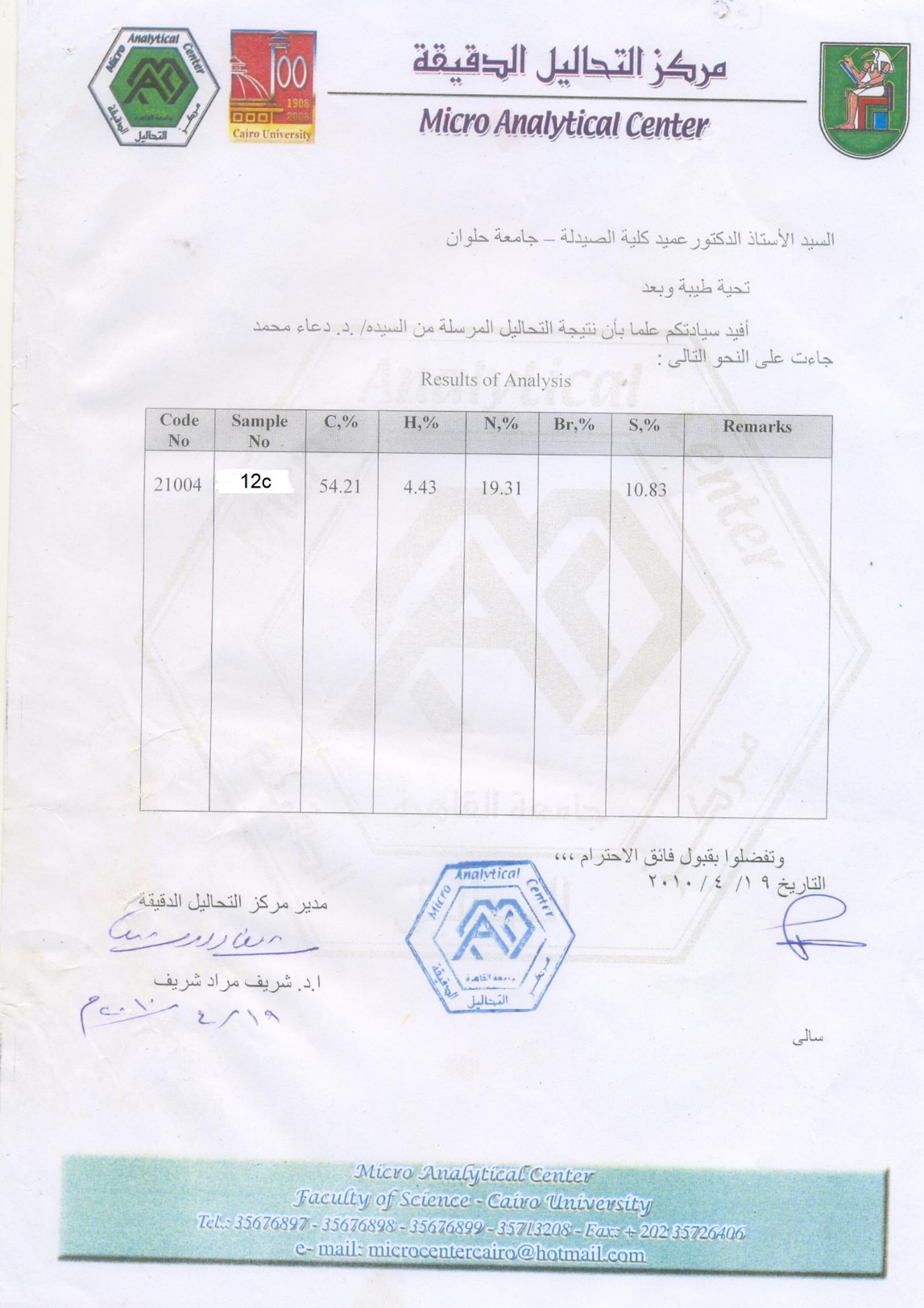


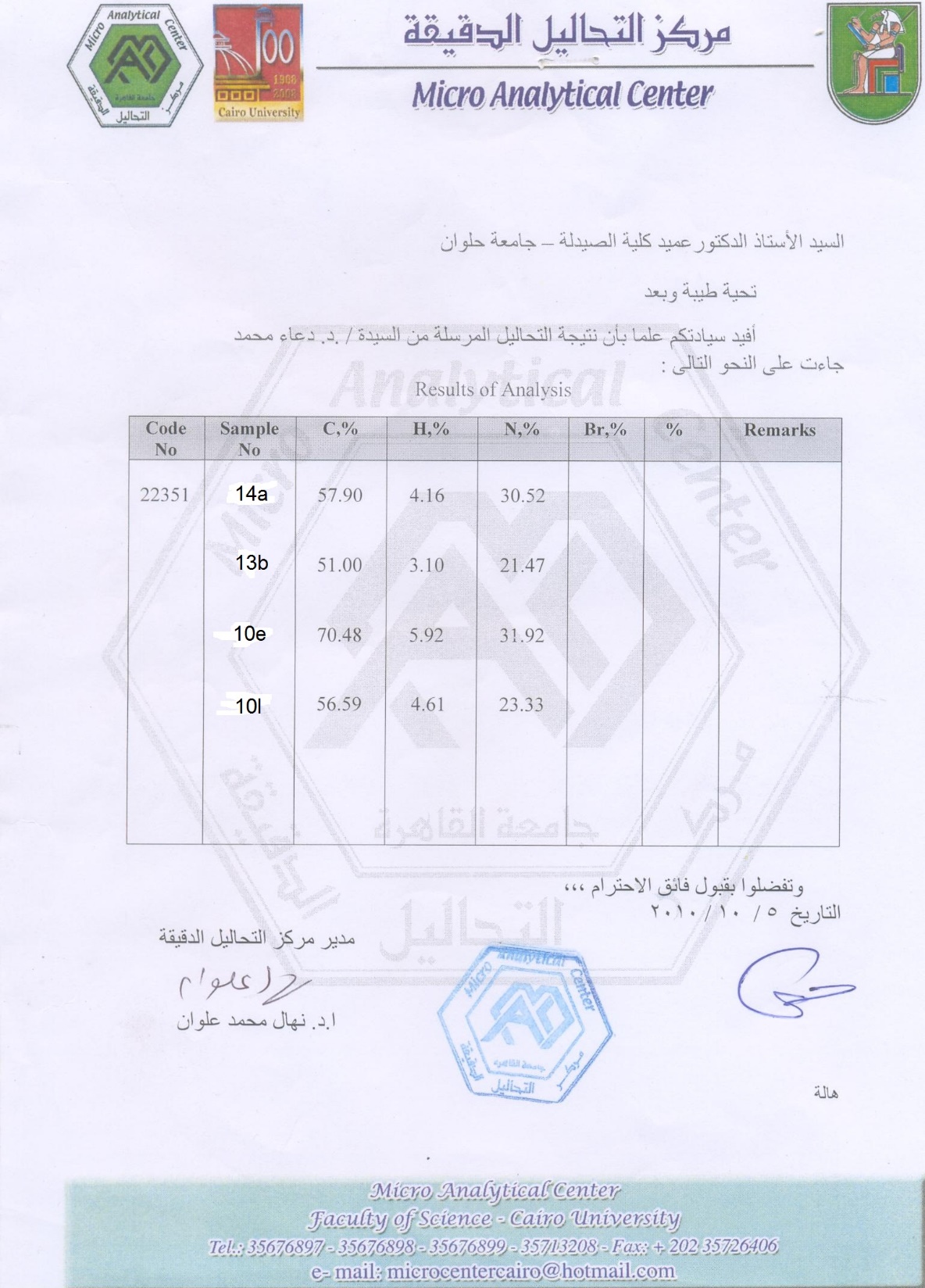


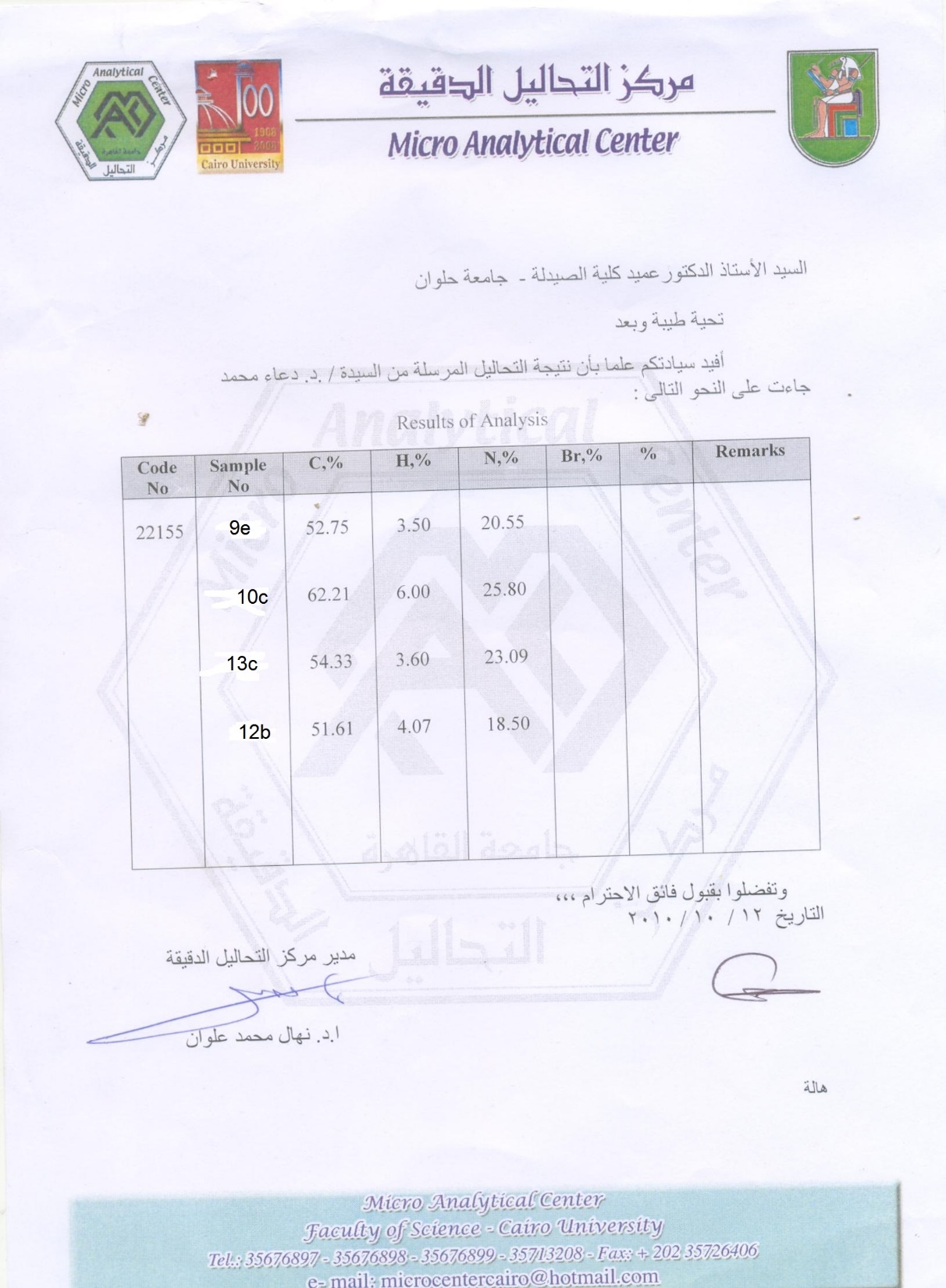


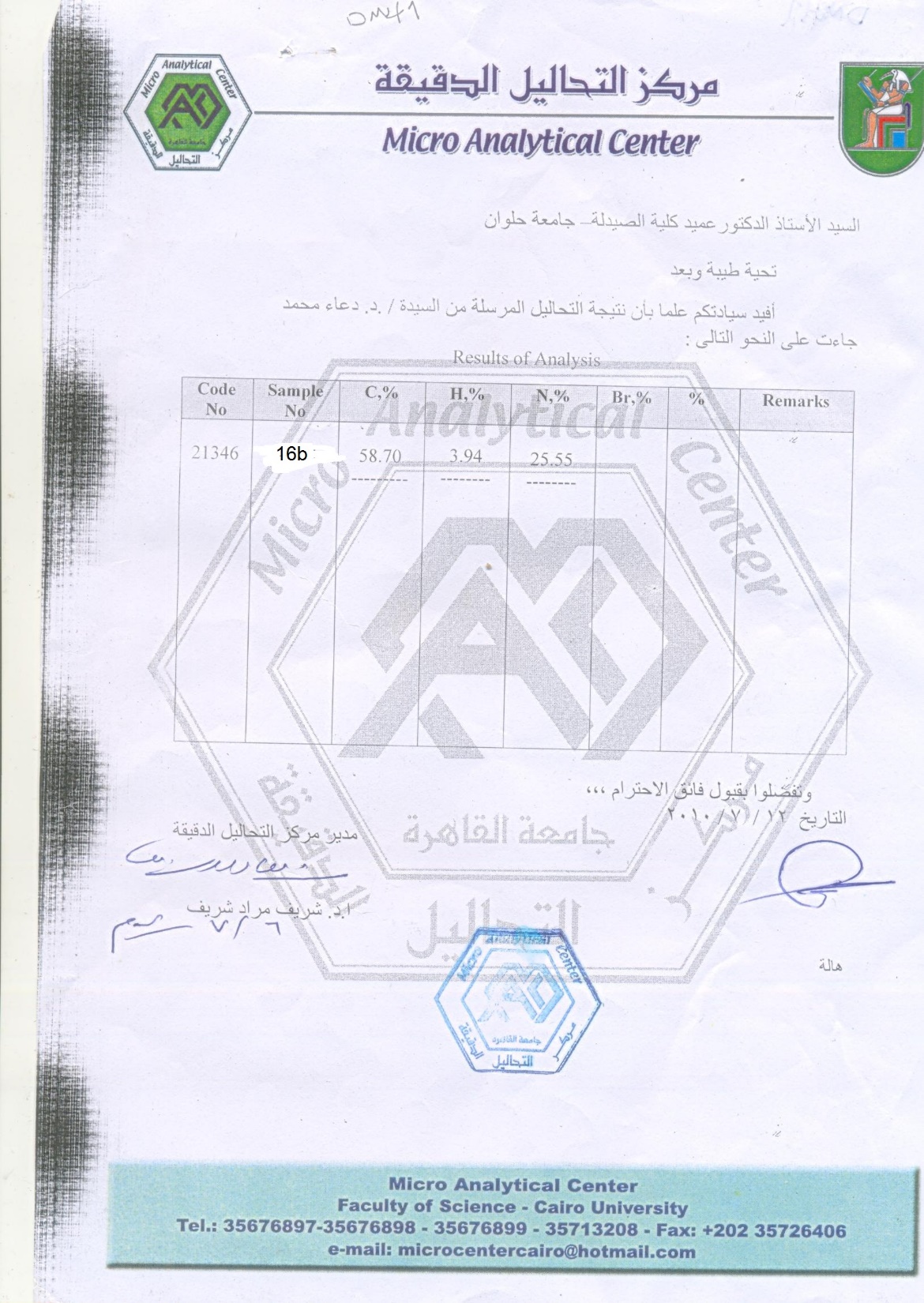


**Protein kinase profiling for compounds DM 8 (10j) and DM 13 (9e)**
